# Long non-coding RNA SNHG9 regulates viral replication in rhabdomyosarcoma cells infected with enterovirus D68 *via* miR-150-5p/c-Fos axis

**DOI:** 10.3389/fmicb.2022.1081237

**Published:** 2023-01-19

**Authors:** Huichao Fu, Junzhuo Si, Lei Xu, Xia Tang, Yonglin He, Nan Lu, Huayi Li, Anlong Li, Sijia Gao, Chun Yang

**Affiliations:** ^1^Department of Pathogen Biology, College of Basic Medicine, Chongqing Medical University, Chongqing, China; ^2^Rongchang District People’s Hospital, Chongqing, China

**Keywords:** enterovirus D68, SNHG9, miR-150-5p, c-Fos, infection, ceRNA

## Abstract

**Background:**

The *Enterovirus* D68 (EV-D68) epidemic has increased knowledge of the virus as a pathogen capable of causing serious respiratory and neurological illnesses. It has been shown that long noncoding RNAs (lncRNAs) regulate viral replication and infection via multiple mechanisms or signaling pathways. However, the precise function of lncRNAs in EV-D68 infection remains unknown.

**Methods:**

The differential expression profiles of lncRNA in EV-D68-infected and uninfected rhabdomyosarcoma (RD) cells were studied using high-throughput sequencing technology. The knockdown through small interfering RNA (siRNA) and overexpression of lncRNA SNHG9 (small ribonucleic acid host gene 9) were applied to investigate how lncRNA SNHG9 regulates EV-D68 propagation. The targeted interactions of lncRNA SNHG9 with miR-150-5p and miR-150-5p with c-Fos were validated using dual luciferase reporter system. LncRNA SNHG9 knockdown and miR-150-5p inhibitor were co-transfected with RD cells. QRT-PCR and western blot were used to detect RNA and protein levels, of c-Fos and VP1, respectively. Median tissue culture infectious dose (TCID50) was applied to detect viral titers.

**Results:**

The results demonstrated that a total of 375 lncRNAs were highly dysregulated in the EV-D68 infection model. In the EV-D68 infection model, lncRNA SNHG9 and c-Fos were increased in EV-D68-infected RD cells. However, the expression level of miR-150-5p was downregulated. In addition, overexpression of SNHG9 in RD cells resulted in decreased viral replication levels and viral titers following infection with EV-D68, and further experiments revealed that overexpression of SNHG9 inhibited the viral replication by targeting increased miR-150-5p binding and significantly increased c-Fos expression in RD cells.

**Conclusion:**

Our findings indicate that the SNHG9/miR-150-5p/c-Fos axis influences EV-D68 replication in host cells and that SNHG9 may be a possible target for anti-EV-D68 infection therapies.

## 1. Introduction

In 1962, EV-D68 was initially isolated from four children with pneumonia and severe respiratory disorders in Berkeley, California, United States (Holm-Hansen et al., 2016). Since its discovery, the virus has been associated with only a few instances of respiratory illness (Holm-Hansen et al., 2016; Xiang and Wang, 2016; Messacar et al., 2016b; Sun et al., 2019). A huge outbreak of EV-D68 that began in the late summer of 2014 in the United States and Canada and was subsequently reported in other countries, caused severe respiratory illnesses, primarily in children (Messacar et al., 2016a, 2016c). Given the magnitude and severity of the 2014 outbreak and the occurrence of severe neurological side effects of acute flaccid paralysis in children, EV-D68 requires additional attention (Bosis and Esposito, 2017; Hu et al., 2020; Andrés et al., 2022). EV-D68 has become a global threat to public health, but there is now neither a vaccine nor an effective antiviral medicine (Imamura and Oshitani, 2015). LncRNAs with more than 200 nucleotides were once assumed to represent transcriptional noise (Nojima and Proudfoot, 2022). Recent studies have shown that lncRNAs affect gene expression and are critical for pathological processes such as cancer and inflammatory response (Chew et al., 2018; Liu et al., 2021; Gysens et al., 2022). In addition, more and more data indicate that the altered lncRNA expression is closely related to the pathogenesis of viral infection (Yin et al., 2013; Aznaourova et al., 2022; Basavappa et al., 2022; Li H. et al., 2022; Liu et al., 2022): The lncRNA ALPHA targets chikungunya virus to limit infection (Basavappa et al., 2022). Single-cell RNA sequencing reveals the nuclear decoy lncRNA PIRAT as a modulator of systemic monocyte immunity during COVID-19 infection (Aznaourova et al., 2022). However, the function of lncRNAs in EV-D68 infection is uncertain.

20–25 nucleotides long, miRNAs control gene expression by attaching to target genes’ 3′ untranslated regions (3′ UTR). They are involved in numerous pathological processes, such as cancer, cell proliferation, and organ development (Shenoy and Blelloch, 2014; Rupaimoole et al., 2016; Treiber et al., 2019). Multiple dysregulated miRNAs, including miR-let-7 and miR-150, are implicated in the mechanism of viral infection (D’Agostino et al., 2022; You et al., 2022). By interacting with mRNAs and miRNAs, lncRNAs play a significant biological role. In addition, the hypothesized lncRNA-miRNA-mRNA regulatory mechanism during EV-D68 infection is unknown and requires investigation.

In this study, the lncRNA expression profile in EV-D68-infected host cells was comprehensively analyzed by high-throughput sequencing technology, and the target genes of lncRNA were predicted according to different mechanisms of gene regulation. Then lncRNA SNHG9 was identified for study, and it was confirmed to affect EV-D68 replication in cells by targeting the miR-150-5p/c-Fos axis. Our findings could provide new strategies for preventing and treating EV-D68 infection.

## 2. Materials and methods

### 2.1. Cell and viruses

Rhabdomyosarcoma (RD) cells were cultured at 37°C in 10% fetal bovine serum (FBS; Hyclone, United States) Dulbecco’s modified Eagle medium (DMEM; Hyclone, United States) in complete medium with 5% carbon dioxide (CO_2_) until cell confluency reached 80–90%. The EV-D68 (Fermon strain) utilized in this work was maintained and propagated on RD cells. Briefly, fused cells were infected with EV-D68 at various multiplicities of infection (MOI) of 1, and the virus was isolated from the supernatant at the relevant infection times.

### 2.2. Virus infection and viral titers evaluation

RD cells were planted in 6-well plates. When the cell density reached 60–80%, EV-D68 inoculum was supplied and incubated at 37°C with 5% CO_2_. The supernatant was changed after 4 h with DMEM containing 2% FBS. Cell samples were obtained at the relevant hours based on the various experimental requirements. Viral titer assays were performed by using the TCID_50_ method. In detail, RD cells were cultured in 96-well plates. When the cell density reached 60–80%, the previous culture media was removed and the 96-well plate was washed twice with PBS. Simultaneously, the EV-D68 virus stock solution was serially 10^−1^ diluted from 10^−10^. At each dilution, the diluted virus was implanted in a single vertical row. The cytopathic effect (CPE) in the 96-well plate was measured every 24 h for 3–5 days, and the TCID_50_ was estimated using Karber’s method. The formula is lgTCID_50_ = *L*–*D*(*S*–0.5), where *L* is the highest dilution logarithm, *D* is the difference between dilution controls, and *S* is the sum of CPE-positive well ratios.

### 2.3. RNA extraction and lncRNA sequencing

Six-well tissue culture plates were inoculated at ~1 × 10^6^ RD cells/well. When cells reach approximately 90% confluence, wash twice with PBS and incubate EV-D68 with cells at MOI of 1. Cells were collected after 0 h of infection and 24 h, and cells treated with EV-D68 for 0 h were designated as the control group. For each time point, total RNA was extracted from three different experiments independently and pooled into one set, following the manufacturer’s instructions (Invitrogen, Carlsbad, CA, United States). Total RNA was then detected and quantified using a nanotitrator and an Agilent 2,100 Bioanalyzer (Thermo Fisher Scientific, MA, United States); all samples containing RNA samples with RIN 8 and 28 s/18 s 1 were considered. cDNA segments containing aptamers were amplified by PCR and then purified using ampoules of XP magnetic beads. For quality control, libraries were analyzed on an Agilent T Technologies 2100 Bioanalyzer. The sequences of splice oligonucleotides were denatured by heating and cycling the previously disclosed double-stranded PCR products. Circular single-stranded DNA was used to create completed libraries (ssCir DNA). More than 300 DNA nanoballs (DNBs) were produced by amplification of the final library with phi29 (Thermo Fisher Scientific, MA, United States). DNBs were included in patterned nanoarrays on the BGISEQ500 platform (BGI, Shenzhen, China) to obtain double-ended 100-base readings (BGI, Shenzhen, China). After filtering the sequencing data with SOAPnuke, clean reads for lncRNAs were acquired and stored in FASTQ format (v1.5.2). HISAT2 (v2.0.4) was used to map clean readings to the reference genome. Using Ericscript (v0.5.5) and RMAT (v3.2.5), differentially spliced genes (DSG) and fusion genes were found. Bowtie2 (v2.2.5) was used to align clean reads to the gene set, a database generated by the Beijing Genome Institute (BGI) in Shenzhen that includes known and new, coding and non-coding transcripts, and then RSEM (v1.2.12) was utilized to calculate gene expression levels. The original sequencing data used in the article, and the original data processing are all consistent with the source of the published articles in our laboratory (Si et al., 2021).

### 2.4. Analysis of differentially expressed lncRNAs

To evaluate transcript expression, Cufflinks software (v2.1.1.) was used to determine transcripts per kilobase and per million mapped read fragments (FPKM) based on fragment length and read counts mapped to these fragments. FPKM 0.1 generally implies that the transcript is expressed. Following the experimental design, we screened for differentially expressed genes (DEG) between EV-D68-infected and control groups using the cufflinks software. | Log_2_ (fold change) | ≥ 1 and *Q*. Value < 0.05 serve as indications for distinguishing between the infected and uninfected groups.

### 2.5. Construction of lncRNA gene regulatory network

The lncRNAs regulate cellular biology by binding competitively to miRNAs as competing endogenous RNAs (ceRNAs), consequently modulating the amounts of encoded proteins (Salmena et al., 2011). The ceRNA network regulatory map was used to predict all possible interactions of the differential lncRNAs, miRNAs, and mRNAs, calculate the correlation of targeting relationships, and visualize them using Cytoscape (v3.8.2). The Coexpression Network (CN) assumes that co-expressed genomes are involved in the same pathway, regulated by the same stimuli, and have similar biological activity (Cen et al., 2022). Based on the Pearson correlation values algorithm, lncRNAs and coding genes were identified. Using modified signal intensities for different mRNA and lncRNA expression levels, co-expression networks were created and displayed with Cytoscape (v3.8.2). LncRNAs can also interact with mRNAs through base complementary pairing, and the functions of lncRNAs were investigated by targeting mRNAs of lncRNAs (Li M. et al., 2022). The software lncTar is employed to predict the targeting relationships of lncRNAs and all the differential mRNAs. All the reciprocal pairs predicted to have targeting relationships are drawn and visualized using Cytoscape (v3.8.2). In addition to interacting with some RNAs by a complementary pairing of base sequences, lncRNAs can also interact with RNA-binding protein (RBP) *via* specific spatial structures (Wang et al., 2020). The database RBPDB was used, all possible target proteins of the predicted top five lncRNA of up-and down-regulated differential ploidy (without differential screening). All interaction pairs with target relationships are plotted and the network diagram is constructed *via* Cytoscape (v3.8.2).

### 2.6. Gene ontology annotations and KEGG pathways

This study used Gene Ontology (GO) and Kyoto Encyclopedia of Genes and Genomes (KEGG) pathways based on the Database for Annotation, Visualization, and Integrated Discovery (DAVID) to investigate the potential functions of target gene mRNAs derived from the prediction of differentially expressed lncRNAs. Gene ontology keywords give information regarding biological processes (BP), cellular components (CC), and molecular functions (MF), whereas KEGG analysis is utilized to investigate the pathways and functional categories associated with target genes.

### 2.7. Vector construction and cell transfection

The miR-150-5p inhibitor mimics as well as the corresponding negative controls (NC) were obtained from RIBOBIO Co., Ltd. Small interfering RNAs for SNHG9 (si-SNHG9) were purchased from RIBOBIO Co., Ltd. (Guangzhou, China; Table 1). All the plasmids, miRNA inhibitors, mimics and siRNAs were transfected into cells using Lipofectamine 2000 reagent (Invitrogen, United States) following the manufacturer’s instructions.

**Table 1:** Long noncoding RNAs (lncRNAs) SNHG9 siRNA sequence.

Name	Sequences (5′ → 3′)
Human-SNHG9-siRNA-1-F	GAATCTACGTCACCCGAAA
Human-SNHG9-siRNA-1-R	UUUCGGGUGACGUAGAUUC
Human-SNHG9-siRNA-2-F	CCTCTTCACTTAGGACACT
Human-SNHG9-siRNA-2-R	AGUGUCCUAAGUGAAGAGG
Human-SNHG9-siRNA-3-F	GAAGAGTGGCTATAAACGT
Human-SNHG9-siRNA-3-R	ACGUUUAUAGCCACUCUUC
NC-siRNA-F	GGCUCUAGAAAAGCCUAUGCdTdT
NC-siRNA-R	dTdTCCGAGAUCUUUUCGGAUACG

### 2.8. Quantitative reverse transcription polymerase chain reaction validation

The Mir-X miRNA First-Strand synthesis kit (TaKaRa, China) was used for the reverse transcription of total RNA. PrimeScript^™^ RT kit (TaKaRa, China) containing gDNA scavenger was used for reverse transcription of total RNA. The mRQ 3′ Primer supplied with the kit is the 3′primer for miRNA RT-qPCR. Tsingke Biotechnology Co., Ltd. manufactured qualified primers (Table 2). The expression levels of lncRNAs and mRNAs were normalized using GAPDH, whereas the expression levels of miRNAs were adjusted to U6. The 2^–△△Ct^ equation was used to determine the relative expression levels of various RNAs.

**Table 2:** Oligonucleotide primer sets for qRT-PCR.

Gene name	Sequences (5′ → 3′)
BORCS7-ASMT F	TTCCTTCCGCTTTGCTTGTC
BORCS7-ASMT R	GCGCGATTACGTCAGTACCA
SLMO2-ATP5E F	GCAGCGTTTGCAGAGAAGTG
SLMO2-ATP5E R	GTGCCCACACATCTTCACCT
ITGA6-AS1 F	GCGGCATCCCAGTATGATTC
ITGA6-AS1 R	CTCAGCTCCAGCCTTGTTTC
MIR133A1HG F	AGGCAATAAACCACCAAGGG
MIR133A1HG R	TGCCAAAGGTCATCTGTTCA
MEG8 F	AGCACACAAACCATTGCGAG
MEG8 R	GCTCCCAATCAAGAGGCAGA
LINC01970 F	GAGGATCGGAAACAGCCGAG
LINC01970 R	AACAAAGGAGCACTGAGCGA
TALAM1 F	GGTCCCTCTCTCTGGTAGGTCT
TALAM1 R	AGCATCTGGCTGTTGGAAGGA
SNHG9 F	TACGTCACCCGAAAAGCGAC
SNHG9 R	CGCGGACGTTTATAGCCACT
GAPDH F	CCAGGTGGTCTCCTCTGA
GAPDH R	GCTGTAGCCAAATCGTTGT
VP-1 F	GTGGTCCCTAGTCTAAATGCAG
VP-1 R	TAACGTCTCCGACACACCATG
miR-150-5p F	TCTCCCAACCCTTGTAC
miR-150-5p R	GTCGTATCCAGTGCAGGGTCCGAGGT
U6 F	CTCGCTTCGGCAGCACATATACT
U6 R	ACGCTTCACGAATTTGCGTGTC
c-Fos F	CTCCCGAAGAGGAGGAGAAG
c-Fos R	GGACTGATTTTGCACACAGG

### 2.9. Western blot

The total protein from treated cells was extracted by the Total Protein Extraction Kit (Thermo Fisher Scientific, Waltham, MA, United States) according to the manufacturer’s instructions. Protein concentration in the lysate was measured using the Protein BCA Assay Kit (Beyotime, Shanghai, China). On 10% sodium dodecyl sulfate-polyacrylamide gel electrophoresis, proteins were separated and then transferred to PVDF membranes for protein blotting detection. The membranes were sealed for 1 h at room temperature with 5% skim milk in Tris-buffered saline with Tween 20. The primary antibodies were incubated with EV-D68 VP1 (1:5000, GeneTex, Shanghai, China) and GAPDH (1:8000, Proteintech Group, Wuhan, China) for 1 h at 4°C, followed by horseradish peroxidase-conjugated secondary antibodies. The target protein was monitored and quantified using the Bio hypersensitive Rad ECL chemiluminescence kit (Suzhou New Cell & Molecular Biotechnology Co., Ltd., Suzhou, China). ImageJ software (National Institutes of Health) was utilized for the quantitative analysis of proteins.

### 2.10. Dual-luciferase reporter assay

A luciferase reporter plasmid containing wild-type (WT) or mutant (MUT) SNHG9/c-Fos with miR-150-5p binding site was constructed. Different promoter sequences containing WT or MUT BS were cloned into the pmiRGLO vector (Promega, Madison, WI, United States) and transfected into target cells using Lipofectamine 3,000 (Invitrogen). Samples were assayed for Luciferase activity using Promega’s Dual-Luciferase Reporter Assay System (E1910). At 48 h post-transfection, the values were read using a GloMax bioluminescence detector for 2 s.

### 2.11. Statistical analysis

GraphPad Prism version 8.0 (GraphPad Software, San Diego, CA, United States) was used to conduct all statistical analyses. Student’s t-test was used to compare two groups, and a one-way analysis of variance was followed by Tukey’s *post hoc* test to compare multiple groups. *p* < 0.05 was determined to be statistically significant.

## 3. Results

### 3.1. Establishing an *in vitro* model of infected EV-68 cells

**Figure 1:**
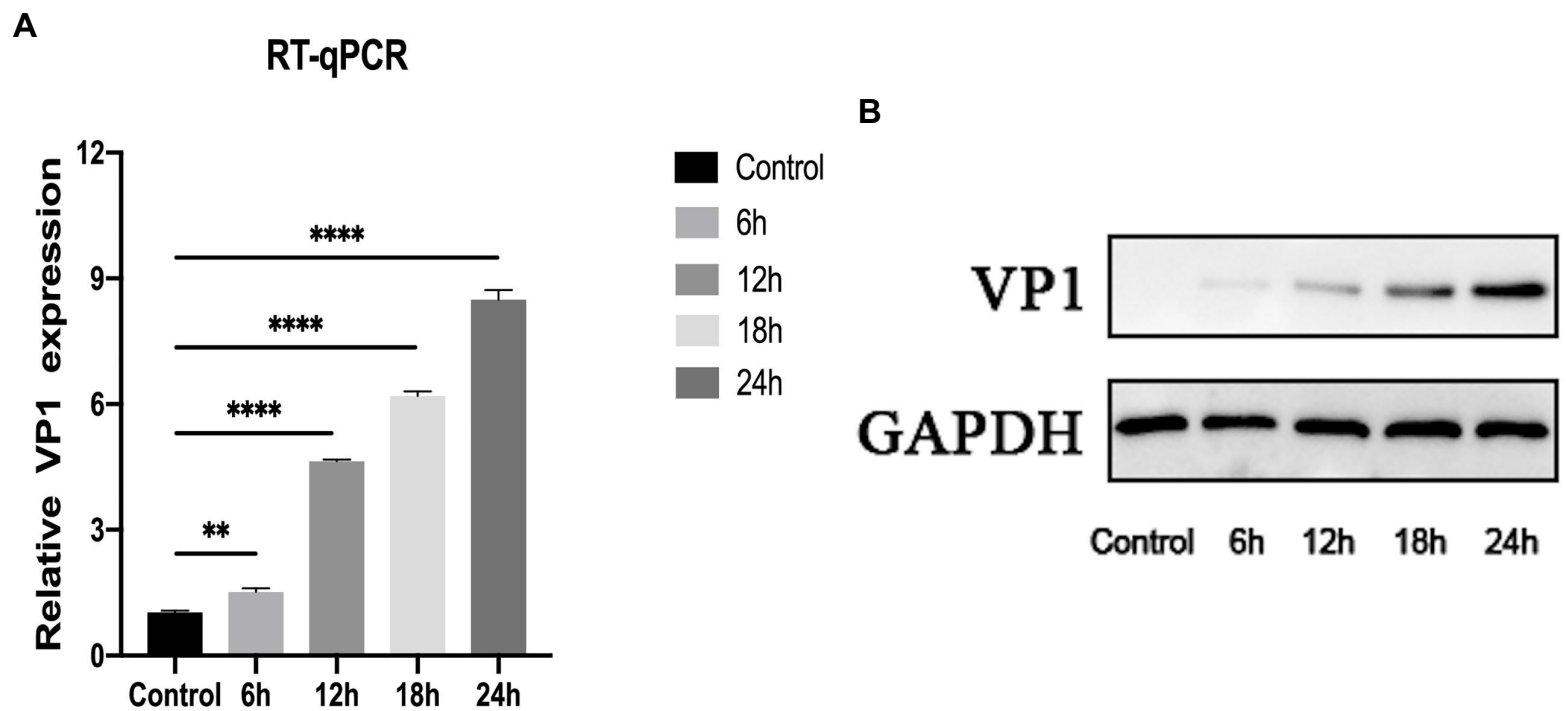
Construction of EV-D68 infected cell model. **(A)** The relative expression level of viral VP1 was determined using quantitative reverse transcription polymerase chain reaction (qRT-PCR). Rhabdomyosarcoma (RD) cells were infected with EV-D68 for 0, 6, 12, 18, and 24 h respectively, and the total RNA was extracted. **(B)** The expression level of viral VP1 was determined by western blot. The total protein in RD cells was collected, which had been infected with EV-D68 for 0, 6, 12, 18, and 24 h, respectively (^**^p < 0.01, ^****^p < 0.0001).

As depicted in Figures 1A,B, qRT-PCR and western blot were utilized to determine the expression level of VP1 at MOI = 1 after 0, 6, 12, 18, and 24 h of RD cell infection. Both RNA and protein levels of VP1 expression increased considerably with increasing infection time, demonstrating a time dependence. In a conclusion, RD cells are an excellent model for understanding the EV-D68 infection process.

### 3.2. Analysis of differentially expressed lncRNAs

**Figure 2:**
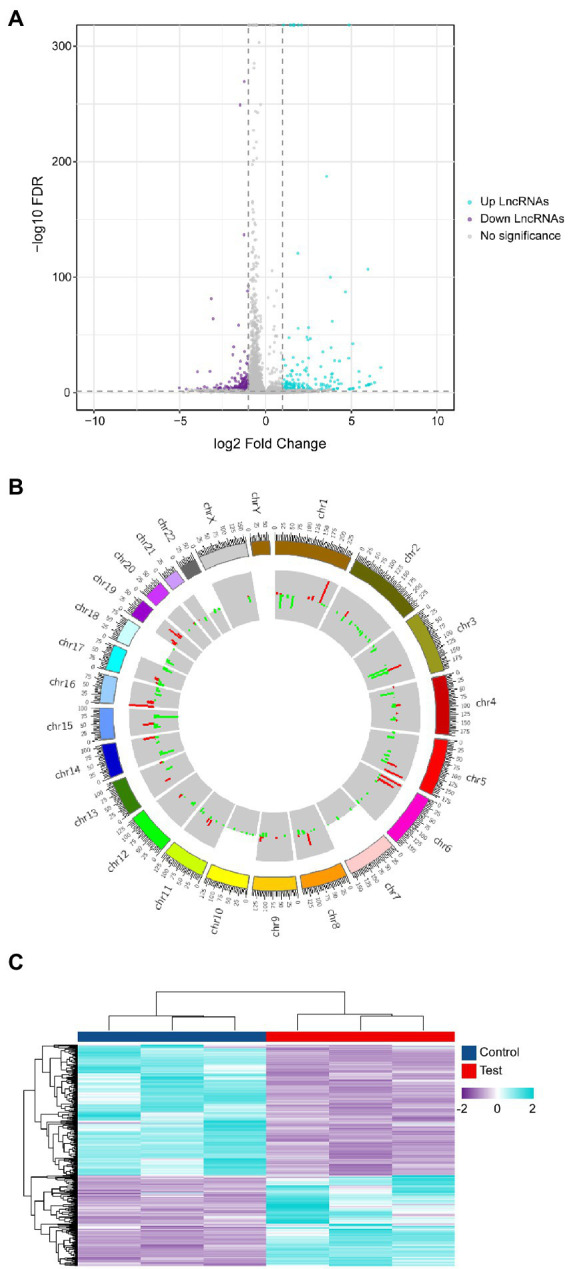
Differential analysis of lncRNA expression profiles. **(A)** Differential lncRNAs volcano plot: the plot’s horizontal and vertical axes reflect Log_2_(Fold change) and -Log_10_(Q. Value), respectively. We determined the importance of Log_2_ | (Fold change) | ≥ 1, Q. Value < 0.05 as the cut-off criterion. **(B)** Localization of differential lncRNAs chromosomes: the inner and outer circles of the circos plot were used to represent up-regulated and down-regulated genes, respectively, with |Log_2_(Fold change) | ≥ 1, Q. Value < 0.05 as the cut-off criterion and the fold change in expression was represented by column height. **(C)** Heat map of expressed lncRNAs cluster analysis: each column represents control and experimental groups The horizontal axis symbolizes each column of experimental groups. The hue indicates the expression level of each sample group. The tint transitions from violet to blue as the level of expression increases.

Since differentially expressed genes will be utilized as the basis for further analysis in high-throughput whole transcriptome sequencing, screening for differentially expressed genes is critical. The entire dataset (Supplementary Data Sheet 1) was filtered using the following criteria. (|Log_2_(Fold change) | ≥ 1, *Q*. Value < 0.05). Differential expression analysis plotted in the volcano plot (Figure 2A) shows that 375 differentially expressed lncRNAs were found in the infected group compared to the control group, of which 154 were upregulated and 221 were downregulated. Mapping the chromosomal localization of these lncRNAs indicated that the majority of the highly differently expressed lncRNAs originated from the autosomal chromosome (Figure 2B). In addition, these lncRNAs were utilized for clustering analysis, which revealed the expression pattern of lncRNAs between various samples (Figure 2C). These findings imply that EV-D68 infection generates alterations in several lncRNAs and that lncRNAs co-expressed differentially during EV-D68 infection may be regulatory factors in the pathogenesis of EV-D68.

### 3.3. Prediction of lncRNA target genes and mapping of network regulation

As the mode of action of lncRNAs is very complex, so the target gene prediction must be based on the various modes of action of lncRNAs.

**Figure 3:**
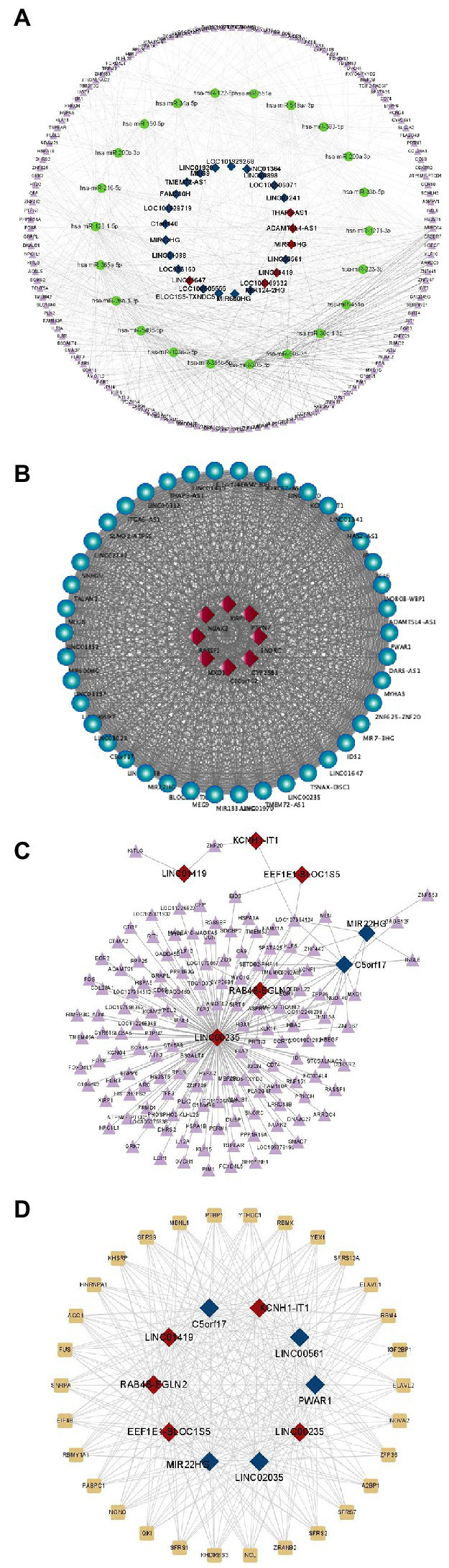
Prediction of differential lncRNAs regulation. **(A)** Differential regulatory network for ceRNAs centered on lncRNAs: This diagram depicts lncRNAs, miRNAs, and mRNAs as squares, circles, and triangles, respectively. Red and dark blue denote the up- and down-regulated lncRNAs, respectively. **(B)** The co-expression network of lncRNAs and protein-coding genes is constructed in Cytoscape by employing Pearson correlation coefficients. The dots and diamonds indicate lncRNA and mRNA, respectively. **(C)** Triangles and squares, representing mRNAs and lncRNAs, respectively, depict anticipated up- and down-regulated differential fold top five lncRNAs and targeting relationships of all differential mRNAs, along with all reciprocal pairs predicted to have targeting associations. **(D)** Network regulation of interactions between lncRNAs and RNA-binding proteins: all potential target proteins of the top five predicted up- and down-regulated differential fold lncRNAs, and all predicted pairs of interactions with targeting relationships, with rectangular and square nodes representing mRNA and lncRNA, respectively.

As shown in Figure 3A, we screened a total of 25 lncRNAs, 22 miRNAs, and 143 mRNAs from differentially expressed genes (Supplementary Data Sheet 2) to develop a regulatory map for the ceRNA network. And the building of a regulatory map for the ceRNA network provides a new perspective for transcriptome research and a more comprehensive and in-depth explanation of certain biological occurrences in RD cells infected with EV-D68. As shown in Figure 3B, 8 mRNAs co-expressed with differential lncRNAs were obtained using expression profiling data (Supplementary Data Sheet 3), and coregulated lncRNAs and mRNAs of the experimental samples were mapped. These co-expressed differential mRNAs and lncRNAs may participate in similar EV-D68 infection pathways.

According to the lncRNA regulatory mechanism, the five most substantially differently expressed lncRNAs were screened, and the differential mRNAs that were complementarily associated with them were predicted (Supplementary Data Sheet 4). Many divergent mRNAs addressed lnc00235, as seen in Figure 3C, indicating that lnc00235 may be a crucial hub lncRNA in the control of EV-D68 infection. LncRNAs perform various functions by forming RNA-protein complexes with proteins such as chromosomal regulatory complexes, transcription factors, and RNP complexes. The lncRNAs with the most significant up- and down-regulation were chosen for RBP prediction (Supplementary Data Sheet 5), as shown in Figure 3D. The SR protein family (SFRS) had the closest targeting to lncRNAs. The most extensively investigated function of this RBP family is its participation in the shearing of RNA and the metabolic processes of mRNAs, including stability and exonucleation (Wan et al., 2022). This also shows that *via* binding to lncRNAs, SFRS may be involved in controlling EV-D68 infection.

### 3.4. lncRNA predicted target genes for KEGG, GO pathway analysis

**Figure 4:**
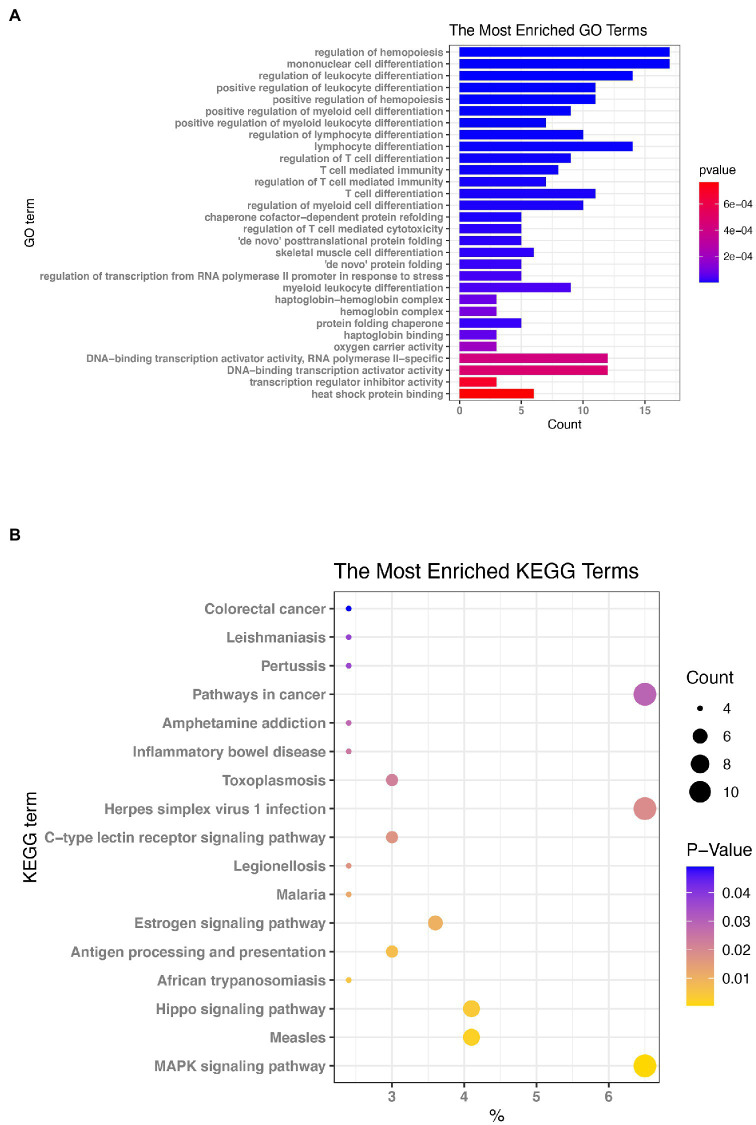
Enrichment evaluation **(A)** GO enrichment: the graph depicts the ranking of the most enriched pathways, with the number of genes enriched in each GO annotation represented by the horizontal bar and the size of Log_10_ (*p*. value) reflected by the bar’s color. **(B)** Enrichment of Kyoto Encyclopedia of Genes and Genomes (KEGG) signaling pathways: The size of the bubbles in the graph represents the number of genes enriched in each signaling route, while the color of the spots indicates their significance level.

To determine the key functions and pathways of action of differentially expressed mRNAs in the EV-D68 infection model, GO and KEGG enrichment analyses were performed on differential mRNAs screened by lncRNA regulatory mechanisms (Supplementary Data Sheet 6). First, as shown in Figure 4A, the most important pathways enriched by BP, CC, and MF in GO enrichment analysis were “Hematopoietic regulation,” “Monocyte differentiation,” “Rhodopsin-hemoglobin complex,” “Hemoglobin complex” and “DNA-binding transcriptional activator activity, RNA polymerase II specificity,” respectively.

The KEGG pathway enrichment analysis (Figure 4B) revealed that the signaling pathways were predominantly enriched (Supplementary Data Sheet 7) in the “MAPK signaling route,” “Measles,” “Hippo signaling pathway,” and “Herpes simplex virus 1 infection.” Based on the results of KEGG and GO enrichment analyses, we will also study the functions of the genes that are enriched in these pathways.

### 3.5. Quantitative reverse transcription polymerase chain reaction validates the accuracy of sequencing results

**Figure 5:**
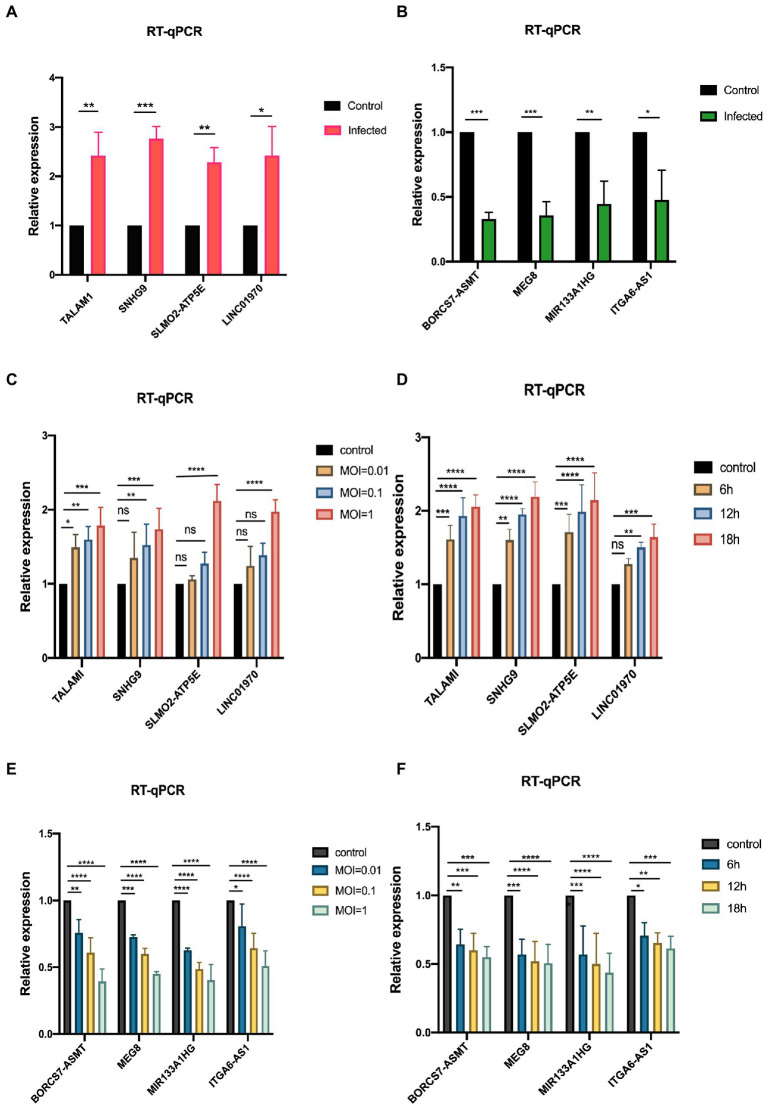
Validation of sequencing data by qRT-PCR **(A,B)** qRT-PCR validation of the screened up-and down-regulated genes, establishing the control group (MOI = 0) and the infected group (MOI = 1). **(C,D)** qRT-PCR was performed to verify the expression variations of up-regulated genes in RD cells after infection with EV-D68 at concentration gradients (MOI = 0.01, 0.1, 1) for 24 h and time gradients (6 h,12 h,18 h post-infection), MOI = 1. **(E,F)** qRT-PCR was used to verify the RNA levels of down-regulated genes at the concentration gradient and time gradient, respectively (^*^p < 0.05, ^**^p < 0.01, ^***^p < 0.001, ^****^p < 0.0001).

To ensure the accuracy of the sequencing results and the reliability of the subsequent data analysis, 8 lncRNAs were randomly selected from 375 differentially expressed lncRNAs for qRT-PCR validation, including 4 up-regulated lncRNAs (TALAM1, SNHG9, SLMO2-ATP5E, and LINC01970) and 4 down-regulated lncRNAs (BORCS7-ASMT, MEG8, MIR133A1HG, and ITGA6-AS1), as shown in Figures 5A,B, at MOI = 1, infected RD cells for 24 h. Moreover, as depicted in Figures 5C,D, it was discovered that the expression levels of these up-regulated genes rose dramatically with time and concentration. Figures 5E,F demonstrate that with the prolongation of infection time and the increase of MOI, the expression level of down-regulated genes decreased gradually. All these results indicate the dependability of high-throughput sequencing data and give prediction data and experimental viability for future mechanistic research.

### 3.6. lncRNA SNHG9/miR-150-5p/c-Fos axis validation

**Figure 6:**
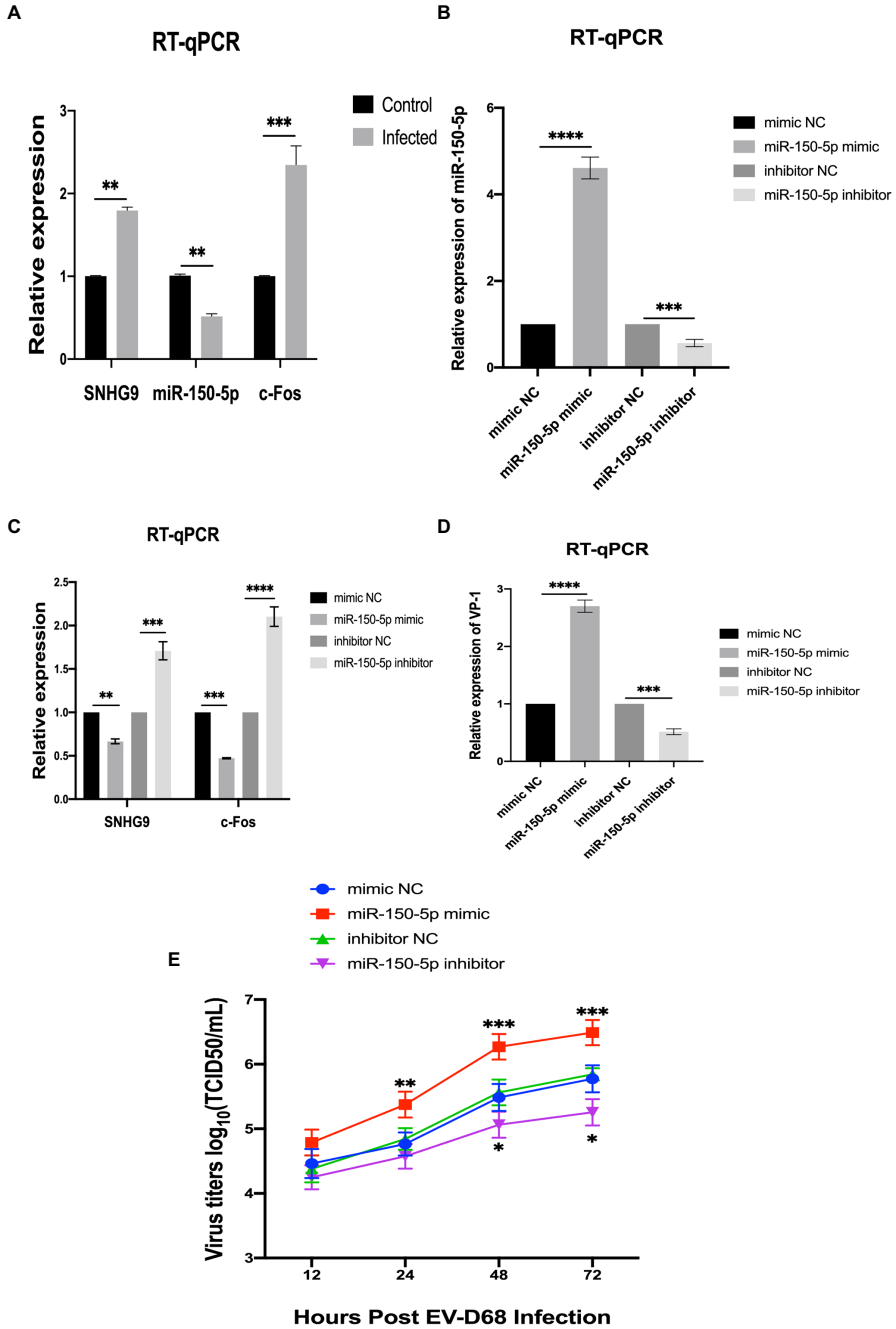
LncRNA SNHG9/miR-150-5p/c-Fos axis validation **(A)** qRT-PCR was utilized to determine the expression of SNHG9, miR-150-5p, and c-Fos in RD cells. RD cells were transfected with miR-150-5p mimic and inhibitor and then infected with EV-D68 for 24 h. **(B–D)** qRT-PCR for validation of RNA levels of SNHG9, miR-150-5p, VP1, and c-Fos in RD cells (^*^p < 0.05, ^**^p < 0.01, ^***^p < 0.001, ^****^p < 0.0001). **(E)** Viral titer assay for verification of viral replication levels. The data are expressed as mean standard deviation (*n* = 3) ^*^p < 0.05, ^**^p < 0.01, ^***^p < 0.001 in comparison to the mimic NC and inhibitor NC groups.

In a preliminary study in our laboratory, c-Fos was demonstrated to have an antiviral effect on RD cells infected with EV-D68 (Si et al., 2021). Overexpression of c-Fos in EV-D68 infection resulted in a significant decrease in viral VP1 expression at both the RNA and protein levels, according to this study. Viral titer experiments also revealed that c-Fos overexpression reduced viral replication capacity. Based on the ceRNA hypothesis, we reasonably hypothesized that lncRNA SNHG9 competes as a ceRNA for c-Fos to bind miR-150-5p, resulting in reduced degradation of c-Fos and thus indirectly regulating the expression level of c-Fos to regulate viral replication. Using qRT-PCR, the expression levels of SNHG9, miR-150-5p, and c-Fos were evaluated in RD cells infected with EV-68. Figure 6A reveals that EV-D68 infection of RD cells increased the expression of SNHG9 and c-Fos, whereas it decreased the expression of miR-150-5p. Therefore, when the miR-150-5p expression is down-regulated in this infection paradigm, SNHG9 and c-Fos expression will exhibit the inverse trend. In addition, considering the reliability of lncRNA SNHG9/miR-150-5p/c-Fos axis regulation, miR-150-5p mimic and inhibitor were transfected into RD cells by miRNA transfection technique. As shown in Figure 6B, qRT-PCR results demonstrated miR-150-5p expression levels. The RNA expression of lncRNA SNHG9 and c-Fos were further examined and the results in Figure 6C showed that when miR-150-5p was overexpressed, the expression of SNHG9 and c-Fos was significantly reduced compared with the control group. In contrast, SNHG9 and c-Fos expression increased when miR-150-5p was knocked down. It is reasonable to speculate whether these phenomena influence EV-D68 replication. Figures 6D,E shows that after EV-D68 infected RD cells with MOI = 1 for 24 h, the expression level of EV-D68 VP1 RNA was significantly increased in the miR-150-5p mimic group compared to the control group. Viral titer assays similarly showed a significant increase when miR-150-5p was overexpressed. This indicates that EV-D68 virus infectivity was enhanced, and replication ability was promoted at this time. When miR-150-5p was knocked down, the opposite change was verified by qRT-PCR and virus titer experiments. The above results suggest that miR-150-5p can regulate the replication of EV-D68 by affecting the expression of c-Fos, i.e., the antiviral effect of Fos.

### 3.7. SNHG9 is involved in EV-D68 replication regulation

**Figure 7:**
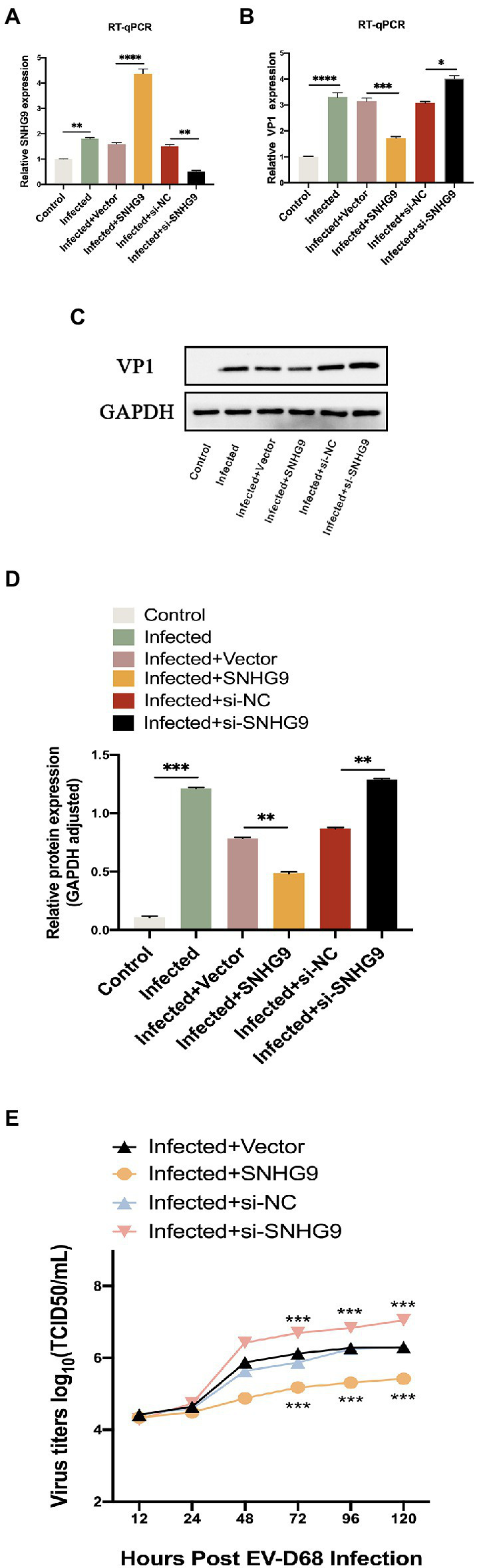
Effect of SNHG9 on viral replication in EV-D68-infected RD cells. RD cells were transfected with SNHG9-overexpression plasmid or si-SNHG9 and then infected with EV-D68 for 12 h. **(A,B)** Expression of SNHG9 and VP1 in RD cells was detected by qRT-PCR (^*^p < 0.05, ^**^p < 0.01, ^***^p < 0.001, ^****^p < 0.0001). **(C,D)** Western blot was utilized to evaluate viral VP1 protein levels. The data are expressed as mean ± SD (*n* = 3) ^*^p < 0.05, ^**^p < 0.01, and ^***^p < 0.001 vs. the control, vector, and si-NC groups. **(E)** For the replication levels of viral infections, viral titers were estimated. The data are expressed as mean standard deviation (n = 3) ^*^p < 0.05, ^**^p < 0.01, ^***^p < 0.001 in comparison to the vector and si-NC groups.

SNHG9 plasmid-mediated overexpression or siRNA-mediated knockdown was then utilized to confirm the involvement of SNHG9 in RD cells infected with EV-D68. Like Figure 7A, EV-D68 was infected with RD cells for 24 h at MOI = 1, and SNHG9 expression levels were validated by qRT-PCR. As shown in Figures 7B–E, when SNHG9 is overexpressed, TCID_50_/mL decreases significantly, western blot and qRT-PCR results similarly show a decrease in viral VP1 expression. The elimination of SNHG9 has a profound effect on these variables. These data imply that SNHG9 is involved in the regulation of EV-D68 infection propagation.

### 3.8. SNHG9 regulates viral replication during EV-D68 infection of RD cells *via* the miR-150-5p/c-Fos axis

**Figure 8:**
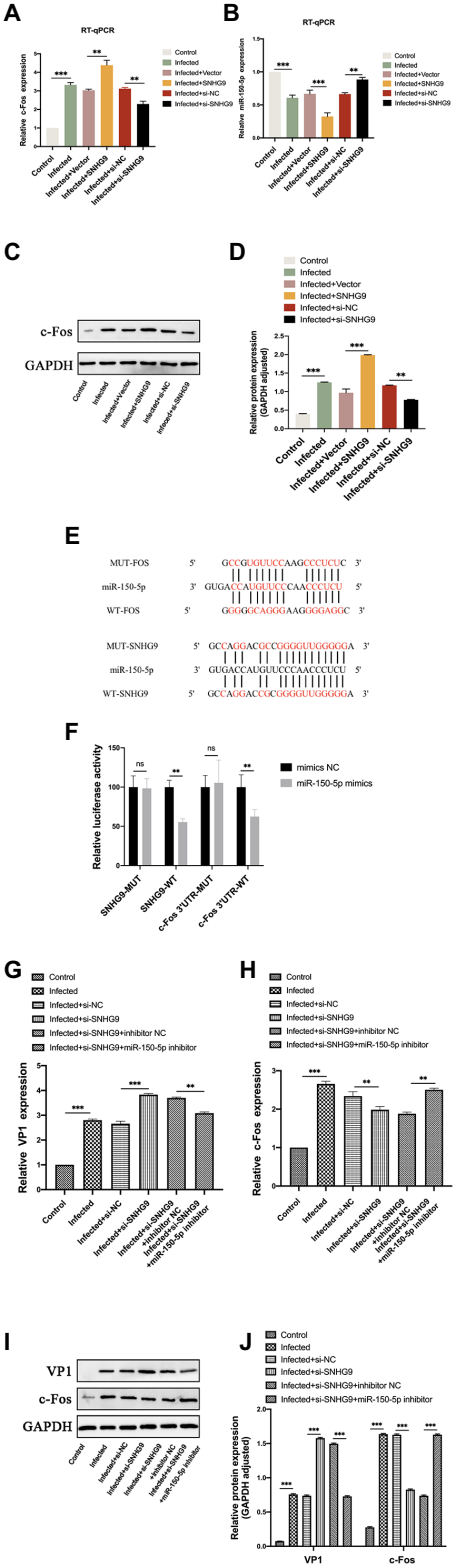
SNHG9 regulates viral replication during EV-D68 infection of RD cells *via* the miR-150-5p/c-Fos axis. RD cells were transfected with SNHG9-overexpression plasmid or si-SNHG9 and then infected with EV-D68 for 12 h. **(A,B)** The expression of miR-150-5p and c-Fos in RD cells was detected by qRT-PCR (^**^p < 0.01, ^***^p < 0.001). **(C,D)** Western blot was utilized to evaluate viral c-Fos protein levels. The data are expressed as mean ± SD (*n* = 3) ^*^p < 0.05, ^**^p < 0.01, and ^***^p < 0.001 vs. the control, vector, and si-NC groups. **(E)** The binding sites for miR-150-5p in the SNHG9/c-Fos sequence were shown. **(F)** The interaction between miR-150-5p and SNHG9/c-Fos was verified by a dual luciferase reporter assay. RD cells were transfected with si-SNHG9 or conjugated with miR-150-5p inhibitor, and then infected with EV-D68 for 12 h. **(G,H)** Detection of viral VP1 and c-Fos expression in RD cells by qRT-PCR. **(I,J)** The protein levels of c-Fos and VP1 in RD cells were evaluated by western blot assay. Data are expressed as mean ± SD (*n* = 3). ^*^p < 0.05, ^**^p < 0.01, ^***^p < 0.001 vs. the indicated group.

To further investigate the mechanism by which SNHG9 regulates EV-D68 replication, the miR-150-5p/c-Fos axis was focused on. As demonstrated by qRT-PCR and western blot in Figures 8A–D, upon EV-D68 infection of RD cells, miR-150-5p expression was decreased when SNHG9 was overexpressed and increased when SNHG9 was knocked down. In contrast, c-Fos expression was increased in RD cells when SNHG9 was overexpressed and decreased when SNHG9 was silenced. The connection between miR-150-5p and SNHG9/c-Fos in RD cells was then studied. Figure 8E depicts miR-150-5p binding sites to SNHG9 and c-Fos. Figure 8F demonstrates, using a dual luciferase reporter, that miR-150-5p mimics dramatically reduced the relative luciferase activity of the WT-SNHG9/c-Fos group. It did not affect the MUT-SNHG9/c-Fos group. Thus, miR-150-5p was capable of binding SNHG9/c-Fos directly in RD cells. As demonstrated in Figure 8G, the miR-150-5p inhibitor greatly decreased the stimulation of viral replication in EV-D68-infected RD cells when SNHG9 was knocked down, and this impact was confirmed at the protein level (Figures 8I,J). In addition, miR-150-5p inhibitors were found to prevent SNHG9 knockdown-mediated c-Fos downregulation (Figures 8H–J). It was inferred that SNHG9 regulates viral replication through the miR-150-5p/c-Fos axis to affect EV-D68 infection of RD cells.

## 4. Discussion

In recent years, the advent of extremely accurate next-generation sequencing technology and effective computational prediction platforms has led to the identification of numerous lncRNAs across all kingdoms of life (St Laurent et al., 2015; Cao et al., 2018; Qian et al., 2019). LncRNAs have emerged as critical regulators of gene expression. The idea that lncRNAs are the result of transcriptional noise and lack function has been dispelled. There is accumulating evidence that they play a crucial role in controlling major elements of growth, development, and stress adaption, making them one of the central players in gene expression regulation (Zhang et al., 2019; Rashidmayvan et al., 2022). Numerous studies have emphasized the relevance of lncRNAs in animal-virus interactions and their dual function as viral inducers and antiviral agents (Liu et al., 2019; Shen et al., 2020). LncRNA-ACOD1 is an example of a proviral factor that is induced by multiple viruses in humans and animals, including vesicular stomatitis virus (VSV), herpes simplex virus type 1 (HSV1), vaccinia virus (VACV), and influenza A/PR/8/34 virus (Runtsch and O’Neill, 2018). This lncRNA is increased in the absence of interferon (IFN), and its increased expression facilitates viral replication (Wang et al., 2017). LncRNA-MEG3 inhibits the replication of the respiratory syncytial virus (RSV). Its ectopic expression in RSV-infected BEAS-2B cells suppressed RSV infection *via* TLR4-dependent suppression of p38 NF-B and MAPK activation (Tao et al., 2018).

However, existing papers on EV-D68 infection do not contain a complete examination of lncRNA sequencing results. In our investigation, we obtained differentially expressed lncRNAs by performing high-throughput sequencing on RD cells with and without EV-D68 infection. A total of 375 differentially expressed lncRNAs were identified. Multiple targeting regulatory network maps were created based on the multiple mechanisms of action of lncRNAs to further investigate the regulatory mechanisms of lncRNAs in EV-D68 infection. In addition, it is more important to consider whether these different lncRNAs are important players in the regulation of genes in EV-D68 infection models.

To confirm our hypothesis, we validated the accuracy of lncRNA sequencing data using qRT-PCR and began gene regulation investigations using these differentially expressed significant lncRNAs. SNHG9 is a lncRNA whose coding gene is located on chromosome 17 and has a total length of approximately 416 base pairs. It was found to be associated with the development and progression of various cancers, including pancreatic cancer, glioblastoma, lung cancer, and ovarian cancer (Zhang et al., 2018; Lin et al., 2020; Chen et al., 2021; Wang et al., 2022). To date, there are limited investigations on SNHG9 in EV-D68 infection. Increasing data suggest that dysregulated miRNAs relate to viral infections. MiR-150’s role in HTLV-1 infection and T cell transformation, as well as TAT3/miR-125b-5p-1/HK2 signaling, are crucial for SeMet’s anti-PDCoV replication function (D’Agostino et al., 2022). However, the role of miR-150-5p in the course of EV-D68 infection remains unknown and needs to be investigated. The c-Fos proto-oncogene was initially identified as the gene responsible for Finkel-Biskis-Jinkins murine sarcoma virus-induced bone cancers (Cruz-Mendoza et al., 2022). Fos, however, is a protein that can operate as either a transcription factor or a transrepressor. Their relative propensity for binding to diverse interaction complexes, for instance, contributes to the diversity of this activation (O’Donnell et al., 2012). Importantly, it has been demonstrated that c-Fos is a target gene for miR-150-5p (Wang et al., 2021), which was consistent with our previous findings (Si et al., 2021).

SNHG9 expression is elevated in RD cells infected with EV-D68, according to this investigation. Moreover, overexpression of SNHG9 decreases viral replication in RD cells, whereas silencing SNHG9 reverses these effects. We verified this conclusion regarding RNA level, protein expression, and viral titer. These results imply that SNHG9 plays a role in the replication of EV-D68-infected RD cells and may represent a potential therapeutic target.

It has been demonstrated that SNHG9 exerts its biological effects by binding directly to miRNAs. For instance, SNHG9 promotes carcinogenesis in hepatoblastoma by downregulating miR-23a-5p and upregulating Wnt3a (Feng et al., 2021). SNHG9, when increased in glioblastoma stem cells (GSCs), functions as a ceRNA for miR-326 and promotes the development of GSCs (Wang et al., 2022). According to our research, SNHG9 might bind to miR-150-5p and regulate its expression in RD cells. In addition, SNHG9 overexpression inhibits viral growth in RD cells, a result that can be partially reversed by miR-150-5p inhibitors.

Both SNHG9 and c-Fos were found to have miR-150-5p binding sites using bioinformatics databases. Moreover, a dual luciferase reporter confirmed this hypothesis. Therefore, it is hypothesized that SNHG9 acts as a ceRNA to regulate the expression of c-Fos *via* binding to miR-150-5p. According to our findings, c-Fos is a target of miR-150-5p in RD cells. Moreover, in the RD cell line infected with EV-D68, c-Fos expression is raised when SNHG9 is overexpressed and lowered when SNHG9 is silenced. By functioning as a sponge for miR-150-5p, SNHG9 protects c-Fos from repression, demonstrating a post-transcriptional regulatory role. Therefore, overexpression of SNHG9 promotes c-Fos expression through binding to miR-150-5p, limiting viral replication in RD cells infected with EV-D68. In upcoming investigations, the Biosign database will continue to be used to investigate the mechanisms of gene regulation in RD cells after infection by EV-D68.

To sum up, differential expression of lncRNAs in RD cells infected with EV-D68 was obtained using high-throughput sequencing. Based on biomimetic data, the regulation of lncRNA genes in RD cell lines infected with EV-D68 was investigated. By regulating the miR-150-5p/c-Fos axis, our study demonstrated that SNHG9 overexpression inhibits viral multiplication in EV-D68 infection. Our findings indicate that SNHG9 may be a suitable therapeutic target for illnesses against EV-D68 infection.

## Data availability statement

The datasets presented in this study can be found in online repositories. The names of the repository/repositories and accession number(s) can be found in the article/Supplementary material.

## Author contributions

HF, JS, and XT: conceptualization. HF: data curation, investigation, and writing—original draft. HF, XT, and HL: formal analysis. CY: funding acquisition, supervision, and validation. HF, XT, HL, and AL: methodology. HF, SG, and CY: project administration. NL, HL, and CY: resources. LX: software. HF, SG, and JS: visualization. LX and CY: writing—review and editing. All authors contributed to the article and approved the submitted version.

## Funding

This work was funded by Natural Science Foundation of Chongqing (cstc2016jcyjA0212), Natural Science Foundation of Chongqing (cstc2017jcyjAX0409), and Graduate Training Fund of Chongqing Medical University.

## Conflict of interest

The authors declare that the research was conducted in the absence of any commercial or financial relationships that could be construed as a potential conflict of interest.

## Publisher’s note

All claims expressed in this article are solely those of the authors and do not necessarily represent those of their affiliated organizations, or those of the publisher, the editors and the reviewers. Any product that may be evaluated in this article, or claim that may be made by its manufacturer, is not guaranteed or endorsed by the publisher.
