# Supplementary material for: Long non-coding RNA SNHG9 regulates viral replication in rhabdomyosarcoma cells infected with enterovirus D68 via miR-150-5p/c-Fos axis
Source: Front Microbiol. 2023 Jan 19;13:1081237. doi: 10.3389/fmicb.2022.1081237 (PMC9893417; doi:10.3389/fmicb.2022.1081237)
Supplement: Supplementary file 1 [file Data_Sheet_1.PDF]

Supplementary Material

Data Sheet 1 Differential lncRNAs database

Gene ID	Gene Symbol	Type	Lnc_C1 FPKM	Lnc_C2 FPKM	Lnc_C3 FPKM	Lnc_T1 FPKM	Lnc_T2 FPKM	Lnc_T3 FPKM
**100128531**	'LOC100128531'	lncRNA	1.8	2.7	2.27	0.85	1.7	0.86
**100128560**	'MYHAS'	lncRNA	0.81	0.86	0.55	0.15	0.52	0.34
**100129215**	'LOC100129215'	lncRNA	0.28	0.8	0.85	0	0.83	0.11
**100129550**	'LINC02035'	lncRNA	0.25	0.27	0.15	0.04	0	0
**100130155**	'MIR124-2HG'	lncRNA	0.24	0.28	0.23	0.14	0.11	0.13
**100134391**	'LOC100134391'	lncRNA	0.11	0.06	0.13	0.02	0	0.04
**100190986**	'LOC100190986'	lncRNA	0	6.14	0.41	15.63	0	8.23
**100288346**	'NCAM1-AS1'	lncRNA	0.15	0.15	0.13	0.05	0.08	0.07
**100303453**	'TSNAX-DISC1'	lncRNA	1.32	0.78	1.38	0.93	0.68	0.91
**100499177**	'THAP9-AS1'	lncRNA	1.12	1.13	0.84	4.72	3.15	3.7
**100505555**	'LOC100505555'	lncRNA	0.99	0.79	0.98	0.62	0.43	0.32
**100505768**	'LINC01364'	lncRNA	0.27	0.25	0.24	0.07	0.14	0.15
**100505875**	'LINC01088'	lncRNA	0.77	0.53	0.6	0.18	0.3	0.23
**100506071**	'LOC100506071'	lncRNA	1.04	1.11	0.47	0.39	0.35	0.29
**100507257**	'MEG9'	lncRNA	1.05	0.81	1.21	0.53	0.48	0.49
**100526836**	'BLOC1S5-TXNDC5'	lncRNA	5.11	4.44	6.73	2.89	3.81	2.9
**100526837**	'EEF1E1-BLOC1S5'	lncRNA	0	0	0	0.22	0	0.23
**100528007**	'BORCS7-ASMT'	lncRNA	1.04	1.3	1.22	0	0.23	1.25
**100529264**	'RAB4B-EGLN2'	lncRNA	0.08	0.09	0.08	0.32	0.2	0.45
**100529855**	'ZNF625-ZNF20'	lncRNA	0	0.59	0.22	1.93	1.47	1.09
**100532735**	'INO80B-WBP1'	lncRNA	0.22	0.25	0.21	1.04	1.78	0.93
**100533975**	'SLMO2-ATP5E'	lncRNA	3.13	3.29	3.4	11.07	12.47	9.39
**100652853**	'LINC01023'	lncRNA	0.6	0.88	1.16	0.11	0.2	0.08
**100861545**	'LINC00561'	lncRNA	0.43	0.03	0.08	0.01	0.05	0
**100874296**	'KCNH1-IT1'	lncRNA	0	0	0	0.28	0.9	0.51
**100996291**	'LINC01993'	lncRNA	0.3	0.29	0.25	0.04	0.11	0.13
**100996325**	'LOC100996325'	lncRNA	2.54	2.68	2.67	1.06	0.69	1.31
**100996335**	'FAM230H'	lncRNA	3.48	4.61	1.23	1.01	0.63	0.86
**101926907**	'LOC101926907'	lncRNA	0.29	0.74	0.51	0.05	0.05	0.2
**101927182**	'LOC101927182'	lncRNA	0.43	0.57	0.39	0.33	0.16	0.33

**101928059**	'LOC101928059'	lncRNA	0.99	0.85	0.55	1.65	1.99	1.49
**101928093**	'LOC101928093'	lncRNA	0	0.08	0	1.66	1.75	0.8
**101928243**	'DARS-AS1'	lncRNA	1.41	1.93	2.17	1.03	1.44	1.19
**101928324**	'LOC101928324'	lncRNA	0.86	0.75	0.49	0.09	0.17	0
**101928402**	'LOC101928402'	lncRNA	0.49	0.39	0.4	1.01	1.09	1.03
**101928813**	'LOC101928813'	lncRNA	0.19	0.16	0.12	0.49	0.41	0.99
**101929181**	'LINC01647'	lncRNA	0.39	0.57	0.36	0.81	1.05	1.87
**101929260**	'LINC01920'	lncRNA	1.49	1.29	1.38	0.54	0.72	0.74
**101929268**	'LOC101929268'	lncRNA	0.7	0.5	1.14	0.4	0.07	0.72
**101929511**	'LINC01970'	lncRNA	0.89	1.01	1.13	2.41	2.33	2.81
**101929547**	'LOC101929547'	lncRNA	1.15	0.97	0.43	0.24	0.38	0.62
**101929563**	'LOC101929563'	lncRNA	0.68	0.93	1.01	0.33	0.58	0.29
**101929719**	'LOC101929719'	lncRNA	0.25	0.14	0.15	0.06	0.03	0.03
**101929947**	'ITGA6-AS1'	lncRNA	1	0.43	0.58	0.3	0.16	0.77
**101930665**	'LOC101930665'	lncRNA	12.63	10.13	10.37	48.45	44.74	44.98
**102723167**	'MIR133A1HG'	lncRNA	1.18	0.91	0.55	0.44	0.27	0.3
**102723763**	'LOC102723763'	lncRNA	0.42	0.47	0.44	0.51	0.85	1.01
**102724637**	'LOC102724637'	lncRNA	0.83	0.69	0.45	0.06	0.17	0.23
**103352670**	'LINC01419'	lncRNA	0.05	0	0.04	0.32	0.32	0.68
**105369332**	'LOC105369332'	lncRNA	0.28	0.36	0.22	0.95	0.98	1.27
**105369381**	'LOC105369381'	lncRNA	0.44	0.21	0.08	0.06	0.01	0.5
**105369551**	'LOC105369551'	lncRNA	0.14	0.18	0.15	0.05	0.04	0.05
**105369720**	'LOC105369720'	lncRNA	0.2	0.3	0.17	0.09	0.06	0.09
**105369846**	'LOC105369846'	lncRNA	0.6	0.55	0.48	0.26	0.33	0.22
**105369890**	'LOC105369890'	lncRNA	11.88	12.67	10.73	5.29	5.44	6.04
**105370148**	'LOC105370148'	lncRNA	0.11	0.03	0.19	0.45	0.29	0.73
**105370384**	'LOC105370384'	lncRNA	0.91	1.17	0.71	3.21	3.51	1.76
**105370576**	'LOC105370576'	lncRNA	0.52	0.67	0.93	0.23	0.22	0.32
**105370765**	'LOC105370765'	lncRNA	0.38	0.5	0.34	0.09	0.08	0.09
**105370789**	'LOC105370789'	lncRNA	0.02	0.09	0	0.16	0.21	0.63
**105370884**	'LOC105370884'	lncRNA	0.33	0.57	0.75	0	0	0
**105371159**	'LOC105371159'	lncRNA	0.23	0.43	0.39	0.07	0.11	0.43
**105371291**	'LOC105371291'	lncRNA	0.38	0.24	0.4	0.1	0.08	0.06
**105371343**	'LOC105371343'	lncRNA	0.21	0.25	0.3	0.06	0.07	0.09
**105371382**	'LOC105371382'	lncRNA	0.12	0.19	0.1	0.08	0.07	0.04
**105371566**	'LOC105371566'	lncRNA	2.27	1.66	2.13	0.78	0.78	1.04
**105371620**	'LOC105371620'	lncRNA	0.1	0.12	0.05	0.43	0.32	0.26
**105371686**	'LOC105371686'	lncRNA	1.7	1.71	2.51	1.48	1.67	0.99

**105371760**	'LOC105371760'	lncRNA	0.17	0.18	0.06	1.07	0.7	0.55
**105372003**	'LOC105372003'	lncRNA	0.22	0.16	0.16	0.04	0.06	0.02
**105372436**	'LOC105372436'	lncRNA	12.96	18.76	20.73	72.41	61.34	82.73
**105372793**	'LOC105372793'	lncRNA	0.31	0.28	0.27	0.11	0.04	0.01
**105373148**	'LOC105373148'	lncRNA	0.32	0.27	0.84	0.25	0.06	0.02
**105373366**	'IDS2'	lncRNA	0.74	0.82	1.17	0.33	0.44	0.25
**105373715**	'LOC105373715'	lncRNA	1.08	1.28	1.67	0.69	0.46	0.56
**105373775**	'LOC105373775'	lncRNA	2.59	2.35	1.49	0.86	0.66	0.65
**105374029**	'LOC105374029'	lncRNA	3.8	3.81	3.88	2.07	2.24	1.78
**105374117**	'LOC105374117'	lncRNA	0.03	0.12	0.09	0.29	0.26	0.8
**105374118**	'LOC105374118'	lncRNA	0.01	0	0	0.06	0.06	0.1
**105374129**	'LOC105374129'	lncRNA	0.11	0.05	0.11	0.01	0.01	0
**105374140**	'LOC105374140'	lncRNA	0.11	0.15	0.08	0.03	0.08	0.04
**105374439**	'LOC105374439'	lncRNA	0.16	0.26	0.72	0.06	0.13	0.05
**105374464**	'LOC105374464'	lncRNA	4.23	3.51	3.5	2.27	1.13	1.44
**105374538**	'LOC105374538'	lncRNA	0.1	0.08	0.14	0.02	0	0.02
**105374676**	'LINC02241'	lncRNA	1.06	1.52	0.79	0.69	0.28	1.84
**105374798**	'LOC105374798'	lncRNA	0.56	0.48	0.56	0.01	0.38	0.68
**105374985**	'LOC105374985'	lncRNA	0.37	0.32	0.71	1.2	0.79	1.25
**105374986**	'LOC105374986'	lncRNA	0.04	0.06	0.05	1.89	1.27	1.35
**105374988**	'LOC105374988'	lncRNA	0.13	0.05	0.09	0.6	0.42	0.64
**105374989**	'LOC105374989'	lncRNA	0.05	0.09	0.03	0.61	0.61	0.46
**105374995**	'LOC105374995'	lncRNA	0.26	0.18	0.29	4.27	4.3	4.32
**105375019**	'LOC105375019'	lncRNA	0.13	0.2	0.19	0.46	0.55	0.37
**105375218**	'LOC105375218'	lncRNA	1.73	1.32	1.46	0.52	0.6	0.71
**105375544**	'LOC105375544'	lncRNA	0.22	0.53	0.2	0.05	0.04	0.17
**105375586**	'LOC105375586'	lncRNA	1.76	1.76	2.02	1.08	1.17	1.01
**105375655**	'LOC105375655'	lncRNA	0.7	0.68	0.43	0.29	0.19	0.22
**105375730**	'LOC105375730'	lncRNA	0.19	0.09	0.18	0.05	0.06	0.03
**105375731**	'LOC105375731'	lncRNA	0.77	0.57	0.65	0.19	0.1	0.3
**105375740**	'LOC105375740'	lncRNA	0.4	0.56	0.68	1.87	1.39	1.53
**105376219**	'LOC105376219'	lncRNA	2.47	2.16	0.81	0.35	0.62	0.58
**105376301**	'LOC105376301'	lncRNA	0.13	0.1	0.1	0.32	0.45	0.91
**105376323**	'LOC105376323'	lncRNA	1.18	1.05	0.93	0.32	0.62	0.45
**105376987**	'LOC105376987'	lncRNA	0.45	0.94	0.89	0.2	0.02	0.26
**105377295**	'LOC105377295'	lncRNA	0.43	0.88	1.13	0.39	0.1	0.33
**105377319**	'LOC105377319'	lncRNA	0.11	0.08	0.17	0.05	0.03	0.04
**105377451**	'LOC105377451'	lncRNA	2.62	2.12	2.55	0.55	1.52	0.79
**105377477**	'LINC02507'	lncRNA	0.08	0.22	0.2	0.04	0.05	0.05

**105378046**	'LOC105378046'	lncRNA	0.23	0.28	0.3	0.1	0.09	0.17
**105378171**	'LOC105378171'	lncRNA	10.16	8	0.7	1	0.87	1.06
**105378198**	'LOC105378198'	lncRNA	0	0.2	0.09	0.8	1.58	1.04
**105378255**	'LOC105378255'	lncRNA	0.06	0.08	0.08	0.33	0.44	0.14
**105378558**	'LOC105378558'	lncRNA	0.18	0.15	0.41	0.06	0.04	0.15
**105379199**	'LOC105379199'	lncRNA	1.81	1.23	1.2	0.49	0.68	0.44
**105379227**	'LOC105379227'	lncRNA	0.02	0.09	0.06	0.23	0.05	0.29
**105379255**	'LOC105379255'	lncRNA	0.33	0.22	0.19	0.81	0.62	0.6
**105379548**	'LOC105379548'	lncRNA	1.38	0.82	1.53	0.22	0.55	0.72
**105379551**	'LOC105379551'	lncRNA	1.68	1.67	1.75	0.81	0.64	0.91
**107983979**	'LOC107983979'	lncRNA	0.86	0.77	0.91	2.8	2.02	3.29
**107984210**	'LOC107984210'	lncRNA	0.45	0.44	0.53	0.21	0.37	0.31
**107984301**	'LOC107984301'	lncRNA	1.27	1.43	0.69	0.37	0.33	0.73
**107984366**	'LOC107984366'	lncRNA	6.84	6.36	6.96	3.13	3.1	3.23
**107984591**	'LOC107984591'	lncRNA	1.05	0.99	1.08	0.4	0.6	0.53
**107984661**	'LOC107984661'	lncRNA	1.11	1.68	1.8	13.82	14.41	26.06
**107984713**	'LOC107984713'	lncRNA	1.02	1.42	1	0.51	0.42	0.59
**107984718**	'LOC107984718'	lncRNA	0.49	0.56	0.57	0.22	0.23	0.19
**107984773**	'LINC01852'	lncRNA	1.97	2.05	1.69	0.82	0.96	0.85
**107984988**	'LOC107984988'	lncRNA	0.1	0.11	0.22	0.48	0.27	0.47
**107985172**	'LOC107985172'	lncRNA	3.3	4.56	4.64	1.52	2.08	2.36
**107985207**	'LOC107985207'	lncRNA	0.87	0.45	0.99	2.69	1.69	2.33
**107985430**	'LOC107985430'	lncRNA	0.87	0.83	1.06	0.43	0.34	0.57
**107985699**	'LOC107985699'	lncRNA	0.19	0.17	0.47	0.11	0.08	0.06
**107985936**	'LOC107985936'	lncRNA	0.46	0.51	0.57	0.13	0.27	0.19
**107985977**	'LOC107985977'	lncRNA	0.76	0.96	0.93	0.4	0.35	0.4
**107985998**	'LOC107985998'	lncRNA	0.26	0.48	0.82	0.14	0.28	0.24
**107986196**	'LOC107986196'	lncRNA	0.78	0.83	0.48	2.16	1.49	1.64
**107986593**	'LOC107986593'	lncRNA	0.18	0.25	0.14	0.35	0.42	0.44
**107986632**	'LOC107986632'	lncRNA	0.32	0.45	0.49	0.12	0.25	0.22
**107986674**	'LOC107986674'	lncRNA	0.2	0.3	0.32	0.07	0.11	0.11
**107986717**	'LOC107986717'	lncRNA	0.86	0.79	0.57	0.13	0.4	0.35
**107986874**	'LOC107986874'	lncRNA	6.49	6.21	6.3	2.47	2.7	2.78
**107987127**	'LOC107987127'	lncRNA	0.77	1.09	1	2.13	3.47	2.05
**107987128**	'LOC107987128'	lncRNA	1.62	1.55	1.74	0.82	0.87	0.76
**107987208**	'LOC107987208'	lncRNA	0.61	1.22	1.41	0.12	0.46	1.28
**107987293**	'LOC107987293'	lncRNA	29.53	36.14	36.75	63.04	53.66	76.12
**107987392**	'LOC107987392'	lncRNA	1.09	0.22	0.23	0	0.43	0

**107987448**	'LOC107987448'	lncRNA	0.4	0.6	0.02	1.83	0.41	0.07
**109136579**	'TALAM1'	lncRNA	0.54	0.87	0.45	2.43	2.17	2.1
**110091768**	'LOC110091768'	lncRNA	0.6	0.61	0.77	0.23	0.33	0.39
**112267938**	'LOC112267938'	lncRNA	0.75	0.74	0.57	0.2	0.43	0.31
**112268061**	'LOC112268061'	lncRNA	0.67	0.82	0.86	0.35	0.42	0.38
**112268093**	'LOC112268093'	lncRNA	0.26	0.3	0.29	0.14	0.07	0.1
**112268172**	'LOC112268172'	lncRNA	0	0	0.11	0.37	0.34	0
**112268190**	'LOC112268190'	lncRNA	1.17	0.91	0.5	1.95	2.41	2.26
**112268230**	'LOC112268230'	lncRNA	0	0.1	0.06	0.02	0	0
**112268327**	'LOC112268327'	lncRNA	1.33	1.18	1.31	0.32	0.97	0.61
**145624**	'PWAR1'	lncRNA	0.88	0.8	0.76	0.22	0.21	0.2
**149134**	'LINC01341'	lncRNA	1.89	1.51	1.2	0.85	0.8	0.88
**220980**	'TMEM72-AS1'	lncRNA	1.28	1.24	1.4	0.38	0.55	0.59
**27004**	'TCL6'	lncRNA	3.96	2.79	3.53	6.81	7.36	10.75
**284424**	'MIR7-3HG'	lncRNA	0.02	0.08	0.07	0.26	0.29	0.35
**29931**	'LINC00312'	lncRNA	0.89	1.21	1.26	0.57	0.53	0.44
**3653**	'IPW'	lncRNA	2.81	2.68	2.35	0.85	0.9	0.88
**387644**	'FAM238C'	lncRNA	0.47	0.66	0.52	0.15	0.38	0.39
**400804**	'C1orf140'	lncRNA	1.17	0.95	1.38	0.58	0.74	0.43
**400932**	'LINC00898'	lncRNA	0.36	0.39	0.38	0.12	0.25	0.2
**401237**	'CASC15'	lncRNA	0.34	0.4	0.42	0.15	0.26	0.16
**401261**	'LOC401261'	lncRNA	2.1	1.85	0	0	1.62	0
**401471**	'LOC401471'	lncRNA	0.06	0.09	0.07	0.03	0.03	0.01
**439936**	'C5orf17'	lncRNA	0.7	0.91	0.73	0.17	0.03	0.44
**574406**	'ADAMTSL4-AS1'	lncRNA	3	2	2.38	6.32	5.12	4.45
**594842**	'HAS2-AS1'	lncRNA	0.82	0.82	0.92	0.4	0.51	0.29
**64493**	'LINC00235'	lncRNA	0	0	0	1.89	0.86	0.92
**653160**	'LOC653160'	lncRNA	0.76	0.74	0.9	0.34	0.4	0.24
**728431**	'LINC01137'	lncRNA	0.66	0.66	0.64	0.34	0.26	0.16
**735301**	'SNHG9'	lncRNA	4.23	1.35	2.3	6.3	5.38	6.31
**79104**	'MEG8'	lncRNA	34.87	40.75	39.51	15.82	17.17	20.4
**79999**	'LOC79999'	lncRNA	112.35	137.1	146.05	469.05	444.02	351.56
**81571**	'MIR600HG'	lncRNA	1.01	1.36	0.66	0.5	0.6	0
**81698**	'LINC00597'	lncRNA	0.71	0.94	0.73	0.29	0.38	0.3
**84981**	'MIR22HG'	lncRNA	2.65	2.84	2.58	1.24	0.54	0.81
**91450**	'LOC91450'	lncRNA	0.95	0.96	0.85	0.23	0.26	0.29
**BGIG9606_36237**	'BGIG9606_36237'	lncRNA	0.26	0.11	0.17	0.74	0.53	1.22
**BGIG9606_36305**	'BGIG9606_36305'	lncRNA	0.26	0.34	0.32	0.14	0.08	0.21
**BGIG9606_36582**	'BGIG9606_36582'	lncRNA	0	0	0.02	0.51	0.7	1.11

**BGIG9606_36680**	'BGIG9606_36680'	lncRNA	0.43	0.32	0.45	0.13	0.12	0.12
**BGIG9606_36740**	'BGIG9606_36740'	lncRNA	0.02	0.08	0.07	0.22	0.24	0.3
**BGIG9606_36877**	'BGIG9606_36877'	lncRNA	0.05	0.03	0.08	0.77	0.72	0.78
**BGIG9606_36972**	'BGIG9606_36972'	lncRNA	0.2	0.21	0	0.76	1.98	1.61
**BGIG9606_37304**	'BGIG9606_37304'	lncRNA	0.39	0.45	0.38	1.23	1.05	0.67
**BGIG9606_37305**	'BGIG9606_37305'	lncRNA	0.52	0.31	0.31	0.72	0.82	0.9
**BGIG9606_37508**	'BGIG9606_37508'	lncRNA	0.05	0.05	0.07	0.02	0.03	0.01
**BGIG9606_37576**	'BGIG9606_37576'	lncRNA	0.19	0.23	0.06	0.41	0.47	0.19
**BGIG9606_37613**	'BGIG9606_37613'	lncRNA	0.98	0.69	0.7	0.91	2.32	1.45
**BGIG9606_37677**	'BGIG9606_37677'	lncRNA	0.03	0	0	0.11	0.05	0.09
**BGIG9606_38525**	'BGIG9606_38525'	lncRNA	1.35	1.72	2.7	0.3	1.15	0.51
**BGIG9606_38756**	'BGIG9606_38756'	lncRNA	0.29	0.11	0.14	2.31	2.18	3.19
**BGIG9606_39245**	'BGIG9606_39245'	lncRNA	0.27	0.44	0.26	0.9	0.66	0.8
**BGIG9606_39319**	'BGIG9606_39319'	lncRNA	0.17	0.16	0.2	0.09	0.11	0.08
**BGIG9606_39344**	'BGIG9606_39344'	lncRNA	0.78	0.76	0.69	0	0	0.23
**BGIG9606_39429**	'BGIG9606_39429'	lncRNA	0.17	0.34	0.26	0.12	0	0.15
**BGIG9606_39612**	'BGIG9606_39612'	lncRNA	1.72	0.99	1.31	3.62	3.28	3.06
**BGIG9606_39671**	'BGIG9606_39671'	lncRNA	2.63	2.68	2.69	1.13	1.36	1.34
**BGIG9606_39771**	'BGIG9606_39771'	lncRNA	0.13	0.14	0.17	0.74	0.65	0.32
**BGIG9606_39893**	'BGIG9606_39893'	lncRNA	0.04	0	0.02	0.3	0.21	0.28
**BGIG9606_39916**	'BGIG9606_39916'	lncRNA	0.42	0.37	0.48	0.13	0.14	0.21
**BGIG9606_39965**	'BGIG9606_39965'	lncRNA	0.03	0.03	0.05	0.01	0.01	0.01
**BGIG9606_40019**	'BGIG9606_40019'	lncRNA	0.12	0.04	0.09	0.34	0.2	0.25
**BGIG9606_40089**	'BGIG9606_40089'	lncRNA	0.08	0.09	0.07	0.26	0.26	0.16
**BGIG9606_40372**	'BGIG9606_40372'	lncRNA	0.36	0.24	0.28	0.02	0.09	0.08
**BGIG9606_40400**	'BGIG9606_40400'	lncRNA	0.13	0.1	0.15	0.46	0.84	1.05
**BGIG9606_40559**	'BGIG9606_40559'	lncRNA	1.18	2.62	2.77	9.81	9.71	9.8
**BGIG9606_40681**	'BGIG9606_40681'	lncRNA	0.77	0.79	0.89	0.32	0.23	0.16
**BGIG9606_41215**	'BGIG9606_41215'	lncRNA	0.29	0.19	0.28	0.52	0.75	0.79
**BGIG9606_41351**	'BGIG9606_41351'	lncRNA	0.23	0.16	0.23	14.62	13.89	11.14
**BGIG9606_41359**	'BGIG9606_41359'	lncRNA	0.18	0.36	0.19	0.1	0.08	0.16
**BGIG9606_41482**	'BGIG9606_41482'	lncRNA	0.57	1.06	0.7	0.27	0.44	0.32
**BGIG9606_41526**	'BGIG9606_41526'	lncRNA	0.68	0.58	0.62	0.27	0.36	0.24
**BGIG9606_41572**	'BGIG9606_41572'	lncRNA	0.7	0.43	1.26	2.17	2.48	4.54
**BGIG9606_41648**	'BGIG9606_41648'	lncRNA	0.07	0	0	0.18	0.34	0.76
**BGIG9606_42117**	'BGIG9606_42117'	lncRNA	3.39	3.38	5.71	10.08	11.12	14.06
**BGIG9606_42126**	'BGIG9606_42126'	lncRNA	0	0	0.09	1.1	1.14	1.86
**BGIG9606_42128**	'BGIG9606_42128'	lncRNA	0	0	0	1.33	1.12	1.38

**BGIG9606_42205**	'BGIG9606_42205'	lncRNA	0	0	0	0.11	0.11	0.07
**BGIG9606_42447**	'BGIG9606_42447'	lncRNA	0	0.24	0	0.32	0.1	0.48
**BGIG9606_42682**	'BGIG9606_42682'	lncRNA	0	0.1	0.28	2.23	0.94	0.93
**BGIG9606_42685**	'BGIG9606_42685'	lncRNA	0.1	0.21	0	4.25	2.76	3.58
**BGIG9606_42688**	'BGIG9606_42688'	lncRNA	0	0	0.07	0.42	0.27	0.16
**BGIG9606_42693**	'BGIG9606_42693'	lncRNA	0.06	0.12	0.06	1.29	1.01	0.99
**BGIG9606_42694**	'BGIG9606_42694'	lncRNA	0	0	0	0.72	1.02	0.33
**BGIG9606_42695**	'BGIG9606_42695'	lncRNA	0.35	0.09	0.17	2.13	1.91	2.82
**BGIG9606_42811**	'BGIG9606_42811'	lncRNA	2.59	2.49	2.08	1.06	0.9	1.22
**BGIG9606_43256**	'BGIG9606_43256'	lncRNA	0.01	0.01	0	0.08	0.09	0.18
**BGIG9606_43258**	'BGIG9606_43258'	lncRNA	0	0.75	0.18	1.15	1.74	1.94
**BGIG9606_43884**	'BGIG9606_43884'	lncRNA	0.69	0.71	0.87	0.11	0.26	0.46
**BGIG9606_43889**	'BGIG9606_43889'	lncRNA	0.35	0.36	0.28	1.29	1.55	1
**BGIG9606_44056**	'BGIG9606_44056'	lncRNA	1.34	1.83	1.47	10.39	7.53	6.98
**BGIG9606_44431**	'BGIG9606_44431'	lncRNA	0.01	0	0.01	0.2	0.19	0.13
**BGIG9606_44552**	'BGIG9606_44552'	lncRNA	0.52	0.5	0.46	0.27	0.19	0.21
**BGIG9606_44585**	'BGIG9606_44585'	lncRNA	0.56	0.85	0.86	0.21	0.32	0.45
**BGIG9606_44769**	'BGIG9606_44769'	lncRNA	0.91	0.84	1.37	5.57	4.79	5.92
**BGIG9606_44836**	'BGIG9606_44836'	lncRNA	0.42	1.09	0.79	0	0	0.46
**BGIG9606_45311**	'BGIG9606_45311'	lncRNA	0.99	0.6	0.69	0.04	0.33	0.57
**BGIG9606_45435**	'BGIG9606_45435'	lncRNA	0.02	0	0	0.09	0	0.27
**BGIG9606_45455**	'BGIG9606_45455'	lncRNA	0.15	0.2	0.26	1	1.01	0.79
**BGIG9606_45642**	'BGIG9606_45642'	lncRNA	0.18	0.29	0.14	0	0.03	0.06
**BGIG9606_45979**	'BGIG9606_45979'	lncRNA	1.56	1.51	1.77	1.49	0.84	0.6
**BGIG9606_45981**	'BGIG9606_45981'	lncRNA	1.64	2.22	2.2	0.7	0.67	1.07
**BGIG9606_46242**	'BGIG9606_46242'	lncRNA	0.13	0.11	0.12	0.47	0.4	0.28
**BGIG9606_46389**	'BGIG9606_46389'	lncRNA	0.18	0.33	0.22	0.86	0.71	0.7
**BGIG9606_46392**	'BGIG9606_46392'	lncRNA	0.3	0.2	0.22	1.08	1.49	1.47
**BGIG9606_46437**	'BGIG9606_46437'	lncRNA	0.34	0.35	0.37	0.53	0.69	0.9
**BGIG9606_46524**	'BGIG9606_46524'	lncRNA	0.4	0.48	0.41	0.04	0.07	0.17
**BGIG9606_46575**	'BGIG9606_46575'	lncRNA	0.05	0.25	0.14	0.02	0	0.03
**BGIG9606_46646**	'BGIG9606_46646'	lncRNA	0.42	0.45	0.62	0.07	0.24	0.16
**BGIG9606_46803**	'BGIG9606_46803'	lncRNA	0	0	0	1.34	1.42	0.7
**BGIG9606_46995**	'BGIG9606_46995'	lncRNA	0.47	0.31	0.55	6.28	5.8	6.4
**BGIG9606_47021**	'BGIG9606_47021'	lncRNA	0.21	0.12	0.16	0.02	0.06	0
**BGIG9606_47101**	'BGIG9606_47101'	lncRNA	0.13	0.2	0.15	0.08	0.05	0.08
**BGIG9606_47301**	'BGIG9606_47301'	lncRNA	1	0.77	0.78	0.08	0.23	0.38
**BGIG9606_47303**	'BGIG9606_47303'	lncRNA	0.46	0.54	0.5	0.13	0.16	0.04
**BGIG9606_47402**	'BGIG9606_47402'	lncRNA	0.66	0.45	0.61	0.2	0.25	0.32

**BGIG9606_47847**	'BGIG9606_47847'	lncRNA	0	0	0.15	0.39	0.28	1.1
**BGIG9606_48323**	'BGIG9606_48323'	lncRNA	0.84	0.76	0.4	0.23	0.38	0.29
**BGIG9606_48339**	'BGIG9606_48339'	lncRNA	0.48	0.9	1.11	2.45	1.98	2.06
**BGIG9606_48340**	'BGIG9606_48340'	lncRNA	3.41	5.53	5.05	10.79	8.9	10.08
**BGIG9606_48452**	'BGIG9606_48452'	lncRNA	0.33	0.36	0.31	0.11	0.1	0.14
**BGIG9606_48557**	'BGIG9606_48557'	lncRNA	0.47	0.51	0.6	1.47	1.65	1.07
**BGIG9606_48624**	'BGIG9606_48624'	lncRNA	5.66	5.93	5.3	3.26	3.09	1.95
**BGIG9606_48744**	'BGIG9606_48744'	lncRNA	0.17	0.12	0.38	0.87	0.74	1.15
**BGIG9606_48878**	'BGIG9606_48878'	lncRNA	0.22	0.23	0.86	8.46	3.25	8.22
**BGIG9606_49002**	'BGIG9606_49002'	lncRNA	0.25	0.15	0.27	0.1	0.06	0.09
**BGIG9606_49041**	'BGIG9606_49041'	lncRNA	0.65	0.48	0.8	3.78	3.68	3.74
**BGIG9606_49077**	'BGIG9606_49077'	lncRNA	0.07	0.13	0.12	0.47	0.44	0.35
**BGIG9606_49400**	'BGIG9606_49400'	lncRNA	0.53	0.59	0.58	0.29	0.27	0.38
**BGIG9606_49431**	'BGIG9606_49431'	lncRNA	0.67	0.7	0.26	2.05	1.14	2.08
**BGIG9606_49502**	'BGIG9606_49502'	lncRNA	28.01	17.11	22.25	0.45	14.7	8.53
**BGIG9606_49708**	'BGIG9606_49708'	lncRNA	0.64	0.08	0.3	0.62	0.81	0.82
**BGIG9606_49781**	'BGIG9606_49781'	lncRNA	1.48	1.73	1.16	0.24	0.79	0.33
**BGIG9606_50037**	'BGIG9606_50037'	lncRNA	4.46	3.1	3.15	1.3	1.14	1.24
**BGIG9606_50146**	'BGIG9606_50146'	lncRNA	204.66	241.47	208.74	792.55	727.62	560.8
**BGIG9606_50150**	'BGIG9606_50150'	lncRNA	0.04	0.11	0.1	1.8	1.7	1.81
**BGIG9606_50244**	'BGIG9606_50244'	lncRNA	0.93	0.67	0.94	0.32	0.31	0.23
**BGIG9606_50255**	'BGIG9606_50255'	lncRNA	0.78	1.09	0.38	0	0.16	0
**BGIG9606_50499**	'BGIG9606_50499'	lncRNA	0	0	0	0.06	0.17	0.17
**BGIG9606_50502**	'BGIG9606_50502'	lncRNA	127.53	130.46	122.65	395.46	333.34	303.34
**BGIG9606_50503**	'BGIG9606_50503'	lncRNA	283.06	277.48	225.57	877.74	750.59	699.74
**BGIG9606_50523**	'BGIG9606_50523'	lncRNA	6.76	4.65	6.4	3.82	2	1.63
**BGIG9606_50632**	'BGIG9606_50632'	lncRNA	0.53	1.06	0.83	1.65	1.93	1.29
**BGIG9606_50685**	'BGIG9606_50685'	lncRNA	0.26	0.45	0.58	2.73	2.23	3.04
**BGIG9606_50689**	'BGIG9606_50689'	lncRNA	0.04	0.11	0.07	0.95	1.06	1.16
**BGIG9606_51120**	'BGIG9606_51120'	lncRNA	0.35	0.32	0.27	0.09	0.24	0.16
**BGIG9606_51274**	'BGIG9606_51274'	lncRNA	2.95	3.78	3.43	89.55	85	84.15
**BGIG9606_51277**	'BGIG9606_51277'	lncRNA	0.14	0.1	0.19	1.43	1.02	1
**BGIG9606_51485**	'BGIG9606_51485'	lncRNA	0.17	0.4	0.38	0.9	1.07	0.91
**BGIG9606_51636**	'BGIG9606_51636'	lncRNA	0.15	0.21	0.15	0.47	0.34	0.44
**BGIG9606_51777**	'BGIG9606_51777'	lncRNA	0	0.03	0	0.62	0.51	1.04
**BGIG9606_51842**	'BGIG9606_51842'	lncRNA	2.1	1.32	1.81	0.64	1.07	0.53
**BGIG9606_51932**	'BGIG9606_51932'	lncRNA	0.84	0.76	0.64	0.12	0.24	0.5
**BGIG9606_51959**	'BGIG9606_51959'	lncRNA	0.53	0.59	0.8	0.11	0.34	0.29

**BGIG9606_52160**	'BGIG9606_52160'	lncRNA	0	0	0	0.2	0	0.42
**BGIG9606_52169**	'BGIG9606_52169'	lncRNA	0.28	0.19	0.17	0.09	0.07	0.07
**BGIG9606_52334**	'BGIG9606_52334'	lncRNA	0.72	0.53	0.63	0.24	0.32	0.33
**BGIG9606_52376**	'BGIG9606_52376'	lncRNA	18.67	18.7	20.5	8.44	8.85	7.93
**BGIG9606_52475**	'BGIG9606_52475'	lncRNA	0.53	0.66	0.69	0.34	0.24	0.3
**BGIG9606_52605**	'BGIG9606_52605'	lncRNA	0.17	0.24	0.13	0.08	0.12	0.05
**BGIG9606_52667**	'BGIG9606_52667'	lncRNA	0.22	0.25	0.16	0.65	0.73	0.93
**BGIG9606_52668**	'BGIG9606_52668'	lncRNA	0.17	0.2	0.4	0.53	1.1	0.76
**BGIG9606_52677**	'BGIG9606_52677'	lncRNA	0.21	0.16	0.19	0.41	0.52	0.34
**BGIG9606_52719**	'BGIG9606_52719'	lncRNA	0.78	0.68	0.6	0.33	0.33	0.35
**BGIG9606_52799**	'BGIG9606_52799'	lncRNA	0.44	0.33	0.46	1.07	1.56	1.27
**BGIG9606_52815**	'BGIG9606_52815'	lncRNA	0.69	0.59	0.8	2.58	2.37	2.23
**BGIG9606_53022**	'BGIG9606_53022'	lncRNA	4.57	6.4	5.19	1.71	1.88	2.2
**BGIG9606_53220**	'BGIG9606_53220'	lncRNA	0.72	0.68	0.76	0.48	0.36	0.24
**BGIG9606_53563**	'BGIG9606_53563'	lncRNA	0	0	0	0.25	0.05	0.27
**BGIG9606_53894**	'BGIG9606_53894'	lncRNA	0.1	0	0.14	0	0.26	0
**BGIG9606_53966**	'BGIG9606_53966'	lncRNA	1.93	2.34	2.57	0	1.56	1.48
**BGIG9606_54027**	'BGIG9606_54027'	lncRNA	0.18	0.73	0.41	0.28	1.64	0.21
**BGIG9606_54088**	'BGIG9606_54088'	lncRNA	0.19	0	0.4	0	0	0.1
**BGIG9606_54165**	'BGIG9606_54165'	lncRNA	0	0	0	0.76	0	0.29
**BGIG9606_54271**	'BGIG9606_54271'	lncRNA	0.16	0.17	0.34	1.17	0.87	1.16
**BGIG9606_54322**	'BGIG9606_54322'	lncRNA	0	0	0	0	0.58	0.85
**BGIG9606_54493**	'BGIG9606_54493'	lncRNA	4.8	5.04	4.48	1.92	1.81	2
**BGIG9606_54527**	'BGIG9606_54527'	lncRNA	1.99	1.65	1.38	0.61	0.58	0.85
**BGIG9606_54840**	'BGIG9606_54840'	lncRNA	0	0	0.03	0.52	0	0.04
**BGIG9606_55130**	'BGIG9606_55130'	lncRNA	2.64	1.56	2.43	0.61	0.41	0.76
**BGIG9606_55244**	'BGIG9606_55244'	lncRNA	0.22	0.26	0.3	0.21	0.07	0.17
**BGIG9606_55405**	'BGIG9606_55405'	lncRNA	1.61	2.12	1.41	0.81	0.77	0.87
**BGIG9606_55425**	'BGIG9606_55425'	lncRNA	1.38	1.78	0.49	0.03	1.22	0
**BGIG9606_55426**	'BGIG9606_55426'	lncRNA	0.02	0.09	0.29	0.49	1.51	2.17
**BGIG9606_55479**	'BGIG9606_55479'	lncRNA	2.09	0.97	1.03	0.93	0	0.32
**BGIG9606_55726**	'BGIG9606_55726'	lncRNA	0.88	0.87	0.81	0.34	0.3	0.54
**BGIG9606_55853**	'BGIG9606_55853'	lncRNA	0	0	0	0	0.56	0
**BGIG9606_55903**	'BGIG9606_55903'	lncRNA	2.82	1.52	2.76	0	0.81	0
**BGIG9606_55945**	'BGIG9606_55945'	lncRNA	1.66	1.72	1.93	4.79	4.03	3.07
**BGIG9606_55962**	'BGIG9606_55962'	lncRNA	2.13	1.48	1.71	0.97	0.79	0.91
**BGIG9606_56278**	'BGIG9606_56278'	lncRNA	0.28	0.83	0.44	0.16	0.11	0.11
**BGIG9606_56369**	'BGIG9606_56369'	lncRNA	0.6	0.62	0.67	0.13	0.25	0.29
**BGIG9606_56460**	'BGIG9606_56460'	lncRNA	0.77	1.47	0.6	3.63	2.59	4.78

**BGIG9606_56620**	'BGIG9606_56620'	lncRNA	0.18	0.17	0.21	0.05	0	0.04
**BGIG9606_56723**	'BGIG9606_56723'	lncRNA	0.47	0.57	0.34	0.14	0.27	0.1
**BGIG9606_57054**	'BGIG9606_57054'	lncRNA	0.13	0	0.19	2.8	2.26	4.22
**BGIG9606_57198**	'BGIG9606_57198'	lncRNA	0.62	1.01	0.77	0.3	0.37	0.4
**BGIG9606_57261**	'BGIG9606_57261'	lncRNA	0.31	0.4	0.22	0.9	1.03	1.02
**BGIG9606_57378**	'BGIG9606_57378'	lncRNA	2.08	2.08	1.9	0.51	0.93	0.96
**BGIG9606_58225**	'BGIG9606_58225'	lncRNA	0.41	0.44	0.35	0.15	0.16	0.16
**BGIG9606_59632**	'BGIG9606_59632'	lncRNA	0.2	0.18	0.3	0.05	0.07	0.07
**BGIG9606_60571**	'BGIG9606_60571'	lncRNA	0.25	0.07	0.4	2.38	2.07	3.05
**BGIG9606_62621**	'BGIG9606_62621'	lncRNA	0.31	0.33	0.27	0.11	0	0.07
**BGIG9606_62855**	'BGIG9606_62855'	lncRNA	2.11	0.97	1.88	0.7	0.38	0.56
**BGIG9606_62857**	'BGIG9606_62857'	lncRNA	0	0.07	0.07	0.09	0.34	0.83
**BGIG9606_62879**	'BGIG9606_62879'	lncRNA	0.09	0.09	0.1	0.01	0.01	0
**BGIG9606_63238**	'BGIG9606_63238'	lncRNA	1.67	1.01	1.28	0.8	0.51	0.25
**BGIG9606_63291**	'BGIG9606_63291'	lncRNA	3	2.14	3.35	1.32	1.14	1.01
**BGIG9606_64166**	'BGIG9606_64166'	lncRNA	1.69	1.43	1.7	0.6	0.67	0.76
**BGIG9606_64307**	'BGIG9606_64307'	lncRNA	1.25	1.01	1.52	0.35	0.46	0.39
**BGIG9606_64550**	'BGIG9606_64550'	lncRNA	0.06	0	0.03	0.33	0.39	0.39
**BGIG9606_64551**	'BGIG9606_64551'	lncRNA	0.06	0.03	0.06	0.33	0.38	0.27
**BGIG9606_65588**	'BGIG9606_65588'	lncRNA	0.19	0.13	0.2	0.03	0	0.05
**BGIG9606_65731**	'BGIG9606_65731'	lncRNA	6.04	6.41	6.7	2.44	3.55	3.19
**BGIG9606_65774**	'BGIG9606_65774'	lncRNA	0.16	0.1	0.19	0.04	0.05	0.07
**BGIG9606_65840**	'BGIG9606_65840'	lncRNA	0.27	0.28	0.23	0.53	0.99	0.9
**BGIG9606_66067**	'BGIG9606_66067'	lncRNA	0.38	0.39	0.43	0.11	0.16	0.25
**BGIG9606_66208**	'BGIG9606_66208'	lncRNA	0.65	0.53	0.64	0.32	0.22	0.33
**BGIG9606_66247**	'BGIG9606_66247'	lncRNA	0.21	0.17	0.18	0.1	0.04	0.04
**BGIG9606_66253**	'BGIG9606_66253'	lncRNA	0.34	0.44	0.53	0.17	0.1	0.13
**BGIG9606_66310**	'BGIG9606_66310'	lncRNA	0.53	0.16	0.3	0.08	0.08	0.07
**BGIG9606_66342**	'BGIG9606_66342'	lncRNA	0.41	0.32	0.25	0.09	0.19	0.15
**BGIG9606_66508**	'BGIG9606_66508'	lncRNA	0.79	0.51	0.79	0.34	0.16	0.24
**BGIG9606_66731**	'BGIG9606_66731'	lncRNA	0.51	0.8	0.43	1.89	1.32	2.14
**BGIG9606_67618**	'BGIG9606_67618'	lncRNA	0.65	1.05	0.8	0.3	0.33	0.33
**BGIG9606_67671**	'BGIG9606_67671'	lncRNA	0.28	0.38	0.36	1.26	0.4	1.28
**BGIG9606_68033**	'BGIG9606_68033'	lncRNA	0.51	0.25	0.49	0.92	1.4	1.14
**BGIG9606_68234**	'BGIG9606_68234'	lncRNA	1.44	2.53	1.56	0.53	0.79	0.69
**BGIG9606_68829**	'BGIG9606_68829'	lncRNA	0.74	0.91	0.74	0.18	0.42	0.42
**BGIG9606_68844**	'BGIG9606_68844'	lncRNA	0.9	0.78	0.67	2.56	1.63	1.37
