# Supplementary material for: Long non-coding RNA SNHG9 regulates viral replication in rhabdomyosarcoma cells infected with enterovirus D68 via miR-150-5p/c-Fos axis
Source: Front Microbiol. 2023 Jan 19;13:1081237. doi: 10.3389/fmicb.2022.1081237 (PMC9893417; doi:10.3389/fmicb.2022.1081237)
Supplement: Supplementary file 2 [file Data_Sheet_2.PDF]

Supplementary Material

Data Sheet 2 ceRNA screening gene sets

ENTREZID	symbol	gene_class	mirna_pro	cor	pvalue
91947	ARRDC4	mRNA	hsa-miR-30c-1-3p	-0.998456978	3.57E-06
153222	CREBRF	mRNA	hsa-miR-30c-1-3p	-0.998124439	5.27E-06
1839	HBEGF	mRNA	hsa-miR-503-3p	-0.99699499	1.35E-05
7071	KLF10	mRNA	hsa-miR-30c-1-3p	-0.99676548	1.57E-05
91947	ARRDC4	mRNA	hsa-miR-30b-3p	-0.994572617	4.41E-05
57561	ARRDC3	mRNA	hsa-miR-30c-1-3p	-0.994072554	5.26E-05
126068	ZNF441	mRNA	hsa-miR-30c-1-3p	-0.992772909	7.82E-05
7071	KLF10	mRNA	hsa-miR-30b-3p	-0.991624633	0.000104926
57561	ARRDC3	mRNA	hsa-miR-30b-3p	-0.991342942	0.000112093
195828	ZNF367	mRNA	hsa-miR-503-3p	-0.990771192	0.000127363
126068	ZNF441	mRNA	hsa-miR-30b-3p	-0.99070456	0.000129206
153222	CREBRF	mRNA	hsa-miR-30b-3p	-0.989774782	0.000156298

3433	IFIT2	mRNA	hsa-miR-503-3p	-0.988908995	0.000183833
10912	GADD45G	mRNA	hsa-miR-503-3p	-0.984270148	0.000369196
5054	SERPINE1	mRNA	hsa-miR-30b-3p	-0.983796463	0.000391705
23409	SIRT4	mRNA	hsa-miR-503-3p	-0.982844959	0.000438919
6016	RIT1	mRNA	hsa-miR-30c-1-3p	-0.981812442	0.000493173
195828	ZNF367	mRNA	hsa-miR-30c-1-3p	-0.981605199	0.000504441
1960	EGR3	mRNA	hsa-miR-30c-1-3p	-0.981010914	0.000537455
6016	RIT1	mRNA	hsa-miR-30b-3p	-0.980700907	0.000555088
163051	ZNF709	mRNA	hsa-miR-30c-1-3p	-0.97982411	0.000606493
1960	EGR3	mRNA	hsa-miR-30b-3p	-0.977783049	0.000734906
91543	RSAD2	mRNA	hsa-miR-30b-3p	-0.977078162	0.000782094
133	ADM	mRNA	hsa-miR-365b-5p	-0.976533509	0.000819553
5054	SERPINE1	mRNA	hsa-miR-30c-1-3p	-0.975658072	0.000881583
81788	NUAK2	mRNA	hsa-miR-365b-5p	-0.974840456	0.000941541

2353	FOS	mRNA	hsa-miR-503-3p	-0.973470232	0.001046407
64005	MYO1G	mRNA	hsa-miR-103a-2-5p	-0.973269876	0.0010622
402573	C7orf61	mRNA	hsa-miR-103a-2-5p	-0.97284761	0.001095869
153222	CREBRF	mRNA	hsa-miR-503-3p	-0.972661382	0.001110884
57561	ARRDC3	mRNA	hsa-miR-365b-5p	-0.97139791	0.00121542
5292	PIM1	mRNA	hsa-miR-365b-5p	-0.970944136	0.0012541
6016	RIT1	mRNA	hsa-miR-365b-5p	-0.970821696	0.001264639
195828	ZNF367	mRNA	hsa-miR-30b-3p	-0.970775584	0.00126862
282618	IFNL1	mRNA	hsa-miR-503-3p	-0.969787028	0.001355446
80726	IQCN	mRNA	hsa-miR-503-3p	-0.969630117	0.001369489
5292	PIM1	mRNA	hsa-miR-503-3p	-0.969442715	0.001386355
4938	OAS1	mRNA	hsa-miR-548d-5p	-0.968944527	0.001431688
133	ADM	mRNA	hsa-miR-26a-1-3p	-0.968668966	0.001457073
91947	ARRDC4	mRNA	hsa-miR-503-3p	-0.967294996	0.001586935
163059	ZNF433	mRNA	hsa-miR-548d-5p	-0.967285876	0.001587815

80726	IQCN	mRNA	hsa-miR-365b-5p	-0.967044063	0.001611244
5876	RABGGTB	mRNA	hsa-miR-365b-5p	-0.966701747	0.0016447
3725	JUN	mRNA	hsa-miR-503-3p	-0.96628941	0.001685451
55602	CDKN2AIP	mRNA	hsa-miR-503-3p	-0.965021856	0.001813809
100499177	THAP9-AS1	lncRNA	hsa-miR-365b-5p	-0.964744941	0.001842469
3592	IL12A	mRNA	hsa-miR-30c-1-3p	-0.963565955	0.001966977
165904	XIRP1	mRNA	hsa-miR-503-3p	-0.962274853	0.002107935
1490	CTGF	mRNA	hsa-miR-26a-1-3p	-0.961583701	0.00218537
91181	NUP210L	mRNA	hsa-miR-503-3p	-0.960468249	0.00231325
163051	ZNF709	mRNA	hsa-miR-30b-3p	-0.960440691	0.002316454
9510	ADAMTS1	mRNA	hsa-miR-365b-5p	-0.95967048	0.002406908
1843	DUSP1	mRNA	hsa-miR-503-3p	-0.959440605	0.002434235
91543	RSAD2	mRNA	hsa-miR-30c-1-3p	-0.958890438	0.002500257
284391	ZNF844	mRNA	hsa-miR-365b-5p	-0.958759353	0.002516116

388403	YPEL2	mRNA	hsa-miR-30c-1-3p	-0.958696347	0.002523756
3491	CYR61	mRNA	hsa-miR-365b-5p	-0.958600803	0.002535363
27019	DNAI1	mRNA	hsa-miR-548d-5p	-0.95804837	0.002602993
388403	YPEL2	mRNA	hsa-miR-30b-3p	-0.957994593	0.002609623
9510	ADAMTS1	mRNA	hsa-miR-30b-3p	-0.952720002	0.003300252
51277	DNAJC27	mRNA	hsa-miR-365b-5p	-0.952634603	0.00331209
9510	ADAMTS1	mRNA	hsa-miR-30c-1-3p	-0.952381792	0.003347254
55602	CDKN2AIP	mRNA	hsa-miR-548d-5p	-0.952080954	0.003389336
100499177	THAP9-AS1	lncRNA	hsa-miR-30b-3p	-0.95185274	0.003421431
574406	ADAMTSL4-AS1	lncRNA	hsa-miR-503-3p	-0.948543577	0.003903523
64005	MYO1G	mRNA	hsa-miR-365a-5p	-0.948183014	0.003957936
3433	IFIT2	mRNA	hsa-miR-548d-5p	-0.948012495	0.003983798
7568	ZNF20	mRNA	hsa-miR-30c-1-3p	-0.947685808	0.004033576
153222	CREBRF	mRNA	hsa-miR-365b-5p	-0.947265566	0.004098056
100499177	THAP9-AS1	lncRNA	hsa-miR-30c-1-3p	-0.947181795	0.004110969

5292	PIM1	mRNA	hsa-miR-26a-1-3p	-0.945776273	0.004330604
4084	MXD1	mRNA	hsa-miR-365b-5p	-0.945773759	0.004331002
4616	GADD45B	mRNA	hsa-miR-503-3p	-0.945397113	0.004390814
1960	EGR3	mRNA	hsa-miR-503-3p	-0.94302545	0.004776676
3398	ID2	mRNA	hsa-miR-548d-5p	-0.940207205	0.005255883
91543	RSAD2	mRNA	hsa-miR-365a-5p	-0.938936277	0.005479321
84808	PERM1	mRNA	hsa-miR-503-3p	-0.936930184	0.005841263
2353	FOS	mRNA	hsa-miR-26a-1-3p	-0.935383977	0.006127952
4084	MXD1	mRNA	hsa-miR-365a-5p	-0.935165166	0.006169065
91181	NUP210L	mRNA	hsa-miR-548d-5p	-0.934556596	0.006284117
11186	RASSF1	mRNA	hsa-miR-365b-5p	-0.934315276	0.006330027
6528	SLC5A5	mRNA	hsa-miR-503-3p	-0.934109848	0.006369237
81788	NUAK2	mRNA	hsa-miR-26a-1-3p	-0.933693883	0.006448994
149647	FAM71A	mRNA	hsa-miR-365b-5p	-0.932592879	0.00666244

3592	IL12A	mRNA	hsa-miR-30b-3p	-0.931899169	0.006798668
9510	ADAMTS1	mRNA	hsa-miR-503-3p	-0.930689383	0.00703946
7071	KLF10	mRNA	hsa-miR-548d-5p	-0.930641271	0.00704912
153222	CREBRF	mRNA	hsa-miR-26a-1-3p	-0.929814164	0.007216208
7538	ZFP36	mRNA	hsa-miR-548d-5p	-0.929025631	0.00737728
91947	ARRDC4	mRNA	hsa-miR-26a-1-3p	-0.925924458	0.008027546
7538	ZFP36	mRNA	hsa-miR-365b-5p	-0.925684303	0.008079018
91543	RSAD2	mRNA	hsa-miR-503-3p	-0.925422046	0.00813541
29951	PDZRN4	mRNA	hsa-miR-365b-5p	-0.925086449	0.008207851
1843	DUSP1	mRNA	hsa-miR-548d-5p	-0.9240745	0.008428179
126068	ZNF441	mRNA	hsa-miR-548d-5p	-0.923798368	0.008488794
467	ATF3	mRNA	hsa-miR-548d-5p	-0.923724254	0.008505098
688	KLF5	mRNA	hsa-miR-503-3p	-0.923552148	0.00854302
153222	CREBRF	mRNA	hsa-miR-548d-5p	-0.919550217	0.009447909

3725	JUN	mRNA	hsa-miR-548d-5p	-0.918052503	0.009797934
5054	SERPINE1	mRNA	hsa-miR-128-1-5p	-0.917170027	0.010007066
84502	JPH4	mRNA	hsa-miR-30c-1-3p	-0.915783823	0.010339901
1959	EGR2	mRNA	hsa-miR-503-3p	-0.912887016	0.011052472
51421	AMOTL2	mRNA	hsa-miR-503-3p	-0.912529181	0.01114209
388403	YPEL2	mRNA	hsa-miR-548d-5p	-0.912416932	0.011170275
7568	ZNF20	mRNA	hsa-miR-30b-3p	-0.912217975	0.011220316
6665	SOX15	mRNA	hsa-miR-365a-5p	-0.911760221	0.011335859
5876	RABGGTB	mRNA	hsa-miR-548d-5p	-0.911487519	0.011404965
100499177	THAP9-AS1	lncRNA	hsa-miR-503-3p	-0.911399144	0.011427404
4938	OAS1	mRNA	hsa-miR-365b-5p	-0.910618137	0.011626635
1958	EGR1	mRNA	hsa-miR-548d-5p	-0.909815544	0.011833109
4084	MXD1	mRNA	hsa-miR-548d-5p	-0.909733303	0.011854365
284391	ZNF844	mRNA	hsa-miR-26a-1-3p	-0.909151245	0.012005334

126068	ZNF441	mRNA	hsa-miR-26a-1-3p	-0.906393176	0.012733254
91543	RSAD2	mRNA	hsa-miR-128-1-5p	-0.906190248	0.012787629
23767	FLRT3	mRNA	hsa-miR-103a-2-5p	-0.90596994	0.012846788
57561	ARRDC3	mRNA	hsa-miR-548d-5p	-0.905667265	0.01292828
1490	CTGF	mRNA	hsa-miR-210-5p	-0.905392325	0.01300252
64005	MYO1G	mRNA	hsa-miR-503-3p	-0.905217868	0.013049734
4084	MXD1	mRNA	hsa-miR-103a-2-5p	-0.904715426	0.013186174
6016	RIT1	mRNA	hsa-miR-26a-1-3p	-0.904258234	0.013310921
4092	SMAD7	mRNA	hsa-miR-503-3p	-0.903009189	0.013654619
8705	B3GALT4	mRNA	hsa-miR-503-3p	-0.902812164	0.013709221
64005	MYO1G	mRNA	hsa-miR-128-1-5p	-0.901698493	0.014019827
3575	IL7R	mRNA	hsa-miR-365b-5p	-0.901534706	0.01406579
6016	RIT1	mRNA	hsa-miR-365a-5p	-0.90078958	0.014275812
7071	KLF10	mRNA	hsa-miR-26a-1-3p	-0.899985555	0.014504117
7538	ZFP36	mRNA	hsa-miR-365a-5p	-0.899162688	0.01473958

51561	IL23A	mRNA	hsa-miR-503-3p	-0.898944266	0.014802388
84808	PERM1	mRNA	hsa-miR-365a-5p	-0.898820051	0.014838164
91181	NUP210L	mRNA	hsa-miR-365b-5p	-0.898419166	0.014953909
153222	CREBRF	mRNA	hsa-miR-365a-5p	-0.897534034	0.015211002
51277	DNAJC27	mRNA	hsa-miR-548d-5p	-0.897189154	0.015311746
51421	AMOTL2	mRNA	hsa-miR-30b-3p	-0.896901818	0.015395925
51421	AMOTL2	mRNA	hsa-miR-30c-1-3p	-0.896351619	0.015557734
91543	RSAD2	mRNA	hsa-miR-365b-5p	-0.89620863	0.015599919
3491	CYR61	mRNA	hsa-miR-365a-5p	-0.896078432	0.015638378
84502	JPH4	mRNA	hsa-miR-30b-3p	-0.895670902	0.015759053
4084	MXD1	mRNA	hsa-miR-26a-1-3p	-0.893554215	0.016393005
6016	RIT1	mRNA	hsa-miR-548d-5p	-0.892787172	0.016625702
29951	PDZRN4	mRNA	hsa-miR-503-3p	-0.892569991	0.016691874
51421	AMOTL2	mRNA	hsa-miR-26a-1-3p	-0.892170476	0.016813931

26049	FAM169A	mRNA	hsa-miR-30c-1-3p	-0.891916163	0.01689185
10769	PLK2	mRNA	hsa-miR-210-5p	-0.891834272	0.016916977
9120	SLC16A6	mRNA	hsa-miR-503-3p	-0.891635216	0.01697813
5292	PIM1	mRNA	hsa-miR-365a-5p	-0.891361069	0.017062525
284391	ZNF844	mRNA	hsa-miR-365a-5p	-0.88958417	0.017614407
57561	ARRDC3	mRNA	hsa-miR-365a-5p	-0.889011839	0.017793961
23409	SIRT4	mRNA	hsa-miR-26a-1-3p	-0.886589383	0.01856361
26049	FAM169A	mRNA	hsa-miR-30b-3p	-0.88610185	0.018720394
5876	RABGGTB	mRNA	hsa-miR-26a-1-3p	-0.884778742	0.019149072
574406	ADAMTSL4-AS1	lncRNA	hsa-miR-26a-1-3p	-0.883103939	0.01969836
4938	OAS1	mRNA	hsa-miR-365a-5p	-0.881957076	0.020078785
5876	RABGGTB	mRNA	hsa-miR-365a-5p	-0.881911849	0.020093859
26049	FAM169A	mRNA	hsa-miR-26a-1-3p	-0.881580583	0.02020443
4092	SMAD7	mRNA	hsa-miR-548d-5p	-0.880534818	0.020555395
131616	TMEM42	mRNA	hsa-miR-365a-5p	-0.879586501	0.020876154

26049	FAM169A	mRNA	hsa-miR-365a-5p	-0.879478949	0.020912682
6016	RIT1	mRNA	hsa-miR-210-5p	-0.87897304	0.021084915
55603	TENT5A	mRNA	hsa-miR-365b-5p	-0.877601502	0.021555238
80726	IQCN	mRNA	hsa-miR-365a-5p	-0.876689669	0.021870663
11186	RASSF1	mRNA	hsa-miR-365a-5p	-0.876652185	0.021883676
166929	SGMS2	mRNA	hsa-miR-30c-1-3p	-0.87607451	0.022084696
7071	KLF10	mRNA	hsa-miR-103a-2-5p	-0.875761374	0.022194028
6016	RIT1	mRNA	hsa-miR-103a-2-5p	-0.875225217	0.022381826
149647	FAM71A	mRNA	hsa-miR-365a-5p	-0.874102932	0.022777369
131616	TMEM42	mRNA	hsa-miR-103a-2-5p	-0.870408198	0.024102868
91181	NUP210L	mRNA	hsa-miR-365a-5p	-0.87007926	0.024222606
1960	EGR3	mRNA	hsa-miR-548d-5p	-0.870057002	0.024230718
80726	IQCN	mRNA	hsa-miR-210-5p	-0.869753403	0.024341501
284424	MIR7-3HG	lncRNA	hsa-miR-128-1-5p	-0.867478028	0.025179429
29951	PDZRN4	mRNA	hsa-miR-26a-1-3p	-0.867292002	0.025248531

23409	SIRT4	mRNA	hsa-miR-103a-2-5p	-0.866963438	0.025370801
51703	ACSL5	mRNA	hsa-miR-200b-3p	-0.866241	0.025640633
6665	SOX15	mRNA	hsa-miR-548d-5p	-0.86611697	0.025687094
64005	MYO1G	mRNA	hsa-miR-30b-3p	-0.865673132	0.025853684
165904	XIRP1	mRNA	hsa-miR-128-1-5p	-0.8644332	0.026321788
166929	SGMS2	mRNA	hsa-miR-503-3p	-0.861567002	0.027419098
9510	ADAMTS1	mRNA	hsa-miR-365a-5p	-0.856010158	0.029606936
4254	KITLG	mRNA	hsa-miR-365b-5p	-0.855109472	0.029969035
29881	NPC1L1	mRNA	hsa-miR-365b-5p	-0.854857427	0.030070736
10769	PLK2	mRNA	hsa-miR-548d-5p	-0.854828102	0.03008258
126068	ZNF441	mRNA	hsa-miR-210-5p	-0.854652091	0.030153711
91947	ARRDC4	mRNA	hsa-miR-210-5p	-0.852762211	0.030922468
3491	CYR61	mRNA	hsa-miR-128-1-5p	-0.852139348	0.031177837
91543	RSAD2	mRNA	hsa-miR-548d-5p	-0.849619707	0.032220982
5054	SERPINE1	mRNA	hsa-miR-103a-2-5p	-0.847627122	0.033057383

84502	JPH4	mRNA	hsa-miR-503-3p	-0.847224096	0.033227784
3337	DNAJB1	mRNA	hsa-miR-26a-1-3p	-0.846648291	0.033471953
10912	GADD45G	mRNA	hsa-miR-103a-2-5p	-0.846017103	0.033740575
51421	AMOTL2	mRNA	hsa-miR-103a-2-5p	-0.845674675	0.033886729
91181	NUP210L	mRNA	hsa-miR-103a-2-5p	-0.843493713	0.034824577
64005	MYO1G	mRNA	hsa-miR-30c-1-3p	-0.841725175	0.035593915
163051	ZNF709	mRNA	hsa-miR-103a-2-5p	-0.841722251	0.035595194
133	ADM	mRNA	hsa-miR-365a-5p	-0.841106184	0.035865052
27019	DNAI1	mRNA	hsa-miR-503-3p	-0.840835836	0.035983775
100499177	THAP9-AS1	lncRNA	hsa-miR-365a-5p	-0.840683871	0.036050592
7071	KLF10	mRNA	hsa-miR-210-5p	-0.840135972	0.036291978
467	ATF3	mRNA	hsa-miR-103a-2-5p	-0.839306247	0.036658967
400581	GRAPL	mRNA	hsa-miR-103a-2-5p	-0.838769403	0.036897339
3398	ID2	mRNA	hsa-miR-128-1-5p	-0.837865666	0.037300257

100861545	LINC00561	lncRNA	hsa-miR-200b-3p	-0.836985769	0.037694519
2354	FOSB	mRNA	hsa-miR-128-1-5p	-0.836930759	0.037719232
3491	CYR61	mRNA	hsa-miR-103a-2-5p	-0.835296417	0.038456926
4084	MXD1	mRNA	hsa-miR-128-1-5p	-0.834915897	0.038629643
84808	PERM1	mRNA	hsa-miR-365b-5p	-0.833573667	0.039241771
2353	FOS	mRNA	hsa-miR-103a-2-5p	-0.832518294	0.039726242
29881	NPC1L1	mRNA	hsa-miR-503-3p	-0.832169997	0.039886738
1958	EGR1	mRNA	hsa-miR-103a-2-5p	-0.831307741	0.040285373
55603	TENT5A	mRNA	hsa-miR-210-5p	-0.830999544	0.040428307
23645	PPP1R15A	mRNA	hsa-miR-103a-2-5p	-0.829988645	0.040898799
81788	NUAK2	mRNA	hsa-miR-365a-5p	-0.829675298	0.041045153
195828	ZNF367	mRNA	hsa-miR-210-5p	-0.828056134	0.041805306
29881	NPC1L1	mRNA	hsa-miR-365a-5p	-0.827444468	0.042094161
91947	ARRDC4	mRNA	hsa-miR-128-1-5p	-0.827406057	0.042112332

9510	ADAMTS1	mRNA	hsa-miR-128-1-5p	-0.826477283	0.042552804
166929	SGMS2	mRNA	hsa-miR-30b-3p	-0.825695263	0.042925336
103352670	LINC01419	lncRNA	hsa-miR-503-3p	-0.825532052	0.043003277
5778	PTPN7	mRNA	hsa-miR-128-1-5p	-0.824791223	0.043357884
79973	ZNF442	mRNA	hsa-miR-128-1-5p	-0.823854351	0.043808274
29951	PDZRN4	mRNA	hsa-miR-365a-5p	-0.823768598	0.043849607
1490	CTGF	mRNA	hsa-miR-103a-2-5p	-0.823600282	0.043930788
5292	PIM1	mRNA	hsa-miR-103a-2-5p	-0.823152192	0.04414725
153222	CREBRF	mRNA	hsa-miR-103a-2-5p	-0.822310168	0.044555354
51277	DNAJC27	mRNA	hsa-miR-365a-5p	-0.821204304	0.04509399
4616	GADD45B	mRNA	hsa-miR-128-1-5p	-0.820831207	0.045276395
6528	SLC5A5	mRNA	hsa-miR-128-1-5p	-0.820031832	0.045668359
5199	CFP	mRNA	hsa-miR-548d-5p	-0.819547589	0.045906567
163059	ZNF433	mRNA	hsa-miR-103a-2-5p	-0.818981836	0.0461856

64005	MYO1G	mRNA	hsa-miR-365b-5p	-0.816704049	0.047316974
80726	IQCN	mRNA	hsa-miR-103a-2-5p	-0.81645445	0.047441723
10769	PLK2	mRNA	hsa-miR-128-1-5p	-0.816314748	0.047511612
84502	JPH4	mRNA	hsa-miR-365a-5p	-0.815779736	0.047779707
284391	ZNF844	mRNA	hsa-miR-103a-2-5p	-0.815430142	0.047955267
9510	ADAMTS1	mRNA	hsa-miR-103a-2-5p	-0.814767711	0.048288749
9120	SLC16A6	mRNA	hsa-miR-365a-5p	-0.814471672	0.048438128
11186	RASSF1	mRNA	hsa-miR-210-5p	-0.81285731	0.049256488
3575	IL7R	mRNA	hsa-miR-128-1-5p	-0.81253495	0.049420662
105369332	LOC105369332	lncRNA	hsa-miR-210-5p	-0.811297829	0.050053062
153222	CREBRF	mRNA	hsa-miR-128-1-5p	-0.81081514	0.050300818
9120	SLC16A6	mRNA	hsa-miR-365b-5p	-0.810492059	0.050466966
3592	IL12A	mRNA	hsa-miR-26a-1-3p	-0.810319135	0.050555998
91947	ARRDC4	mRNA	hsa-miR-103a-2-5p	-0.810014443	0.05071305
6016	RIT1	mRNA	hsa-miR-128-1-5p	-0.809683193	0.050884047

1843	DUSP1	mRNA	hsa-miR-103a-2-5p	-0.808127508	0.051690682
4254	KITLG	mRNA	hsa-miR-26a-1-3p	-0.807823342	0.05184908
23409	SIRT4	mRNA	hsa-miR-128-1-5p	-0.807429127	0.052054707
109703458	HTD2	mRNA	hsa-miR-150-5p	-0.802967021	0.054408386
131890	GRK7	mRNA	hsa-miR-103a-2-5p	-0.8024613	0.054678175
7538	ZFP36	mRNA	hsa-miR-128-1-5p	-0.800760986	0.055589763
100130155	MIR124-2HG	lncRNA	hsa-miR-200b-3p	-0.800387383	0.055790993
7568	ZNF20	mRNA	hsa-miR-103a-2-5p	-0.800091481	0.055950611
90589	ZNF625	mRNA	hsa-miR-128-1-5p	-0.79955439	0.056240868
29881	NPC1L1	mRNA	hsa-miR-103a-2-5p	-0.799550732	0.056242847
55603	TENT5A	mRNA	hsa-miR-30b-3p	-0.79947008	0.056286493
4254	KITLG	mRNA	hsa-miR-128-1-5p	-0.798435596	0.056847713
1839	HBEGF	mRNA	hsa-miR-128-1-5p	-0.797918642	0.057129127
91543	RSAD2	mRNA	hsa-miR-103a-2-5p	-0.796605373	0.057846907
284391	ZNF844	mRNA	hsa-miR-128-1-5p	-0.796512658	0.057897737

55603	TENT5A	mRNA	hsa-miR-30c-1-3p	-0.791052493	0.060927365
688	KLF5	mRNA	hsa-miR-128-1-5p	-0.789558914	0.061768437
10202	DHRS2	mRNA	hsa-miR-365a-5p	-0.789096712	0.062029785
84502	JPH4	mRNA	hsa-miR-365b-5p	-0.787482908	0.062946262
26049	FAM169A	mRNA	hsa-miR-548d-5p	-0.786785768	0.063344071
1960	EGR3	mRNA	hsa-miR-128-1-5p	-0.78379264	0.06506506
84808	PERM1	mRNA	hsa-miR-103a-2-5p	-0.782391434	0.06587797
3337	DNAJB1	mRNA	hsa-miR-128-1-5p	-0.78214222	0.066023034
109703458	HTD2	mRNA	hsa-miR-30b-3p	-0.782065087	0.066067962
51561	IL23A	mRNA	hsa-miR-103a-2-5p	-0.781096682	0.066633217
688	KLF5	mRNA	hsa-miR-103a-2-5p	-0.779782285	0.067403941
100499177	THAP9-AS1	lncRNA	hsa-miR-548d-5p	-0.779751958	0.067421772
5876	RABGGTB	mRNA	hsa-miR-128-1-5p	-0.779157407	0.067771769
100499177	THAP9-AS1	lncRNA	hsa-miR-103a-2-5p	-0.777331266	0.068851938
109703458	HTD2	mRNA	hsa-miR-30c-1-3p	-0.774362246	0.070624715

10202	DHRS2	mRNA	hsa-miR-503-3p	-0.770537132	0.072938833
166929	SGMS2	mRNA	hsa-miR-365a-5p	-0.770029204	0.073248667
4092	SMAD7	mRNA	hsa-miR-103a-2-5p	-0.769800581	0.073388321
8705	B3GALT4	mRNA	hsa-miR-210-5p	-0.768274998	0.074323307
29951	PDZRN4	mRNA	hsa-miR-128-1-5p	-0.767172114	0.075002574
26049	FAM169A	mRNA	hsa-miR-365b-5p	-0.766791686	0.075237529
6528	SLC5A5	mRNA	hsa-miR-548d-5p	-0.75751805	0.081067578
51421	AMOTL2	mRNA	hsa-miR-128-1-5p	-0.75731779	0.08119564
4254	KITLG	mRNA	hsa-miR-30b-3p	-0.754131165	0.083245658
3304	HSPA1B	mRNA	hsa-miR-103a-2-5p	-0.753077309	0.083928684
51421	AMOTL2	mRNA	hsa-miR-548d-5p	-0.753063612	0.083937578
2354	FOSB	mRNA	hsa-miR-103a-2-5p	-0.750433	0.085653555
3433	IFIT2	mRNA	hsa-miR-128-1-5p	-0.750166722	0.08582812
388403	YPEL2	mRNA	hsa-miR-128-1-5p	-0.749459635	0.086292443
163051	ZNF709	mRNA	hsa-miR-128-1-5p	-0.746460067	0.088274656

4938	OAS1	mRNA	hsa-miR-128-1-5p	-0.744931989	0.089292212
55603	TENT5A	mRNA	hsa-miR-103a-2-5p	-0.742758854	0.090748301
8747	ADAM21	mRNA	hsa-miR-200b-3p	-0.742210416	0.091117441
23228	PLCL2	mRNA	hsa-miR-200b-3p	-0.741529198	0.091576883
55603	TENT5A	mRNA	hsa-miR-128-1-5p	-0.735053093	0.095996075
3575	IL7R	mRNA	hsa-miR-503-3p	-0.732863954	0.097510864
5199	CFP	mRNA	hsa-miR-30c-1-3p	-0.722126022	0.105093048
166929	SGMS2	mRNA	hsa-miR-103a-2-5p	-0.719694949	0.106844469
91181	NUP210L	mRNA	hsa-miR-128-1-5p	-0.71745778	0.108467472
54084	TSPEAR	mRNA	hsa-miR-200b-3p	-0.715454708	0.109929785
4254	KITLG	mRNA	hsa-miR-365a-5p	-0.714670534	0.110504606
5199	CFP	mRNA	hsa-miR-30b-3p	-0.711737231	0.112666477
166929	SGMS2	mRNA	hsa-miR-26a-1-3p	-0.710452963	0.113618782
81571	MIR600HG	lncRNA	hsa-miR-34a-5p	-0.710019097	0.113941295

55603	TENT5A	mRNA	hsa-miR-365a-5p	-0.709174481	0.114570288
4254	KITLG	mRNA	hsa-miR-30c-1-3p	-0.707592349	0.115752598
9120	SLC16A6	mRNA	hsa-miR-26a-1-3p	-0.706168051	0.116821505
43847	KLK14	mRNA	hsa-miR-103a-2-5p	-0.705796797	0.117100831
3575	IL7R	mRNA	hsa-miR-365a-5p	-0.704965767	0.117727141
8705	B3GALT4	mRNA	hsa-miR-103a-2-5p	-0.704495187	0.118082444
166929	SGMS2	mRNA	hsa-miR-365b-5p	-0.702696774	0.119444614
3310	HSPA6	mRNA	hsa-miR-103a-2-5p	-0.69989929	0.121577054
5583	PRKCH	mRNA	hsa-miR-200b-3p	-0.698578975	0.122589203
27019	DNAI1	mRNA	hsa-miR-103a-2-5p	-0.698213633	0.122869918
51277	DNAJC27	mRNA	hsa-miR-128-1-5p	-0.694655618	0.125618366
10202	DHRS2	mRNA	hsa-miR-103a-2-5p	-0.694041556	0.126095382
6665	SOX15	mRNA	hsa-miR-365b-5p	-0.693586159	0.126449651
29951	PDZRN4	mRNA	hsa-miR-103a-2-5p	-0.692755159	0.127097226

3039	HBA1	mRNA	hsa-miR-210-5p	-0.690695332	0.128708574
7368	UGT8	mRNA	hsa-miR-200b-3p	-0.690417488	0.128926598
5199	CFP	mRNA	hsa-miR-503-3p	-0.689591832	0.129575431
150221	RIMBP3C	mRNA	hsa-miR-128-1-5p	-0.686091211	0.132342005
574406	ADAMTSL4-AS1	lncRNA	hsa-miR-128-1-5p	-0.685831638	0.132548154
103352670	LINC01419	lncRNA	hsa-miR-103a-2-5p	-0.685506703	0.132806409
150221	RIMBP3C	mRNA	hsa-miR-503-3p	-0.680819812	0.13655559
10610	ST6GALNAC2	mRNA	hsa-miR-503-3p	-0.680189491	0.137063231
147837	ZNF563	mRNA	hsa-miR-26a-1-3p	-0.676931349	0.13970015
100526836	BLOC1S5-TXNDC5	lncRNA	hsa-miR-200b-3p	-0.671789429	0.14390549
3575	IL7R	mRNA	hsa-miR-103a-2-5p	-0.670746184	0.144765227
80036	TRPM3	mRNA	hsa-miR-200b-3p	-0.670740258	0.144770117
349334	FOXD4L4	mRNA	hsa-miR-200b-3p	-0.664540452	0.14992451
340359	KLHL38	mRNA	hsa-miR-210-5p	-0.662218198	0.15187495

6665	SOX15	mRNA	hsa-miR-210-5p	-0.660684195	0.153169224
3040	HBA2	mRNA	hsa-miR-210-5p	-0.659352604	0.1542965
147837	ZNF563	mRNA	hsa-miR-122-5p	-0.659265689	0.154370202
9120	SLC16A6	mRNA	hsa-miR-128-1-5p	-0.650800691	0.161619527
7568	ZNF20	mRNA	hsa-miR-210-5p	-0.647425851	0.164548809
100130155	MIR124-2HG	lncRNA	hsa-miR-551a	-0.642511782	0.16885363
3433	IFIT2	mRNA	hsa-miR-548av-3p	-0.642081555	0.169232739
7568	ZNF20	mRNA	hsa-miR-128-1-5p	-0.638032723	0.172817932
23767	FLRT3	mRNA	hsa-miR-26a-1-3p	-0.637415972	0.173366817
100505555	LOC100505555	lncRNA	hsa-miR-383-5p	-0.637402399	0.173378904
101929181	LINC01647	lncRNA	hsa-miR-128-1-5p	-0.63639826	0.174274131
29881	NPC1L1	mRNA	hsa-miR-548d-5p	-0.634778299	0.175722443
653160	LOC653160	lncRNA	hsa-miR-200a-3p	-0.633223603	0.177117104
81571	MIR600HG	lncRNA	hsa-miR-200a-3p	-0.631803598	0.178394952

6123	RPL3L	mRNA	hsa-miR-33b-5p	-0.626851469	0.182881171
147837	ZNF563	mRNA	hsa-miR-150-5p	-0.625115767	0.184464506
3303	HSPA1A	mRNA	hsa-miR-103a-2-5p	-0.623878211	0.185596876
25758	KIAA1549L	mRNA	hsa-miR-200b-3p	-0.620649275	0.188564852
23767	FLRT3	mRNA	hsa-miR-1271-3p	-0.608787695	0.199633654
23767	FLRT3	mRNA	hsa-miR-128-1-5p	-0.604135604	0.204045249
100861545	LINC00561	lncRNA	hsa-miR-200a-3p	-0.603684047	0.204475555
166929	SGMS2	mRNA	hsa-miR-33b-5p	-0.600812549	0.207220547
10202	DHRS2	mRNA	hsa-miR-128-1-5p	-0.598443343	0.20949657
166929	SGMS2	mRNA	hsa-miR-1271-3p	-0.592148624	0.215592558
10610	ST6GALNAC2	mRNA	hsa-miR-103a-2-5p	-0.589574962	0.218105283
100505875	LINC01088	lncRNA	hsa-miR-200b-3p	-0.588462732	0.219194807
84981	MIR22HG	lncRNA	hsa-miR-383-5p	-0.586693374	0.220932543
51703	ACSL5	mRNA	hsa-miR-383-5p	-0.584966853	0.222633518

29951	PDZRN4	mRNA	hsa-miR-223-3p	-0.583148384	0.224430738
400804	C1orf140	lncRNA	hsa-miR-200b-3p	-0.583033747	0.22454423
55603	TENT5A	mRNA	hsa-miR-548d-5p	-0.581288307	0.226275064
51421	AMOTL2	mRNA	hsa-miR-548av-3p	-0.579835553	0.227719714
23767	FLRT3	mRNA	hsa-miR-33b-5p	-0.578590711	0.228960533
27019	DNAI1	mRNA	hsa-miR-128-1-5p	-0.57375975	0.233801301
166929	SGMS2	mRNA	hsa-miR-128-1-5p	-0.573211009	0.234353704
1600	DAB1	mRNA	hsa-miR-200a-3p	-0.559857513	0.247954722
54084	TSPEAR	mRNA	hsa-miR-551a	-0.559284079	0.248545543
51277	DNAJC27	mRNA	hsa-miR-548av-3p	-0.553729978	0.254296515
3433	IFIT2	mRNA	hsa-miR-1271-3p	-0.552786474	0.255278568
51703	ACSL5	mRNA	hsa-miR-200a-3p	-0.548141968	0.260134311
340359	KLHL38	mRNA	hsa-miR-1271-3p	-0.547117734	0.261209913
7568	ZNF20	mRNA	hsa-miR-33b-5p	-0.546429666	0.261933456

388531	RGS9BP	mRNA	hsa-miR-34a-5p	-0.544992916	0.263446782
79981	FRMD1	mRNA	hsa-miR-383-5p	-0.542989008	0.26556313
100008586	GAGE12F	mRNA	hsa-miR-200a-3p	-0.541417354	0.267227548
27019	DNAI1	mRNA	hsa-miR-548av-3p	-0.541258	0.267396532
284040	CDRT4	mRNA	hsa-miR-34a-5p	-0.541105138	0.26755867
55076	TMEM45A	mRNA	hsa-miR-200b-3p	-0.540026253	0.268704104
3433	IFIT2	mRNA	hsa-miR-33b-5p	-0.53909361	0.269695795
131616	TMEM42	mRNA	hsa-miR-365b-5p	-0.538569609	0.27025359
80036	TRPM3	mRNA	hsa-miR-551a	-0.536662367	0.272287574
79981	FRMD1	mRNA	hsa-miR-34a-5p	-0.5365082	0.272452242
196541	METTL21C	mRNA	hsa-miR-200b-3p	-0.53627799	0.272698203
163059	ZNF433	mRNA	hsa-miR-548av-3p	-0.534685983	0.274401473
4092	SMAD7	mRNA	hsa-miR-548av-3p	-0.534538737	0.274559215
100526832	PHOSPHO2-KLHL23	mRNA	hsa-miR-200b-3p	-0.531279655	0.278059503
4938	OAS1	mRNA	hsa-miR-548av-3p	-0.529746862	0.279711598

1960	EGR3	mRNA	hsa-miR-122-5p	-0.524171168	0.285752681
150221	RIMBP3C	mRNA	hsa-miR-103a-2-5p	-0.522759957	0.287289455
8747	ADAM21	mRNA	hsa-miR-34a-5p	-0.521102666	0.289098192
1600	DAB1	mRNA	hsa-miR-34a-5p	-0.520563918	0.289687096
10202	DHRS2	mRNA	hsa-miR-365b-5p	-0.519793647	0.290529866
100526832	PHOSPHO2-KLHL23	mRNA	hsa-miR-200a-3p	-0.51959746	0.290744666
6016	RIT1	mRNA	hsa-miR-548av-3p	-0.519078802	0.29131282
5788	PTPRC	mRNA	hsa-miR-128-1-5p	-0.518864825	0.29154734
10769	PLK2	mRNA	hsa-miR-548av-3p	-0.516319239	0.294342768
688	KLF5	mRNA	hsa-miR-223-3p	-0.515993591	0.294701102
4084	MXD1	mRNA	hsa-miR-548av-3p	-0.515851811	0.294857165
100302736	TMED7-TICAM2	mRNA	hsa-miR-503-3p	-0.511851862	0.299272837
54913	RPP25	mRNA	hsa-miR-200a-3p	-0.511778527	0.299354025
100526836	BLOC1S5-TXNDC5	lncRNA	hsa-miR-34a-5p	-0.511351439	0.299827004

3936	LCP1	mRNA	hsa-miR-128-1-5p	-0.509599509	0.301770108
7538	ZFP36	mRNA	hsa-miR-548av-3p	-0.508198813	0.303327026
91181	NUP210L	mRNA	hsa-miR-548av-3p	-0.508063298	0.303477815
28999	KLF15	mRNA	hsa-miR-200a-3p	-0.504904739	0.30700027
27019	DNAI1	mRNA	hsa-miR-1271-3p	-0.504076955	0.307925926
150221	RIMBP3C	mRNA	hsa-miR-150-5p	-0.503428034	0.308652296
150221	RIMBP3C	mRNA	hsa-miR-122-5p	-0.502973506	0.30916145
1960	EGR3	mRNA	hsa-miR-150-5p	-0.502798583	0.309357479
3043	HBB	mRNA	hsa-miR-128-1-5p	-0.502145954	0.310089261
131890	GRK7	mRNA	hsa-miR-1271-3p	-0.500026629	0.312470043
229	ALDOB	mRNA	hsa-miR-34a-5p	-0.498362432	0.314344273
5876	RABGGTB	mRNA	hsa-miR-548av-3p	-0.497627518	0.315173257
3122	HLA-DRA	mRNA	hsa-miR-128-1-5p	-0.497500417	0.315316709
54212	SNTG1	mRNA	hsa-miR-34a-5p	-0.49699118	0.315891698
5199	CFP	mRNA	hsa-miR-103a-2-5p	-0.49486884	0.318292234

80832	APOL4	mRNA	hsa-miR-383-5p	-0.494496064	0.318714565
126068	ZNF441	mRNA	hsa-miR-223-3p	-0.494365889	0.318862093
157807	CLVS1	mRNA	hsa-miR-200b-3p	-0.491673571	0.321918941
3592	IL12A	mRNA	hsa-miR-33b-5p	-0.490506479	0.323247378
389257	LRRC14B	mRNA	hsa-miR-34a-5p	-0.487096541	0.327140192
100526836	BLOC1S5-TXNDC5	lncRNA	hsa-miR-383-5p	-0.486833958	0.327440665
133	ADM	mRNA	hsa-miR-223-3p	-0.486489198	0.327835325
8326	FZD9	mRNA	hsa-miR-34a-5p	-0.485108298	0.329417836
5199	CFP	mRNA	hsa-miR-128-1-5p	-0.484959669	0.329588329
55603	TENT5A	mRNA	hsa-miR-548av-3p	-0.483954823	0.330741844
3398	ID2	mRNA	hsa-miR-548av-3p	-0.483285594	0.3315109
6528	SLC5A5	mRNA	hsa-miR-548av-3p	-0.482596767	0.332303156
229	ALDOB	mRNA	hsa-miR-383-5p	-0.481526851	0.333535083
153222	CREBRF	mRNA	hsa-miR-548av-3p	-0.480219391	0.335042769

1839	HBEGF	mRNA	hsa-miR-1271-3p	-0.479580382	0.335780533
80036	TRPM3	mRNA	hsa-miR-34a-5p	-0.478928557	0.3365337
11186	RASSF1	mRNA	hsa-miR-223-3p	-0.478772509	0.3367141
25758	KIAA1549L	mRNA	hsa-miR-200a-3p	-0.47843228	0.337107543
101929719	LOC101929719	lncRNA	hsa-miR-34a-5p	-0.478119027	0.337469939
7071	KLF10	mRNA	hsa-miR-548av-3p	-0.478071974	0.337524386
80036	TRPM3	mRNA	hsa-miR-383-5p	-0.477587483	0.338085194
388403	YPEL2	mRNA	hsa-miR-548av-3p	-0.477000651	0.338764912
80832	APOL4	mRNA	hsa-miR-200a-3p	-0.47599255	0.339933731
284391	ZNF844	mRNA	hsa-miR-223-3p	-0.473746281	0.342543329
100996335	FAM230H	lncRNA	hsa-miR-34a-5p	-0.473722022	0.342571551
57561	ARRDC3	mRNA	hsa-miR-223-3p	-0.472398144	0.34411297
57561	ARRDC3	mRNA	hsa-miR-548av-3p	-0.469694406	0.347268698
7368	UGT8	mRNA	hsa-miR-383-5p	-0.469630018	0.347343976
131616	TMEM42	mRNA	hsa-miR-26a-1-3p	-0.469473701	0.347526755

1843	DUSP1	mRNA	hsa-miR-548av-3p	-0.468334915	0.348859353
10610	ST6GALNAC2	mRNA	hsa-miR-128-1-5p	-0.467104242	0.350301527
1958	EGR1	mRNA	hsa-miR-548av-3p	-0.465624207	0.352038727
153222	CREBRF	mRNA	hsa-miR-223-3p	-0.464915297	0.352871899
200350	FOXD4L1	mRNA	hsa-miR-200a-3p	-0.464586554	0.353258505
1839	HBEGF	mRNA	hsa-miR-33b-5p	-0.46396598	0.353988716
3725	JUN	mRNA	hsa-miR-548av-3p	-0.463373597	0.354686256
25758	KIAA1549L	mRNA	hsa-miR-34a-5p	-0.463171119	0.354924789
100526832	PHOSPHO2-KLHL23	mRNA	hsa-miR-34a-5p	-0.462819933	0.355338645
1958	EGR1	mRNA	hsa-miR-223-3p	-0.462414543	0.355816592
79639	TMEM53	mRNA	hsa-miR-200a-3p	-0.461486991	0.356911012
341350	OVCH1	mRNA	hsa-miR-34a-5p	-0.460841938	0.357672813
4092	SMAD7	mRNA	hsa-miR-33b-5p	-0.459175777	0.359643195
100533181	FXYD6-FXYD2	mRNA	hsa-miR-34a-5p	-0.458388142	0.360575974

55602	CDKN2AIP	mRNA	hsa-miR-548av-3p	-0.458044423	0.3609833
55602	CDKN2AIP	mRNA	hsa-miR-223-3p	-0.457970448	0.361070986
100271849	MEF2B	mRNA	hsa-miR-34a-5p	-0.457451255	0.361686619
91947	ARRDC4	mRNA	hsa-miR-122-5p	-0.456306833	0.363044925
5778	PTPN7	mRNA	hsa-miR-1271-3p	-0.455845166	0.363593382
100527943	TGIF2-RAB5IF	mRNA	hsa-miR-103a-2-5p	-0.45450841	0.36518308
128497	SPATA25	mRNA	hsa-miR-34a-5p	-0.454432413	0.36527353
3575	IL7R	mRNA	hsa-miR-223-3p	-0.454332133	0.365392894
1960	EGR3	mRNA	hsa-miR-548av-3p	-0.454003493	0.365784173
972	CD74	mRNA	hsa-miR-128-1-5p	-0.453755715	0.366079273
133121	ENPP6	mRNA	hsa-miR-34a-5p	-0.452185447	0.367951388
93107	KCNG4	mRNA	hsa-miR-383-5p	-0.450798814	0.369607349
56603	CYP26B1	mRNA	hsa-miR-200a-3p	-0.450081059	0.370465538
5583	PRKCH	mRNA	hsa-miR-200a-3p	-0.449993835	0.370569875

467	ATF3	mRNA	hsa-miR-548av-3p	-0.447867705	0.373116322
6514	SLC2A2	mRNA	hsa-miR-383-5p	-0.447672236	0.373350739
574406	ADAMTSL4-AS1	lncRNA	hsa-miR-1271-3p	-0.447542004	0.373506948
5788	PTPRC	mRNA	hsa-miR-103a-2-5p	-0.447304519	0.373791863
4254	KITLG	mRNA	hsa-miR-548d-5p	-0.447045064	0.374103223
284391	ZNF844	mRNA	hsa-miR-122-5p	-0.445977752	0.375385002
3936	LCP1	mRNA	hsa-miR-103a-2-5p	-0.445355185	0.376133373
133121	ENPP6	mRNA	hsa-miR-383-5p	-0.444669856	0.376957786
1959	EGR2	mRNA	hsa-miR-122-5p	-0.444124629	0.377614112
100137049	PLA2G4B	mRNA	hsa-miR-34a-5p	-0.442440395	0.379644036
8705	B3GALT4	mRNA	hsa-miR-150-5p	-0.4418102	0.380404548
220980	TMEM72-AS1	lncRNA	hsa-miR-34a-5p	-0.441670779	0.38057287
57561	ARRDC3	mRNA	hsa-miR-122-5p	-0.441492	0.380788747
54913	RPP25	mRNA	hsa-miR-34a-5p	-0.441003209	0.381379183

8747	ADAM21	mRNA	hsa-miR-200a-3p	-0.440177805	0.382376948
10202	DHRS2	mRNA	hsa-miR-1271-3p	-0.438546392	0.384351677
4092	SMAD7	mRNA	hsa-miR-1271-3p	-0.438531047	0.384370268
5583	PRKCH	mRNA	hsa-miR-34a-5p	-0.43564157	0.387876453
153222	CREBRF	mRNA	hsa-miR-122-5p	-0.435487553	0.388063649
80726	IQCN	mRNA	hsa-miR-122-5p	-0.435081594	0.388557211
5657	PRTN3	mRNA	hsa-miR-128-1-5p	-0.434995345	0.388662099
100996335	FAM230H	lncRNA	hsa-miR-383-5p	-0.434653074	0.389078434
100137049	PLA2G4B	mRNA	hsa-miR-383-5p	-0.433352076	0.39066235
51421	AMOTL2	mRNA	hsa-miR-1271-3p	-0.433294662	0.3907323
80036	TRPM3	mRNA	hsa-miR-200a-3p	-0.432963448	0.391135918
91543	RSAD2	mRNA	hsa-miR-122-5p	-0.432379136	0.391848306
103352670	LINC01419	lncRNA	hsa-miR-33b-5p	-0.430121097	0.394605451
1843	DUSP1	mRNA	hsa-miR-122-5p	-0.429702706	0.395117043

163051	ZNF709	mRNA	hsa-miR-33b-5p	-0.428770643	0.396257547
81788	NUAK2	mRNA	hsa-miR-122-5p	-0.42864942	0.396405962
100507257	MEG9	lncRNA	hsa-miR-383-5p	-0.426334796	0.399243401
100507257	MEG9	lncRNA	hsa-miR-34a-5p	-0.425949178	0.399716788
340267	COL28A1	mRNA	hsa-miR-200a-3p	-0.425804784	0.399894095
5583	PRKCH	mRNA	hsa-miR-383-5p	-0.425025714	0.400851209
91947	ARRDC4	mRNA	hsa-miR-150-5p	-0.424908192	0.400995656
105369332	LOC105369332	lncRNA	hsa-miR-150-5p	-0.424411333	0.401606542
11186	RASSF1	mRNA	hsa-miR-150-5p	-0.424380229	0.401644795
5292	PIM1	mRNA	hsa-miR-150-5p	-0.422713721	0.403696119
2353	FOS	mRNA	hsa-miR-122-5p	-0.421703837	0.404940911
5876	RABGGTB	mRNA	hsa-miR-223-3p	-0.420988773	0.405823086
100499177	THAP9-AS1	lncRNA	hsa-miR-548av-3p	-0.419973913	0.407076229
51277	DNAJC27	mRNA	hsa-miR-1271-3p	-0.419060535	0.408205171

4938	OAS1	mRNA	hsa-miR-1271-3p	- 0.418971587	0.408315167
7368	UGT8	mRNA	hsa-miR-200a-3p	- 0.418090457	0.409405343
101929260	LINC01920	lncRNA	hsa-miR-34a-5p	- 0.417947272	0.40958259
81788	NUAK2	mRNA	hsa-miR-150-5p	- 0.415487723	0.412631248
284391	ZNF844	mRNA	hsa-miR-150-5p	- 0.414805583	0.413478111
3491	CYR61	mRNA	hsa-miR-122-5p	- 0.412721455	0.416069097
81788	NUAK2	mRNA	hsa-miR-1271-3p	- 0.412284535	0.416612958
3936	LCP1	mRNA	hsa-miR-30b-3p	- 0.411724935	0.417309873
1959	EGR2	mRNA	hsa-miR-150-5p	- 0.410025317	0.419428909
153222	CREBRF	mRNA	hsa-miR-150-5p	- 0.409607048	0.41995094
3575	IL7R	mRNA	hsa-miR-122-5p	- 0.408735557	0.421039318
109703458	HTD2	mRNA	hsa-miR-103a-2-5p	- 0.406129163	0.424299909
163059	ZNF433	mRNA	hsa-miR-33b-5p	- 0.405375209	0.42524465
1958	EGR1	mRNA	hsa-miR-150-5p	- 0.404474943	0.426373631

126068	ZNF441	mRNA	hsa-miR-548av-3p	-0.403071908	0.428135072
9510	ADAMTS1	mRNA	hsa-miR-150-5p	-0.402433301	0.428937601
101929268	LOC101929268	lncRNA	hsa-miR-34a-5p	-0.399246115	0.432950236
79973	ZNF442	mRNA	hsa-miR-150-5p	-0.399114645	0.433116018
100505875	LINC01088	lncRNA	hsa-miR-34a-5p	-0.399072515	0.433169146
51277	DNAJC27	mRNA	hsa-miR-33b-5p	-0.398732678	0.433597788
81571	MIR600HG	lncRNA	hsa-miR-551a	-0.397966715	0.434564416
4938	OAS1	mRNA	hsa-miR-122-5p	-0.397913379	0.43463175
100505768	LINC01364	lncRNA	hsa-miR-200a-3p	-0.39751586	0.435133712
91181	NUP210L	mRNA	hsa-miR-1271-3p	-0.396755067	0.436094916
400581	GRAPL	mRNA	hsa-miR-150-5p	-0.395858628	0.437228384
968	CD68	mRNA	hsa-miR-122-5p	-0.394669829	0.438732987
5199	CFP	mRNA	hsa-miR-122-5p	-0.391711887	0.442483953
3575	IL7R	mRNA	hsa-miR-150-5p	-0.391663051	0.442545969

84808	PERM1	mRNA	hsa-miR-122-5p	-0.391607044	0.442617094
5876	RABGGTB	mRNA	hsa-miR-122-5p	-0.390368575	0.444190808
7538	ZFP36	mRNA	hsa-miR-1271-3p	-0.390027381	0.444624676
388403	YPEL2	mRNA	hsa-miR-223-3p	-0.389860303	0.444837186
11186	RASSF1	mRNA	hsa-miR-451a	-0.387647298	0.447655015
91543	RSAD2	mRNA	hsa-miR-150-5p	-0.386860477	0.448658253
349334	FOXD4L4	mRNA	hsa-miR-34a-5p	-0.38622139	0.449473651
23409	SIRT4	mRNA	hsa-miR-223-3p	-0.385713932	0.450121443
6016	RIT1	mRNA	hsa-miR-1271-3p	-0.384180043	0.452081328
8941	CDK5R2	mRNA	hsa-miR-34a-5p	-0.383740108	0.452643945
5199	CFP	mRNA	hsa-miR-223-3p	-0.382468466	0.454271452
3592	IL12A	mRNA	hsa-miR-122-5p	-0.381701638	0.455253771
100527943	TGIF2-RAB5IF	mRNA	hsa-miR-33b-5p	-0.381621518	0.455356445
23645	PPP1R15A	mRNA	hsa-miR-150-5p	-0.380689894	0.456550862

3310	HSPA6	mRNA	hsa-miR-223-3p	-0.380651897	0.456599597
3040	HBA2	mRNA	hsa-miR-34a-5p	-0.379027941	0.458684079
101929181	LINC01647	lncRNA	hsa-miR-122-5p	-0.378908479	0.458837537
100526740	ATP5MF-PTCD1	mRNA	hsa-miR-383-5p	-0.37829723	0.459622985
29881	NPC1L1	mRNA	hsa-miR-548av-3p	-0.377083134	0.461184343
3398	ID2	mRNA	hsa-miR-1271-3p	-0.376831007	0.461508793
968	CD68	mRNA	hsa-miR-150-5p	-0.375698945	0.462966478
6016	RIT1	mRNA	hsa-miR-33b-5p	-0.375604301	0.463088412
10769	PLK2	mRNA	hsa-miR-1271-3p	-0.374015059	0.465137383
10769	PLK2	mRNA	hsa-miR-33b-5p	-0.373909313	0.46527382
2826	CCR10	mRNA	hsa-miR-34a-5p	-0.372386889	0.467239483
7071	KLF10	mRNA	hsa-miR-223-3p	-0.371719952	0.468101409
400932	LINC00898	lncRNA	hsa-miR-34a-5p	-0.371660627	0.468178102
3337	DNAJB1	mRNA	hsa-miR-122-5p	-0.371300277	0.468644036

54937	SOHLH2	mRNA	hsa-miR-34a-5p	-0.371160643	0.468824622
26049	FAM169A	mRNA	hsa-miR-223-3p	-0.369186049	0.471380649
3936	LCP1	mRNA	hsa-miR-30c-1-3p	-0.369166529	0.471405939
4938	OAS1	mRNA	hsa-miR-150-5p	-0.367606264	0.473428724
5788	PTPRC	mRNA	hsa-miR-383-5p	-0.36757792	0.473465495
8941	CDK5R2	mRNA	hsa-miR-383-5p	-0.367171842	0.473992403
91181	NUP210L	mRNA	hsa-miR-33b-5p	-0.367086082	0.474103705
11186	RASSF1	mRNA	hsa-miR-1271-3p	-0.366450937	0.474928263
90589	ZNF625	mRNA	hsa-miR-150-5p	-0.363321128	0.47899791
3303	HSPA1A	mRNA	hsa-miR-150-5p	-0.362530241	0.480027983
349334	FOXD4L4	mRNA	hsa-miR-200a-3p	-0.361706375	0.481101732
7071	KLF10	mRNA	hsa-miR-1271-3p	-0.36165521	0.48116844
5876	RABGGTB	mRNA	hsa-miR-150-5p	-0.359830843	0.483548866
23645	PPP1R15A	mRNA	hsa-miR-1271-3p	-0.358633978	0.485112485
1843	DUSP1	mRNA	hsa-miR-33b-5p	-0.35858256	0.485179694

55603	TENT5A	mRNA	hsa-miR-33b-5p	-0.358147597	0.485748347
3310	HSPA6	mRNA	hsa-miR-33b-5p	-0.356884932	0.487400258
84808	PERM1	mRNA	hsa-miR-150-5p	-0.356306154	0.488158028
3592	IL12A	mRNA	hsa-miR-150-5p	-0.355302615	0.489472769
153222	CREBRF	mRNA	hsa-miR-33b-5p	-0.354391376	0.490667518
90589	ZNF625	mRNA	hsa-miR-33b-5p	-0.354272049	0.490824036
153222	CREBRF	mRNA	hsa-miR-1271-3p	-0.353532598	0.491794292
4616	GADD45B	mRNA	hsa-miR-150-5p	-0.353530059	0.491797624
56603	CYP26B1	mRNA	hsa-miR-383-5p	-0.353306516	0.492091056
51561	IL23A	mRNA	hsa-miR-223-3p	-0.352277892	0.493441955
972	CD74	mRNA	hsa-miR-503-3p	-0.351094331	0.494997716
55603	TENT5A	mRNA	hsa-miR-223-3p	-0.349695953	0.496837751
388403	YPEL2	mRNA	hsa-miR-1271-3p	-0.349048851	0.497689924
151516	ASPRV1	mRNA	hsa-miR-34a-5p	-0.3484609	0.498464582

9510	ADAMTS1	mRNA	hsa-miR-33b-5p	-0.34800116	0.499070566
2353	FOS	mRNA	hsa-miR-33b-5p	-0.346027676	0.501674324
6016	RIT1	mRNA	hsa-miR-122-5p	-0.345995856	0.50171634
2353	FOS	mRNA	hsa-miR-1271-3p	-0.344080927	0.50424677
3725	JUN	mRNA	hsa-miR-1271-3p	-0.343146284	0.505483203
55602	CDKN2AIP	mRNA	hsa-miR-33b-5p	-0.340776555	0.50862213
51277	DNAJC27	mRNA	hsa-miR-150-5p	-0.337946799	0.512377922
84808	PERM1	mRNA	hsa-miR-1271-3p	-0.337907856	0.512429666
3337	DNAJB1	mRNA	hsa-miR-1271-3p	-0.337452849	0.513034351
3310	HSPA6	mRNA	hsa-miR-1271-3p	-0.336888468	0.51378468
163059	ZNF433	mRNA	hsa-miR-150-5p	-0.336833228	0.513858138
5054	SERPINE1	mRNA	hsa-miR-150-5p	-0.336718141	0.514011189
23409	SIRT4	mRNA	hsa-miR-33b-5p	-0.336002709	0.514962924
84808	PERM1	mRNA	hsa-miR-33b-5p	-0.334548659	0.516898823

9510	ADAMTS1	mRNA	hsa-miR-1271-3p	-0.334320855	0.517202311
55076	TMEM45A	mRNA	hsa-miR-200a-3p	-0.334250168	0.517296492
5199	CFP	mRNA	hsa-miR-150-5p	-0.333876872	0.517793948
57561	ARRDC3	mRNA	hsa-miR-1271-3p	-0.333240242	0.518642644
5657	PRTN3	mRNA	hsa-miR-34a-5p	-0.333068709	0.518871385
79973	ZNF442	mRNA	hsa-miR-33b-5p	-0.332527725	0.519592987
103352670	LINC01419	lncRNA	hsa-miR-122-5p	-0.331191985	0.521375937
51421	AMOTL2	mRNA	hsa-miR-223-3p	-0.329267478	0.52394789
80726	IQCN	mRNA	hsa-miR-1271-3p	-0.328402638	0.525104875
3491	CYR61	mRNA	hsa-miR-33b-5p	-0.327361353	0.526498884
6528	SLC5A5	mRNA	hsa-miR-33b-5p	-0.32690795	0.527106207
388403	YPEL2	mRNA	hsa-miR-150-5p	-0.326299727	0.527921222
9120	SLC16A6	mRNA	hsa-miR-150-5p	-0.326273282	0.527956667
5292	PIM1	mRNA	hsa-miR-33b-5p	-0.325311582	0.529246104

100302736	TMED7-TICAM2	mRNA	hsa-miR-1271-3p	-0.324983696	0.529685936
165904	XIRP1	mRNA	hsa-miR-122-5p	-0.324597318	0.530204365
26049	FAM169A	mRNA	hsa-miR-150-5p	-0.322783499	0.532640027
23409	SIRT4	mRNA	hsa-miR-150-5p	-0.321783666	0.533984002
26049	FAM169A	mRNA	hsa-miR-548av-3p	-0.32092371	0.535140727
149647	FAM71A	mRNA	hsa-miR-33b-5p	-0.320109084	0.536237135
688	KLF5	mRNA	hsa-miR-1271-3p	-0.31891157	0.53785003
284391	ZNF844	mRNA	hsa-miR-33b-5p	-0.318747865	0.538070626
80726	IQCN	mRNA	hsa-miR-33b-5p	-0.315658432	0.542238494
1490	CTGF	mRNA	hsa-miR-1271-3p	-0.313561301	0.545072831
100506071	LOC100506071	lncRNA	hsa-miR-383-5p	-0.309933094	0.549986217
400804	C1orf140	lncRNA	hsa-miR-200a-3p	-0.307251752	0.553625214
1960	EGR3	mRNA	hsa-miR-1271-3p	-0.306712523	0.554357833
55603	TENT5A	mRNA	hsa-miR-150-5p	-0.305956793	0.55538505
51561	IL23A	mRNA	hsa-miR-150-5p	-0.30504048	0.556631242

968	CD68	mRNA	hsa-miR-451a	-0.300037116	0.563449337
55603	TENT5A	mRNA	hsa-miR-122-5p	-0.298838883	0.565085531
91947	ARRDC4	mRNA	hsa-miR-1271-3p	-0.294373864	0.57119383
84502	JPH4	mRNA	hsa-miR-33b-5p	-0.294321471	0.571265612
4084	MXD1	mRNA	hsa-miR-122-5p	-0.293995384	0.571712418
3039	HBA1	mRNA	hsa-miR-34a-5p	-0.292211209	0.574158762
64005	MYO1G	mRNA	hsa-miR-1271-3p	-0.291925532	0.574550725
91947	ARRDC4	mRNA	hsa-miR-33b-5p	-0.29161399	0.574978255
166929	SGMS2	mRNA	hsa-miR-223-3p	-0.285382547	0.583547413
6665	SOX15	mRNA	hsa-miR-122-5p	-0.283777041	0.585760637
284424	MIR7-3HG	lncRNA	hsa-miR-223-3p	-0.278464204	0.593100073
29881	NPC1L1	mRNA	hsa-miR-122-5p	-0.276597126	0.595684977
51421	AMOTL2	mRNA	hsa-miR-150-5p	-0.276291799	0.596107967
340359	KLHL38	mRNA	hsa-miR-223-3p	-0.275427689	0.597305495

3040	HBA2	mRNA	hsa-miR-551a	-0.27319614	0.600400941
6123	RPL3L	mRNA	hsa-miR-150-5p	-0.268850362	0.606440778
126068	ZNF441	mRNA	hsa-miR-33b-5p	-0.266913862	0.609137081
4084	MXD1	mRNA	hsa-miR-150-5p	-0.265107782	0.611654498
3398	ID2	mRNA	hsa-miR-150-5p	-0.263807921	0.613467925
26049	FAM169A	mRNA	hsa-miR-33b-5p	-0.256739584	0.623352146
166929	SGMS2	mRNA	hsa-miR-150-5p	-0.254401505	0.626630191
27019	DNAI1	mRNA	hsa-miR-122-5p	-0.250796233	0.631693036
5788	PTPRC	mRNA	hsa-miR-223-3p	-0.246739589	0.637401423
29881	NPC1L1	mRNA	hsa-miR-1271-3p	-0.24566756	0.638911992
4254	KITLG	mRNA	hsa-miR-548av-3p	-0.241331957	0.645029786
6528	SLC5A5	mRNA	hsa-miR-223-3p	-0.240664156	0.645973308
6665	SOX15	mRNA	hsa-miR-150-5p	-0.234595424	0.654562345
131890	GRK7	mRNA	hsa-miR-122-5p	-0.233776681	0.655723106
284424	MIR7-3HG	lncRNA	hsa-miR-150-5p	-0.227660048	0.664409635

340359	KLHL38	mRNA	hsa-miR-150-5p	-0.224155597	0.669398036
27019	DNAI1	mRNA	hsa-miR-150-5p	-0.220480002	0.674638921
3039	HBA1	mRNA	hsa-miR-551a	-0.219814151	0.675589292
100302736	TMED7-TICAM2	mRNA	hsa-miR-122-5p	-0.21939204	0.676191924
5788	PTPRC	mRNA	hsa-miR-150-5p	-0.215081396	0.682352739
6665	SOX15	mRNA	hsa-miR-548av-3p	-0.210354563	0.689122149
131890	GRK7	mRNA	hsa-miR-150-5p	-0.210036611	0.689578005
91543	RSAD2	mRNA	hsa-miR-548av-3p	-0.209587609	0.690221861
5054	SERPINE1	mRNA	hsa-miR-33b-5p	-0.208422586	0.691893058
157807	CLVS1	mRNA	hsa-miR-383-5p	-0.207608052	0.69306199
4254	KITLG	mRNA	hsa-miR-150-5p	-0.202099296	0.700978341
100302736	TMED7-TICAM2	mRNA	hsa-miR-150-5p	-0.200798374	0.702850532
5778	PTPN7	mRNA	hsa-miR-122-5p	-0.19548683	0.71050503
150221	RIMBP3C	mRNA	hsa-miR-1271-3p	-0.194388504	0.712089913

3936	LCP1	mRNA	hsa-miR-548av-3p	-0.191813299	0.715808681
3936	LCP1	mRNA	hsa-miR-223-3p	-0.187215961	0.722457001
5199	CFP	mRNA	hsa-miR-548av-3p	-0.182019812	0.72998555
101929181	LINC01647	lncRNA	hsa-miR-1271-3p	-0.178853384	0.734580553
5778	PTPN7	mRNA	hsa-miR-150-5p	-0.177033968	0.73722326
10610	ST6GALNAC2	mRNA	hsa-miR-122-5p	-0.174779621	0.740500145
166929	SGMS2	mRNA	hsa-miR-451a	-0.169691753	0.747905532
5657	PRTN3	mRNA	hsa-miR-122-5p	-0.169022031	0.748881302
972	CD74	mRNA	hsa-miR-223-3p	-0.166846803	0.752052124
10610	ST6GALNAC2	mRNA	hsa-miR-1271-3p	-0.16023483	0.761704785
3936	LCP1	mRNA	hsa-miR-150-5p	-0.15510893	0.769202471
131616	TMEM42	mRNA	hsa-miR-1271-3p	-0.139291839	0.792413527
972	CD74	mRNA	hsa-miR-150-5p	-0.138940234	0.792930727
200350	FOXD4L1	mRNA	hsa-miR-1271-3p	-0.133831618	0.800451095
3936	LCP1	mRNA	hsa-miR-122-5p	-0.133033567	0.801626859

5199	CFP	mRNA	hsa-miR-1271-3p	-0.129381825	0.807010166
29951	PDZRN4	mRNA	hsa-miR-1271-3p	-0.124777769	0.81380471
11172	INSL6	mRNA	hsa-miR-33b-5p	-0.123845689	0.815181224
3122	HLA-DRA	mRNA	hsa-miR-33b-5p	-0.119698117	0.82131032
10610	ST6GALNAC2	mRNA	hsa-miR-150-5p	-0.117523701	0.824526057
5788	PTPRC	mRNA	hsa-miR-200a-3p	-0.116090865	0.826645986
972	CD74	mRNA	hsa-miR-122-5p	-0.112870764	0.831412829
10202	DHRS2	mRNA	hsa-miR-223-3p	-0.108820151	0.837414088
29951	PDZRN4	mRNA	hsa-miR-33b-5p	-0.106591288	0.840718597
10202	DHRS2	mRNA	hsa-miR-122-5p	-0.105276615	0.842668477
3433	IFIT2	mRNA	hsa-miR-150-5p	-0.102394813	0.84694457
972	CD74	mRNA	hsa-miR-33b-5p	-0.099651772	0.851017137
147837	ZNF563	mRNA	hsa-miR-33b-5p	-0.098566052	0.85262972
10610	ST6GALNAC2	mRNA	hsa-miR-33b-5p	-0.096661466	0.855459376

3936	LCP1	mRNA	hsa-miR-200b-3p	-0.093000809	0.860900976
91543	RSAD2	mRNA	hsa-miR-1271-3p	-0.089041734	0.866790379
5788	PTPRC	mRNA	hsa-miR-33b-5p	-0.086134391	0.871117934
4254	KITLG	mRNA	hsa-miR-1271-3p	-0.080267637	0.879857122
4254	KITLG	mRNA	hsa-miR-33b-5p	-0.079047579	0.881675597
4254	KITLG	mRNA	hsa-miR-34a-5p	-0.078950396	0.881820461
972	CD74	mRNA	hsa-miR-1271-3p	-0.073395607	0.890104278
3936	LCP1	mRNA	hsa-miR-33b-5p	-0.073198065	0.890398999
10202	DHRS2	mRNA	hsa-miR-150-5p	-0.072655219	0.891208938
100526836	BLOC1S5-TXNDC5	lncRNA	hsa-miR-1271-3p	-0.071374691	0.893119767
3936	LCP1	mRNA	hsa-miR-548d-5p	-0.067461529	0.898961218
101929268	LOC101929268	lncRNA	hsa-miR-1271-3p	-0.066200129	0.900844867
3433	IFIT2	mRNA	hsa-miR-451a	-0.064984853	0.902659936
341350	OVCH1	mRNA	hsa-miR-33b-5p	-0.061058072	0.908526707

81571	MIR600HG	lncRNA	hsa-miR-451a	-0.057486056	0.913865902
3575	IL7R	mRNA	hsa-miR-383-5p	-0.054830865	0.917836125
100526836	BLOC1S5-TXNDC5	lncRNA	hsa-miR-33b-5p	-0.054053484	0.918998741
3936	LCP1	mRNA	hsa-miR-1271-3p	-0.047734221	0.92845305
105374676	LINC02241	lncRNA	hsa-miR-33b-5p	-0.04000924	0.940018162
3575	IL7R	mRNA	hsa-miR-34a-5p	-0.030620956	0.954082921
64005	MYO1G	mRNA	hsa-miR-223-3p	-0.017697422	0.973456638
100271849	MEF2B	mRNA	hsa-miR-128-1-5p	-0.015483373	0.976776796
100996335	FAM230H	lncRNA	hsa-miR-150-5p	-0.006391762	0.990412487
222537	HS3ST5	mRNA	hsa-miR-150-5p	-0.003015833	0.995476264
