# Supplementary material for: Long non-coding RNA SNHG9 regulates viral replication in rhabdomyosarcoma cells infected with enterovirus D68 via miR-150-5p/c-Fos axis
Source: Front Microbiol. 2023 Jan 19;13:1081237. doi: 10.3389/fmicb.2022.1081237 (PMC9893417; doi:10.3389/fmicb.2022.1081237)
Supplement: Supplementary file 3 [file Data_Sheet_3.PDF]

Supplementary Material

Data Sheet 3 lncRNA-mRNA co-expressed gene sets

fromNode	toNode
XIRP1	ZNF625-ZNF20
TSNAX-DISC1	ZNF625-ZNF20
THAP9-AS1	ZNF625-ZNF20
TCL6	ZNF625-ZNF20
TALAM1	ZNF625-ZNF20
RASSF1	ZNF625-ZNF20
PWAR1	ZNF625-ZNF20
NUAK2	ZNF625-ZNF20
MIR600HG	ZNF625-ZNF20
MIR22HG	ZNF625-ZNF20
MIR133A1HG	ZNF625-ZNF20
MEG9	ZNF625-ZNF20
MEG8	ZNF625-ZNF20
LINC02241	ZNF625-ZNF20
LINC01852	ZNF625-ZNF20
LINC01647	ZNF625-ZNF20

LINC01419	ZNF625-ZNF20
LINC01341	ZNF625-ZNF20
LINC01088	ZNF625-ZNF20
LINC00597	ZNF625-ZNF20
LINC00312	ZNF625-ZNF20
KCNH1-IT1	ZNF625-ZNF20
ITGA6-AS1	ZNF625-ZNF20
HAS2-AS1	ZNF625-ZNF20
DARS-AS1	ZNF625-ZNF20
CYP26B1	ZNF625-ZNF20
C5orf17	ZNF625-ZNF20
BORCS7-ASMT	ZNF625-ZNF20
BLOC1S5-TXNDC5	ZNF625-ZNF20
THAP9-AS1	XIRP1
TCL6	XIRP1
RASSF1	XIRP1
PWAR1	XIRP1
NUAK2	XIRP1
MIR600HG	XIRP1
MIR133A1HG	XIRP1

LINC01852	XIRP1
LINC01647	XIRP1
LINC00312	XIRP1
KCNH1-IT1	XIRP1
DARS-AS1	XIRP1
CYP26B1	XIRP1
C5orf17	XIRP1
BLOC1S5-TXNDC5	XIRP1
XIRP1	TSNAX-DISC1
THAP9-AS1	TSNAX-DISC1
TCL6	TSNAX-DISC1
RASSF1	TSNAX-DISC1
PWAR1	TSNAX-DISC1
NUAK2	TSNAX-DISC1
MIR600HG	TSNAX-DISC1
MIR22HG	TSNAX-DISC1
MIR133A1HG	TSNAX-DISC1
MEG9	TSNAX-DISC1
MEG8	TSNAX-DISC1
LINC01852	TSNAX-DISC1

LINC01647	TSNAX-DISC1
LINC01419	TSNAX-DISC1
LINC01088	TSNAX-DISC1
LINC00312	TSNAX-DISC1
KCNH1-IT1	TSNAX-DISC1
ITGA6-AS1	TSNAX-DISC1
HAS2-AS1	TSNAX-DISC1
DARS-AS1	TSNAX-DISC1
CYP26B1	TSNAX-DISC1
C5orf17	TSNAX-DISC1
BORCS7-ASMT	TSNAX-DISC1
BLOC1S5-TXNDC5	TSNAX-DISC1
ZNF625-ZNF20	TMEM72-AS1
XIRP1	TMEM72-AS1
TSNAX-DISC1	TMEM72-AS1
THAP9-AS1	TMEM72-AS1
TCL6	TMEM72-AS1
TALAM1	TMEM72-AS1
SLMO2-ATP5E	TMEM72-AS1
RASSF1	TMEM72-AS1

PWAR1	TMEM72-AS1
NUAK2	TMEM72-AS1
MXD1	TMEM72-AS1
MIR600HG	TMEM72-AS1
MIR22HG	TMEM72-AS1
MIR133A1HG	TMEM72-AS1
MEG9	TMEM72-AS1
MEG8	TMEM72-AS1
LINC02241	TMEM72-AS1
LINC01920	TMEM72-AS1
LINC01852	TMEM72-AS1
LINC01647	TMEM72-AS1
LINC01419	TMEM72-AS1
LINC01341	TMEM72-AS1
LINC01137	TMEM72-AS1
LINC01088	TMEM72-AS1
LINC01023	TMEM72-AS1
LINC00597	TMEM72-AS1
LINC00312	TMEM72-AS1
KCNH1-IT1	TMEM72-AS1

ITGA6-AS1	TMEM72-AS1
INO80B-WBP1	TMEM72-AS1
IDS2	TMEM72-AS1
HAS2-AS1	TMEM72-AS1
FAM230H	TMEM72-AS1
DARS-AS1	TMEM72-AS1
CYP26B1	TMEM72-AS1
C5orf17	TMEM72-AS1
C1orf140	TMEM72-AS1
C10orf62	TMEM72-AS1
BORCS7-ASMT	TMEM72-AS1
BLOC1S5-TXNDC5	TMEM72-AS1
ADAMTSL4-AS1	TMEM72-AS1
MIR133A1HG	THAP9-AS1
THAP9-AS1	TCL6
RASSF1	TCL6
NUAK2	TCL6
MIR133A1HG	TCL6
LINC01852	TCL6
LINC01647	TCL6

LINC00312	TCL6
KCNH1-IT1	TCL6
CYP26B1	TCL6
C5orf17	TCL6
BLOC1S5-TXNDC5	TCL6
XIRP1	TALAM1
TSNAX-DISC1	TALAM1
THAP9-AS1	TALAM1
TCL6	TALAM1
RASSF1	TALAM1
PWAR1	TALAM1
NUAK2	TALAM1
MIR600HG	TALAM1
MIR22HG	TALAM1
MIR133A1HG	TALAM1
MEG9	TALAM1
MEG8	TALAM1
LINC01852	TALAM1
LINC01647	TALAM1
LINC01419	TALAM1

LINC01341	TALAM1
LINC01088	TALAM1
LINC00312	TALAM1
KCNH1-IT1	TALAM1
ITGA6-AS1	TALAM1
HAS2-AS1	TALAM1
DARS-AS1	TALAM1
CYP26B1	TALAM1
C5orf17	TALAM1
BORCS7-ASMT	TALAM1
BLOC1S5-TXNDC5	TALAM1
ZNF625-ZNF20	SNORC
XIRP1	SNORC
TSNAX-DISC1	SNORC
TMEM72-AS1	SNORC
THAP9-AS1	SNORC
TCL6	SNORC
TALAM1	SNORC
SLMO2-ATP5E	SNORC
RASSF1	SNORC

PWAR1	SNORC
PTPN7	SNORC
NUAK2	SNORC
MXD1	SNORC
MIR7-3HG	SNORC
MIR600HG	SNORC
MIR22HG	SNORC
MIR133A1HG	SNORC
MEG9	SNORC
MEG8	SNORC
LINC02241	SNORC
LINC01970	SNORC
LINC01920	SNORC
LINC01852	SNORC
LINC01647	SNORC
LINC01419	SNORC
LINC01341	SNORC
LINC01137	SNORC
LINC01088	SNORC
LINC01023	SNORC

LINC00597	SNORC
LINC00312	SNORC
LINC00235	SNORC
KCNH1-IT1	SNORC
ITGA6-AS1	SNORC
INO80B-WBP1	SNORC
IDS2	SNORC
HAS2-AS1	SNORC
FAM230H	SNORC
DARS-AS1	SNORC
CYP26B1	SNORC
C5orf17	SNORC
C1orf140	SNORC
C10orf62	SNORC
BORCS7-ASMT	SNORC
BLOC1S5-TXNDC5	SNORC
ADAMTSL4-AS1	SNORC
ZNF625-ZNF20	SNHG9
XIRP1	SNHG9
TSNAX-DISC1	SNHG9

TMEM72-AS1	SNHG9
THAP9-AS1	SNHG9
TCL6	SNHG9
TALAM1	SNHG9
SNORC	SNHG9
SLMO2-ATP5E	SNHG9
RASSF1	SNHG9
PWAR1	SNHG9
PTPN7	SNHG9
NUAK2	SNHG9
MXD1	SNHG9
MIR7-3HG	SNHG9
MIR600HG	SNHG9
MIR22HG	SNHG9
MIR133A1HG	SNHG9
MEG9	SNHG9
MEG8	SNHG9
LINC02241	SNHG9
LINC01970	SNHG9
LINC01920	SNHG9

LINC01852	SNHG9
LINC01647	SNHG9
LINC01419	SNHG9
LINC01341	SNHG9
LINC01137	SNHG9
LINC01088	SNHG9
LINC01023	SNHG9
LINC00597	SNHG9
LINC00312	SNHG9
LINC00235	SNHG9
KCNH1-IT1	SNHG9
ITGA6-AS1	SNHG9
IPW	SNHG9
INO80B-WBP1	SNHG9
IDS2	SNHG9
HAS2-AS1	SNHG9
FAM230H	SNHG9
DARS-AS1	SNHG9
CYP26B1	SNHG9
C5orf17	SNHG9

C1orf140	SNHG9
C10orf62	SNHG9
BORCS7-ASMT	SNHG9
BLOC1S5-TXNDC5	SNHG9
ADAMTSL4-AS1	SNHG9
ZNF625-ZNF20	SLMO2-ATP5E
XIRP1	SLMO2-ATP5E
TSNAX-DISC1	SLMO2-ATP5E
THAP9-AS1	SLMO2-ATP5E
TCL6	SLMO2-ATP5E
TALAM1	SLMO2-ATP5E
RASSF1	SLMO2-ATP5E
PWAR1	SLMO2-ATP5E
NUAK2	SLMO2-ATP5E
MIR600HG	SLMO2-ATP5E
MIR22HG	SLMO2-ATP5E
MIR133A1HG	SLMO2-ATP5E
MEG9	SLMO2-ATP5E
MEG8	SLMO2-ATP5E
LINC02241	SLMO2-ATP5E

LINC01920	SLMO2-ATP5E
LINC01852	SLMO2-ATP5E
LINC01647	SLMO2-ATP5E
LINC01419	SLMO2-ATP5E
LINC01341	SLMO2-ATP5E
LINC01088	SLMO2-ATP5E
LINC00597	SLMO2-ATP5E
LINC00312	SLMO2-ATP5E
KCNH1-IT1	SLMO2-ATP5E
ITGA6-AS1	SLMO2-ATP5E
HAS2-AS1	SLMO2-ATP5E
DARS-AS1	SLMO2-ATP5E
CYP26B1	SLMO2-ATP5E
C5orf17	SLMO2-ATP5E
BORCS7-ASMT	SLMO2-ATP5E
BLOC1S5-TXNDC5	SLMO2-ATP5E
THAP9-AS1	RASSF1
NUAK2	RASSF1
MIR133A1HG	RASSF1
LINC01852	RASSF1

LINC01647	RASSF1
KCNH1-IT1	RASSF1
C5orf17	RASSF1
BLOC1S5-TXNDC5	RASSF1
THAP9-AS1	PWAR1
TCL6	PWAR1
RASSF1	PWAR1
NUAK2	PWAR1
MIR133A1HG	PWAR1
LINC01852	PWAR1
LINC01647	PWAR1
LINC00312	PWAR1
KCNH1-IT1	PWAR1
DARS-AS1	PWAR1
CYP26B1	PWAR1
C5orf17	PWAR1
BLOC1S5-TXNDC5	PWAR1
ZNF625-ZNF20	PTPN7
XIRP1	PTPN7
TSNAX-DISC1	PTPN7

TMEM72-AS1	PTPN7
THAP9-AS1	PTPN7
TCL6	PTPN7
TALAM1	PTPN7
SLMO2-ATP5E	PTPN7
RASSF1	PTPN7
PWAR1	PTPN7
NUAK2	PTPN7
MXD1	PTPN7
MIR7-3HG	PTPN7
MIR600HG	PTPN7
MIR22HG	PTPN7
MIR133A1HG	PTPN7
MEG9	PTPN7
MEG8	PTPN7
LINC02241	PTPN7
LINC01970	PTPN7
LINC01920	PTPN7
LINC01852	PTPN7
LINC01647	PTPN7

LINC01419	PTPN7
LINC01341	PTPN7
LINC01137	PTPN7
LINC01088	PTPN7
LINC01023	PTPN7
LINC00597	PTPN7
LINC00312	PTPN7
LINC00235	PTPN7
KCNH1-IT1	PTPN7
ITGA6-AS1	PTPN7
INO80B-WBP1	PTPN7
IDS2	PTPN7
HAS2-AS1	PTPN7
FAM230H	PTPN7
DARS-AS1	PTPN7
CYP26B1	PTPN7
C5orf17	PTPN7
C1orf140	PTPN7
C10orf62	PTPN7
BORCS7-ASMT	PTPN7

BLOC1S5-TXNDC5	PTPN7
ADAMTSL4-AS1	PTPN7
THAP9-AS1	NUAK2
MIR133A1HG	NUAK2
LINC01852	NUAK2
KCNH1-IT1	NUAK2
C5orf17	NUAK2
BLOC1S5-TXNDC5	NUAK2
ZNF625-ZNF20	MYHAS
XIRP1	MYHAS
TSNAX-DISC1	MYHAS
TMEM72-AS1	MYHAS
THAP9-AS1	MYHAS
TCL6	MYHAS
TALAM1	MYHAS
SNORC	MYHAS
SNHG9	MYHAS
SLMO2-ATP5E	MYHAS
RASSF1	MYHAS

PWAR1	MYHAS
PTPN7	MYHAS
NUAK2	MYHAS
MXD1	MYHAS
MIR7-3HG	MYHAS
MIR600HG	MYHAS
MIR22HG	MYHAS
MIR133A1HG	MYHAS
MEG9	MYHAS
MEG8	MYHAS
LINC02241	MYHAS
LINC01970	MYHAS
LINC01920	MYHAS
LINC01852	MYHAS
LINC01647	MYHAS
LINC01419	MYHAS
LINC01341	MYHAS
LINC01137	MYHAS
LINC01088	MYHAS
LINC01023	MYHAS

LINC00597	MYHAS
LINC00312	MYHAS
LINC00235	MYHAS
KCNH1-IT1	MYHAS
ITGA6-AS1	MYHAS
IPW	MYHAS
INO80B-WBP1	MYHAS
IDS2	MYHAS
HAS2-AS1	MYHAS
FAM230H	MYHAS
DARS-AS1	MYHAS
CYP26B1	MYHAS
C5orf17	MYHAS
C1orf140	MYHAS
C10orf62	MYHAS
BORCS7-ASMT	MYHAS
BLOC1S5-TXNDC5	MYHAS
ADAMTSL4-AS1	MYHAS
ZNF625-ZNF20	MXD1
XIRP1	MXD1

TSNAX-DISC1	MXD1
THAP9-AS1	MXD1
TCL6	MXD1
TALAM1	MXD1
SLMO2-ATP5E	MXD1
RASSF1	MXD1
PWAR1	MXD1
NUAK2	MXD1
MIR600HG	MXD1
MIR22HG	MXD1
MIR133A1HG	MXD1
MEG9	MXD1
MEG8	MXD1
LINC02241	MXD1
LINC01920	MXD1
LINC01852	MXD1
LINC01647	MXD1
LINC01419	MXD1
LINC01341	MXD1
LINC01088	MXD1

LINC01023	MXD1
LINC00597	MXD1
LINC00312	MXD1
KCNH1-IT1	MXD1
ITGA6-AS1	MXD1
IDS2	MXD1
HAS2-AS1	MXD1
DARS-AS1	MXD1
CYP26B1	MXD1
C5orf17	MXD1
BORCS7-ASMT	MXD1
BLOC1S5-TXNDC5	MXD1
ZNF625-ZNF20	MIR7-3HG
XIRP1	MIR7-3HG
TSNAX-DISC1	MIR7-3HG
TMEM72-AS1	MIR7-3HG
THAP9-AS1	MIR7-3HG
TCL6	MIR7-3HG
TALAM1	MIR7-3HG
SLMO2-ATP5E	MIR7-3HG

RASSF1	MIR7-3HG
PWAR1	MIR7-3HG
NUAK2	MIR7-3HG
MXD1	MIR7-3HG
MIR600HG	MIR7-3HG
MIR22HG	MIR7-3HG
MIR133A1HG	MIR7-3HG
MEG9	MIR7-3HG
MEG8	MIR7-3HG
LINC02241	MIR7-3HG
LINC01970	MIR7-3HG
LINC01920	MIR7-3HG
LINC01852	MIR7-3HG
LINC01647	MIR7-3HG
LINC01419	MIR7-3HG
LINC01341	MIR7-3HG
LINC01137	MIR7-3HG
LINC01088	MIR7-3HG
LINC01023	MIR7-3HG
LINC00597	MIR7-3HG

LINC00312	MIR7-3HG
LINC00235	MIR7-3HG
KCNH1-IT1	MIR7-3HG
ITGA6-AS1	MIR7-3HG
INO80B-WBP1	MIR7-3HG
IDS2	MIR7-3HG
HAS2-AS1	MIR7-3HG
FAM230H	MIR7-3HG
DARS-AS1	MIR7-3HG
CYP26B1	MIR7-3HG
C5orf17	MIR7-3HG
C1orf140	MIR7-3HG
C10orf62	MIR7-3HG
BORCS7-ASMT	MIR7-3HG
BLOC1S5-TXNDC5	MIR7-3HG
ADAMTSL4-AS1	MIR7-3HG
THAP9-AS1	MIR600HG
TCL6	MIR600HG
RASSF1	MIR600HG
PWAR1	MIR600HG

NUAK2	MIR600HG
MIR133A1HG	MIR600HG
LINC01852	MIR600HG
LINC01647	MIR600HG
LINC00312	MIR600HG
KCNH1-IT1	MIR600HG
DARS-AS1	MIR600HG
CYP26B1	MIR600HG
C5orf17	MIR600HG
BLOC1S5-TXNDC5	MIR600HG
XIRP1	MIR22HG
THAP9-AS1	MIR22HG
TCL6	MIR22HG
RASSF1	MIR22HG
PWAR1	MIR22HG
NUAK2	MIR22HG
MIR600HG	MIR22HG
MIR133A1HG	MIR22HG
MEG9	MIR22HG
MEG8	MIR22HG

LINC01852	MIR22HG
LINC01647	MIR22HG
LINC00312	MIR22HG
KCNH1-IT1	MIR22HG
ITGA6-AS1	MIR22HG
DARS-AS1	MIR22HG
CYP26B1	MIR22HG
C5orf17	MIR22HG
BLOC1S5-TXNDC5	MIR22HG
XIRP1	MEG9
THAP9-AS1	MEG9
TCL6	MEG9
RASSF1	MEG9
PWAR1	MEG9
NUAK2	MEG9
MIR600HG	MEG9
MIR133A1HG	MEG9
MEG8	MEG9
LINC01852	MEG9
LINC01647	MEG9

LINC00312	MEG9
KCNH1-IT1	MEG9
ITGA6-AS1	MEG9
DARS-AS1	MEG9
CYP26B1	MEG9
C5orf17	MEG9
BLOC1S5-TXNDC5	MEG9
XIRP1	MEG8
THAP9-AS1	MEG8
TCL6	MEG8
RASSF1	MEG8
PWAR1	MEG8
NUAK2	MEG8
MIR600HG	MEG8
MIR133A1HG	MEG8
LINC01852	MEG8
LINC01647	MEG8
LINC00312	MEG8
KCNH1-IT1	MEG8
DARS-AS1	MEG8

CYP26B1	MEG8
C5orf17	MEG8
BLOC1S5-TXNDC5	MEG8
XIRP1	LINC02241
TSNAX-DISC1	LINC02241
THAP9-AS1	LINC02241
TCL6	LINC02241
TALAM1	LINC02241
RASSF1	LINC02241
PWAR1	LINC02241
NUAK2	LINC02241
MIR600HG	LINC02241
MIR22HG	LINC02241
MIR133A1HG	LINC02241
MEG9	LINC02241
MEG8	LINC02241
LINC01852	LINC02241
LINC01647	LINC02241
LINC01419	LINC02241
LINC01341	LINC02241

LINC01088	LINC02241
LINC00597	LINC02241
LINC00312	LINC02241
KCNH1-IT1	LINC02241
ITGA6-AS1	LINC02241
HAS2-AS1	LINC02241
DARS-AS1	LINC02241
CYP26B1	LINC02241
C5orf17	LINC02241
BORCS7-ASMT	LINC02241
BLOC1S5-TXNDC5	LINC02241
ZNF625-ZNF20	LINC01970
XIRP1	LINC01970
TSNAX-DISC1	LINC01970
TMEM72-AS1	LINC01970
THAP9-AS1	LINC01970
TCL6	LINC01970
TALAM1	LINC01970
SLMO2-ATP5E	LINC01970
RASSF1	LINC01970

PWAR1	LINC01970
NUAK2	LINC01970
MXD1	LINC01970
MIR600HG	LINC01970
MIR22HG	LINC01970
MIR133A1HG	LINC01970
MEG9	LINC01970
MEG8	LINC01970
LINC02241	LINC01970
LINC01920	LINC01970
LINC01852	LINC01970
LINC01647	LINC01970
LINC01419	LINC01970
LINC01341	LINC01970
LINC01137	LINC01970
LINC01088	LINC01970
LINC01023	LINC01970
LINC00597	LINC01970
LINC00312	LINC01970
LINC00235	LINC01970

KCNH1-IT1	LINC01970
ITGA6-AS1	LINC01970
INO80B-WBP1	LINC01970
IDS2	LINC01970
HAS2-AS1	LINC01970
FAM230H	LINC01970
DARS-AS1	LINC01970
CYP26B1	LINC01970
C5orf17	LINC01970
C1orf140	LINC01970
C10orf62	LINC01970
BORCS7-ASMT	LINC01970
BLOC1S5-TXNDC5	LINC01970
ADAMTSL4-AS1	LINC01970
ZNF625-ZNF20	LINC01920
XIRP1	LINC01920
TSNAX-DISC1	LINC01920
THAP9-AS1	LINC01920
TCL6	LINC01920
TALAM1	LINC01920

RASSF1	LINC01920
PWAR1	LINC01920
NUAK2	LINC01920
MIR600HG	LINC01920
MIR22HG	LINC01920
MIR133A1HG	LINC01920
MEG9	LINC01920
MEG8	LINC01920
LINC02241	LINC01920
LINC01852	LINC01920
LINC01647	LINC01920
LINC01419	LINC01920
LINC01341	LINC01920
LINC01088	LINC01920
LINC00597	LINC01920
LINC00312	LINC01920
KCNH1-IT1	LINC01920
ITGA6-AS1	LINC01920
HAS2-AS1	LINC01920
DARS-AS1	LINC01920

CYP26B1	LINC01920
C5orf17	LINC01920
BORCS7-ASMT	LINC01920
BLOC1S5-TXNDC5	LINC01920
THAP9-AS1	LINC01852
MIR133A1HG	LINC01852
C5orf17	LINC01852
THAP9-AS1	LINC01647
NUAK2	LINC01647
MIR133A1HG	LINC01647
LINC01852	LINC01647
KCNH1-IT1	LINC01647
C5orf17	LINC01647
BLOC1S5-TXNDC5	LINC01647
XIRP1	LINC01419
THAP9-AS1	LINC01419
TCL6	LINC01419
RASSF1	LINC01419
PWAR1	LINC01419
NUAK2	LINC01419

MIR600HG	LINC01419
MIR22HG	LINC01419
MIR133A1HG	LINC01419
MEG9	LINC01419
MEG8	LINC01419
LINC01852	LINC01419
LINC01647	LINC01419
LINC00312	LINC01419
KCNH1-IT1	LINC01419
ITGA6-AS1	LINC01419
DARS-AS1	LINC01419
CYP26B1	LINC01419
C5orf17	LINC01419
BLOC1S5-TXNDC5	LINC01419
XIRP1	LINC01341
TSNAX-DISC1	LINC01341
THAP9-AS1	LINC01341
TCL6	LINC01341
RASSF1	LINC01341
PWAR1	LINC01341

NUAK2	LINC01341
MIR600HG	LINC01341
MIR22HG	LINC01341
MIR133A1HG	LINC01341
MEG9	LINC01341
MEG8	LINC01341
LINC01852	LINC01341
LINC01647	LINC01341
LINC01419	LINC01341
LINC01088	LINC01341
LINC00312	LINC01341
KCNH1-IT1	LINC01341
ITGA6-AS1	LINC01341
HAS2-AS1	LINC01341
DARS-AS1	LINC01341
CYP26B1	LINC01341
C5orf17	LINC01341
BORCS7-ASMT	LINC01341
BLOC1S5-TXNDC5	LINC01341
ZNF625-ZNF20	LINC01137

XIRP1	LINC01137
TSNAX-DISC1	LINC01137
THAP9-AS1	LINC01137
TCL6	LINC01137
TALAM1	LINC01137
SLMO2-ATP5E	LINC01137
RASSF1	LINC01137
PWAR1	LINC01137
NUAK2	LINC01137
MXD1	LINC01137
MIR600HG	LINC01137
MIR22HG	LINC01137
MIR133A1HG	LINC01137
MEG9	LINC01137
MEG8	LINC01137
LINC02241	LINC01137
LINC01920	LINC01137
LINC01852	LINC01137
LINC01647	LINC01137
LINC01419	LINC01137

LINC01341	LINC01137
LINC01088	LINC01137
LINC01023	LINC01137
LINC00597	LINC01137
LINC00312	LINC01137
KCNH1-IT1	LINC01137
ITGA6-AS1	LINC01137
IDS2	LINC01137
HAS2-AS1	LINC01137
DARS-AS1	LINC01137
CYP26B1	LINC01137
C5orf17	LINC01137
C1orf140	LINC01137
BORCS7-ASMT	LINC01137
BLOC1S5-TXNDC5	LINC01137
ADAMTSL4-AS1	LINC01137
XIRP1	LINC01088
THAP9-AS1	LINC01088
TCL6	LINC01088
RASSF1	LINC01088

PWAR1	LINC01088
NUAK2	LINC01088
MIR600HG	LINC01088
MIR22HG	LINC01088
MIR133A1HG	LINC01088
MEG9	LINC01088
MEG8	LINC01088
LINC01852	LINC01088
LINC01647	LINC01088
LINC01419	LINC01088
LINC00312	LINC01088
KCNH1-IT1	LINC01088
ITGA6-AS1	LINC01088
HAS2-AS1	LINC01088
DARS-AS1	LINC01088
CYP26B1	LINC01088
C5orf17	LINC01088
BORCS7-ASMT	LINC01088
BLOC1S5-TXNDC5	LINC01088
ZNF625-ZNF20	LINC01023

XIRP1	LINC01023
TSNAX-DISC1	LINC01023
THAP9-AS1	LINC01023
TCL6	LINC01023
TALAM1	LINC01023
SLMO2-ATP5E	LINC01023
RASSF1	LINC01023
PWAR1	LINC01023
NUAK2	LINC01023
MIR600HG	LINC01023
MIR22HG	LINC01023
MIR133A1HG	LINC01023
MEG9	LINC01023
MEG8	LINC01023
LINC02241	LINC01023
LINC01920	LINC01023
LINC01852	LINC01023
LINC01647	LINC01023
LINC01419	LINC01023
LINC01341	LINC01023

LINC01088	LINC01023
LINC00597	LINC01023
LINC00312	LINC01023
KCNH1-IT1	LINC01023
ITGA6-AS1	LINC01023
IDS2	LINC01023
HAS2-AS1	LINC01023
DARS-AS1	LINC01023
CYP26B1	LINC01023
C5orf17	LINC01023
BORCS7-ASMT	LINC01023
BLOC1S5-TXNDC5	LINC01023
XIRP1	LINC00597
TSNAX-DISC1	LINC00597
THAP9-AS1	LINC00597
TCL6	LINC00597
TALAM1	LINC00597
RASSF1	LINC00597
PWAR1	LINC00597
NUAK2	LINC00597

MIR600HG	LINC00597
MIR22HG	LINC00597
MIR133A1HG	LINC00597
MEG9	LINC00597
MEG8	LINC00597
LINC01852	LINC00597
LINC01647	LINC00597
LINC01419	LINC00597
LINC01341	LINC00597
LINC01088	LINC00597
LINC00312	LINC00597
KCNH1-IT1	LINC00597
ITGA6-AS1	LINC00597
HAS2-AS1	LINC00597
DARS-AS1	LINC00597
CYP26B1	LINC00597
C5orf17	LINC00597
BORCS7-ASMT	LINC00597
BLOC1S5-TXNDC5	LINC00597
THAP9-AS1	LINC00312

RASSF1	LINC00312
NUAK2	LINC00312
MIR133A1HG	LINC00312
LINC01852	LINC00312
LINC01647	LINC00312
KCNH1-IT1	LINC00312
CYP26B1	LINC00312
C5orf17	LINC00312
BLOC1S5-TXNDC5	LINC00312
ZNF625-ZNF20	LINC00235
XIRP1	LINC00235
TSNAX-DISC1	LINC00235
TMEM72-AS1	LINC00235
THAP9-AS1	LINC00235
TCL6	LINC00235
TALAM1	LINC00235
SLMO2-ATP5E	LINC00235
RASSF1	LINC00235
PWAR1	LINC00235
NUAK2	LINC00235

MXD1	LINC00235
MIR600HG	LINC00235
MIR22HG	LINC00235
MIR133A1HG	LINC00235
MEG9	LINC00235
MEG8	LINC00235
LINC02241	LINC00235
LINC01920	LINC00235
LINC01852	LINC00235
LINC01647	LINC00235
LINC01419	LINC00235
LINC01341	LINC00235
LINC01137	LINC00235
LINC01088	LINC00235
LINC01023	LINC00235
LINC00597	LINC00235
LINC00312	LINC00235
KCNH1-IT1	LINC00235
ITGA6-AS1	LINC00235
INO80B-WBP1	LINC00235

IDS2	LINC00235
HAS2-AS1	LINC00235
FAM230H	LINC00235
DARS-AS1	LINC00235
CYP26B1	LINC00235
C5orf17	LINC00235
C1orf140	LINC00235
C10orf62	LINC00235
BORCS7-ASMT	LINC00235
BLOC1S5-TXNDC5	LINC00235
ADAMTSL4-AS1	LINC00235
THAP9-AS1	KCNH1-IT1
MIR133A1HG	KCNH1-IT1
LINC01852	KCNH1-IT1
C5orf17	KCNH1-IT1
XIRP1	ITGA6-AS1
THAP9-AS1	ITGA6-AS1
TCL6	ITGA6-AS1
RASSF1	ITGA6-AS1
PWAR1	ITGA6-AS1

NUAK2	ITGA6-AS1
MIR600HG	ITGA6-AS1
MIR133A1HG	ITGA6-AS1
MEG8	ITGA6-AS1
LINC01852	ITGA6-AS1
LINC01647	ITGA6-AS1
LINC00312	ITGA6-AS1
KCNH1-IT1	ITGA6-AS1
DARS-AS1	ITGA6-AS1
CYP26B1	ITGA6-AS1
C5orf17	ITGA6-AS1
BLOC1S5-TXNDC5	ITGA6-AS1
ZNF625-ZNF20	IPW
XIRP1	IPW
TSNAX-DISC1	IPW
TMEM72-AS1	IPW
THAP9-AS1	IPW
TCL6	IPW
TALAM1	IPW
SNORC	IPW

SLMO2-ATP5E	IPW
RASSF1	IPW
PWAR1	IPW
PTPN7	IPW
NUAK2	IPW
MXD1	IPW
MIR7-3HG	IPW
MIR600HG	IPW
MIR22HG	IPW
MIR133A1HG	IPW
MEG9	IPW
MEG8	IPW
LINC02241	IPW
LINC01970	IPW
LINC01920	IPW
LINC01852	IPW
LINC01647	IPW
LINC01419	IPW
LINC01341	IPW
LINC01137	IPW

LINC01088	IPW
LINC01023	IPW
LINC00597	IPW
LINC00312	IPW
LINC00235	IPW
KCNH1-IT1	IPW
ITGA6-AS1	IPW
INO80B-WBP1	IPW
IDS2	IPW
HAS2-AS1	IPW
FAM230H	IPW
DARS-AS1	IPW
CYP26B1	IPW
C5orf17	IPW
C1orf140	IPW
C10orf62	IPW
BORCS7-ASMT	IPW
BLOC1S5-TXNDC5	IPW
ADAMTSL4-AS1	IPW
ZNF625-ZNF20	INO80B-WBP1

XIRP1	INO80B-WBP1
TSNAX-DISC1	INO80B-WBP1
THAP9-AS1	INO80B-WBP1
TCL6	INO80B-WBP1
TALAM1	INO80B-WBP1
SLMO2-ATP5E	INO80B-WBP1
RASSF1	INO80B-WBP1
PWAR1	INO80B-WBP1
NUAK2	INO80B-WBP1
MXD1	INO80B-WBP1
MIR600HG	INO80B-WBP1
MIR22HG	INO80B-WBP1
MIR133A1HG	INO80B-WBP1
MEG9	INO80B-WBP1
MEG8	INO80B-WBP1
LINC02241	INO80B-WBP1
LINC01920	INO80B-WBP1
LINC01852	INO80B-WBP1
LINC01647	INO80B-WBP1
LINC01419	INO80B-WBP1

LINC01341	INO80B-WBP1
LINC01137	INO80B-WBP1
LINC01088	INO80B-WBP1
LINC01023	INO80B-WBP1
LINC00597	INO80B-WBP1
LINC00312	INO80B-WBP1
KCNH1-IT1	INO80B-WBP1
ITGA6-AS1	INO80B-WBP1
IDS2	INO80B-WBP1
HAS2-AS1	INO80B-WBP1
DARS-AS1	INO80B-WBP1
CYP26B1	INO80B-WBP1
C5orf17	INO80B-WBP1
C1orf140	INO80B-WBP1
BORCS7-ASMT	INO80B-WBP1
BLOC1S5-TXNDC5	INO80B-WBP1
ADAMTSL4-AS1	INO80B-WBP1
ZNF625-ZNF20	IDS2
XIRP1	IDS2
TSNAX-DISC1	IDS2

THAP9-AS1	IDS2
TCL6	IDS2
TALAM1	IDS2
SLMO2-ATP5E	IDS2
RASSF1	IDS2
PWAR1	IDS2
NUAK2	IDS2
MIR600HG	IDS2
MIR22HG	IDS2
MIR133A1HG	IDS2
MEG9	IDS2
MEG8	IDS2
LINC02241	IDS2
LINC01920	IDS2
LINC01852	IDS2
LINC01647	IDS2
LINC01419	IDS2
LINC01341	IDS2
LINC01088	IDS2
LINC00597	IDS2

LINC00312	IDS2
KCNH1-IT1	IDS2
ITGA6-AS1	IDS2
HAS2-AS1	IDS2
DARS-AS1	IDS2
CYP26B1	IDS2
C5orf17	IDS2
BORCS7-ASMT	IDS2
BLOC1S5-TXNDC5	IDS2
XIRP1	HAS2-AS1
THAP9-AS1	HAS2-AS1
TCL6	HAS2-AS1
RASSF1	HAS2-AS1
PWAR1	HAS2-AS1
NUAK2	HAS2-AS1
MIR600HG	HAS2-AS1
MIR22HG	HAS2-AS1
MIR133A1HG	HAS2-AS1
MEG9	HAS2-AS1
MEG8	HAS2-AS1

LINC01852	HAS2-AS1
LINC01647	HAS2-AS1
LINC01419	HAS2-AS1
LINC00312	HAS2-AS1
KCNH1-IT1	HAS2-AS1
ITGA6-AS1	HAS2-AS1
DARS-AS1	HAS2-AS1
CYP26B1	HAS2-AS1
C5orf17	HAS2-AS1
BORCS7-ASMT	HAS2-AS1
BLOC1S5-TXNDC5	HAS2-AS1
ZNF625-ZNF20	FAM230H
XIRP1	FAM230H
TSNAX-DISC1	FAM230H
THAP9-AS1	FAM230H
TCL6	FAM230H
TALAM1	FAM230H
SLMO2-ATP5E	FAM230H
RASSF1	FAM230H
PWAR1	FAM230H

NUAK2	FAM230H
MXD1	FAM230H
MIR600HG	FAM230H
MIR22HG	FAM230H
MIR133A1HG	FAM230H
MEG9	FAM230H
MEG8	FAM230H
KCNH1-IT1	FAM230H
ITGA6-AS1	FAM230H
INO80B-WBP1	FAM230H
IDS2	FAM230H
HAS2-AS1	FAM230H
DARS-AS1	FAM230H
CYP26B1	FAM230H
C5orf17	FAM230H
C1orf140	FAM230H
C10orf62	FAM230H
BORCS7-ASMT	FAM230H
BLOC1S5-TXNDC5	FAM230H
ADAMTSL4-AS1	FAM230H

THAP9-AS1	DARS-AS1
TCL6	DARS-AS1
RASSF1	DARS-AS1
NUAK2	DARS-AS1
MIR133A1HG	DARS-AS1
LINC01852	DARS-AS1
LINC01647	DARS-AS1
LINC00312	DARS-AS1
KCNH1-IT1	DARS-AS1
CYP26B1	DARS-AS1
C5orf17	DARS-AS1
BLOC1S5-TXNDC5	DARS-AS1
THAP9-AS1	CYP26B1
RASSF1	CYP26B1
NUAK2	CYP26B1
MIR133A1HG	CYP26B1
LINC01852	CYP26B1
LINC01647	CYP26B1
KCNH1-IT1	CYP26B1
C5orf17	CYP26B1

BLOC1S5-TXNDC5	CYP26B1
THAP9-AS1	C5orf17
MIR133A1HG	C5orf17
ZNF625-ZNF20	C1orf140
XIRP1	C1orf140
TSNAX-DISC1	C1orf140
THAP9-AS1	C1orf140
TCL6	C1orf140
TALAM1	C1orf140
SLMO2-ATP5E	C1orf140
RASSF1	C1orf140
PWAR1	C1orf140
NUAK2	C1orf140
MXD1	C1orf140
MIR600HG	C1orf140
MIR22HG	C1orf140
MIR133A1HG	C1orf140
MEG9	C1orf140
MEG8	C1orf140
LINC02241	C1orf140

LINC01920	C1orf140
LINC01852	C1orf140
LINC01647	C1orf140
LINC01419	C1orf140
LINC01341	C1orf140
LINC01088	C1orf140
LINC01023	C1orf140
LINC00597	C1orf140
LINC00312	C1orf140
KCNH1-IT1	C1orf140
ITGA6-AS1	C1orf140
IDS2	C1orf140
HAS2-AS1	C1orf140
DARS-AS1	C1orf140
CYP26B1	C1orf140
C5orf17	C1orf140
BORCS7-ASMT	C1orf140
BLOC1S5-TXNDC5	C1orf140
ADAMTSL4-AS1	C1orf140
ZNF625-ZNF20	C10orf62

XIRP1	C10orf62
TSNAX-DISC1	C10orf62
THAP9-AS1	C10orf62
TCL6	C10orf62
TALAM1	C10orf62
SLMO2-ATP5E	C10orf62
RASSF1	C10orf62
PWAR1	C10orf62
NUAK2	C10orf62
MXD1	C10orf62
MIR600HG	C10orf62
MIR22HG	C10orf62
MIR133A1HG	C10orf62
MEG9	C10orf62
MEG8	C10orf62
LINC02241	C10orf62
LINC01920	C10orf62
LINC01852	C10orf62
LINC01647	C10orf62
LINC01419	C10orf62

LINC01341	C10orf62
LINC01137	C10orf62
LINC01088	C10orf62
LINC01023	C10orf62
LINC00597	C10orf62
LINC00312	C10orf62
KCNH1-IT1	C10orf62
ITGA6-AS1	C10orf62
INO80B-WBP1	C10orf62
IDS2	C10orf62
HAS2-AS1	C10orf62
DARS-AS1	C10orf62
CYP26B1	C10orf62
C5orf17	C10orf62
C1orf140	C10orf62
BORCS7-ASMT	C10orf62
BLOC1S5-TXNDC5	C10orf62
ADAMTSL4-AS1	C10orf62
XIRP1	BORCS7-ASMT
THAP9-AS1	BORCS7-ASMT

TCL6	BORCS7-ASMT
RASSF1	BORCS7-ASMT
PWAR1	BORCS7-ASMT
NUAK2	BORCS7-ASMT
MIR600HG	BORCS7-ASMT
MIR22HG	BORCS7-ASMT
MIR133A1HG	BORCS7-ASMT
MEG9	BORCS7-ASMT
MEG8	BORCS7-ASMT
LINC01852	BORCS7-ASMT
LINC01647	BORCS7-ASMT
LINC01419	BORCS7-ASMT
LINC00312	BORCS7-ASMT
KCNH1-IT1	BORCS7-ASMT
ITGA6-AS1	BORCS7-ASMT
DARS-AS1	BORCS7-ASMT
CYP26B1	BORCS7-ASMT
C5orf17	BORCS7-ASMT
BLOC1S5-TXNDC5	BORCS7-ASMT
THAP9-AS1	BLOC1S5-TXNDC5

MIR133A1HG	BLOC1S5-TXNDC5
LINC01852	BLOC1S5-TXNDC5
KCNH1-IT1	BLOC1S5-TXNDC5
C5orf17	BLOC1S5-TXNDC5
ZNF625-ZNF20	ADAMTSL4-AS1
XIRP1	ADAMTSL4-AS1
THAP9-AS1	ADAMTSL4-AS1
TCL6	ADAMTSL4-AS1
TALAM1	ADAMTSL4-AS1
SLMO2-ATP5E	ADAMTSL4-AS1
RASSF1	ADAMTSL4-AS1
PWAR1	ADAMTSL4-AS1
NUAK2	ADAMTSL4-AS1
MXD1	ADAMTSL4-AS1
MIR600HG	ADAMTSL4-AS1
MIR22HG	ADAMTSL4-AS1
MIR133A1HG	ADAMTSL4-AS1
MEG9	ADAMTSL4-AS1

MEG8	ADAMTSL4-AS1
LINC02241	ADAMTSL4-AS1
LINC01920	ADAMTSL4-AS1
LINC01852	ADAMTSL4-AS1
LINC01647	ADAMTSL4-AS1
LINC01419	ADAMTSL4-AS1
LINC01341	ADAMTSL4-AS1
LINC01088	ADAMTSL4-AS1
LINC01023	ADAMTSL4-AS1
LINC00597	ADAMTSL4-AS1
LINC00312	ADAMTSL4-AS1
KCNH1-IT1	ADAMTSL4-AS1
ITGA6-AS1	ADAMTSL4-AS1
IDS2	ADAMTSL4-AS1
HAS2-AS1	ADAMTSL4-AS1
DARS-AS1	ADAMTSL4-AS1
CYP26B1	ADAMTSL4-AS1
C5orf17	ADAMTSL4-AS1
BORCS7-ASMT	ADAMTSL4-AS1
BLOC1S5-TXNDC5	ADAMTSL4-AS1
