# Supplementary material for: Long non-coding RNA SNHG9 regulates viral replication in rhabdomyosarcoma cells infected with enterovirus D68 via miR-150-5p/c-Fos axis
Source: Front Microbiol. 2023 Jan 19;13:1081237. doi: 10.3389/fmicb.2022.1081237 (PMC9893417; doi:10.3389/fmicb.2022.1081237)
Supplement: Supplementary file 4 [file Data_Sheet_4.PDF]

frontiers

# *Supplementary Material*

**Data Sheet 4 lncRNA-mRNA direct binding dataset**

| Query | Length_Query | Target | Length_Target | dG | ndG | Start_Position_Query | End_Position_Query | Start_Position_Target | End_Position_Target |
|---|---|---|---|---|---|---|---|---|---|
| LINC00235 | 561 | SOX15 | 1394 | -68.92 | -0.1251 | 1 | 561 | 791 | 1351 |
| LINC00235 | 561 | RPL3L | 1547 | -61.48 | -0.1106 | 1 | 561 | 105 | 665 |
| LINC00235 | 561 | TSPEAR | 3980 | -65.53 | -0.1205 | 1 | 561 | 1479 | 2039 |
| LINC00235 | 561 | RASSF1 | 1979 | -80.08 | -0.1467 | 1 | 561 | 89 | 649 |
| LINC00235 | 561 | IFNL1 | 856 | -56.8 | -0.1046 | 1 | 561 | 108 | 668 |
| LINC00235 | 561 | KCNF1 | 2304 | -89.77 | -0.1851 | 1 | 497 | 1808 | 2304 |
| LINC00235 | 561 | EGR1 | 3136 | -69.45 | -0.1 | 1 | 561 | 299 | 859 |

| | | | | | | | | | |
|---|---|---|---|---|---|---|---|---|---|
| | | | | | 263 | | | | |
| LINC00235 | 561 | EGR3 | 4342 | -70.81 | -0.1285 | 1 | 561 | 3430 | 3990 |
| LINC00235 | 561 | SPATA25 | 752 | -64.81 | -0.1181 | 1 | 561 | 157 | 717 |
| LINC00235 | 561 | JUN | 3338 | -78.52 | -0.1405 | 1 | 561 | 2621 | 3181 |
| LINC00235 | 561 | B3GALT4 | 1704 | -71.91 | -0.1319 | 1 | 561 | 608 | 1168 |
| LINC00235 | 561 | GRK7 | 1746 | -62.48 | -0.1136 | 1 | 561 | 967 | 1527 |
| LINC00235 | 561 | F8A3 | 1116 | -94.93 | -0.1761 | 1 | 561 | 435 | 995 |
| LINC00235 | 561 | F8A2 | 1116 | -94.93 | -0.1761 | 1 | 561 | 435 | 995 |
| LINC00235 | 561 | YPEL2 | 5242 | -52.75 | -0.1053 | 1 | 508 | 4735 | 5242 |

| | | | | | | | | | |
|---|---|---|---|---|---|---|---|---|---|
| LINC00235 | 561 | FZD9 | 2342 | -83.39 | -0.1709 | 1 | 505 | 1838 | 2342 |
| LINC00235 | 561 | PRTN3 | 1001 | -74.92 | -0.138 | 1 | 561 | 209 | 769 |
| LINC00235 | 561 | CD68 | 1872 | -58.12 | -0.1057 | 1 | 561 | 1049 | 1609 |
| LINC00235 | 561 | RPP25 | 2333 | -89.63 | -0.1682 | 1 | 561 | 1285 | 1845 |
| LINC00235 | 561 | CTGF | 2358 | -78.51 | -0.1459 | 1 | 561 | 1741 | 2301 |
| LINC00235 | 561 | ZNF442 | 2825 | -55.39 | -0.1043 | 1 | 561 | 183 | 743 |
| LINC00235 | 561 | RGS9BP | 2894 | -81.17 | -0.1484 | 1 | 561 | 592 | 1152 |
| LINC00235 | 561 | TENT5A | 5617 | -78.43 | -0.1447 | 1 | 561 | 4907 | 5467 |
| LINC00235 | 561 | FOXD4L6 | 2725 | -76.25 | -0.1374 | 1 | 561 | 196 | 756 |

| | | | | | | | | | |
|---|---|---|---|---|---|---|---|---|---|
| LINC00235 | 561 | FOXD4L1 | 2067 | -72.83 | -0.1339 | 1 | 561 | 618 | 1178 |
| LINC00235 | 561 | COL28A1 | 3515 | -60.81 | -0.1102 | 1 | 561 | 1696 | 2256 |
| LINC00235 | 561 | CCR10 | 1244 | -80.4 | -0.1467 | 1 | 561 | 478 | 1038 |
| LINC00235 | 561 | NPC1L1 | 5066 | -65.31 | -0.1207 | 1 | 561 | 376 | 936 |
| LINC00235 | 561 | PPP1R15A | 2399 | -64.53 | -0.132 | 32 | 561 | 1 | 530 |
| LINC00235 | 561 | SLC5A5 | 3594 | -77.96 | -0.1493 | 1 | 530 | 3065 | 3594 |
| LINC00235 | 561 | DHRS2 | 1709 | -68.57 | -0.1247 | 1 | 561 | 709 | 1269 |
| LINC00235 | 561 | FOSB | 3776 | -70.69 | -0.1299 | 1 | 561 | 1703 | 2263 |
| LINC00235 | 561 | HSPA1B | 2551 | -73.23 | -0.1331 | 1 | 561 | 670 | 1230 |

| | | | | | | | | | |
|---|---|---|---|---|---|---|---|---|---|
| LINC00235 | 561 | PLA2G4B | 2752 | -65.95 | -0.1235 | 1 | 561 | 1635 | 2195 |
| LINC00235 | 561 | CA9 | 1561 | -70.48 | -0.1308 | 1 | 561 | 600 | 1160 |
| LINC00235 | 561 | HBA2 | 622 | -65.95 | -0.1397 | 1 | 480 | 143 | 622 |
| LINC00235 | 561 | HS3ST5 | 2744 | -48.75 | -0.1001 | 20 | 561 | 1 | 542 |
| LINC00235 | 561 | ZNF709 | 4916 | -37.98 | -0.1376 | 1 | 350 | 4567 | 4916 |
| LINC00235 | 561 | ASPRV1 | 2216 | -67.28 | -0.1246 | 1 | 561 | 1356 | 1916 |
| LINC00235 | 561 | ARRDC4 | 4074 | -79.67 | -0.1915 | 1 | 439 | 3636 | 4074 |
| LINC00235 | 561 | RIMBP3C | 5802 | -89.42 | -0.1746 | 1 | 561 | 5086 | 5646 |
| LINC00235 | 561 | ST6GALNAC2 | 2105 | -73.59 | -0.1628 | 1 | 476 | 1630 | 2105 |

| | | | | | | | | | |
|---|---|---|---|---|---|---|---|---|---|
| LINC00235 | 561 | HBEGF | 2381 | -61.09 | -0.1267 | 1 | 496 | 1886 | 2381 |
| LINC00235 | 561 | HSPA1A | 2445 | -73.23 | -0.1331 | 1 | 561 | 697 | 1257 |
| LINC00235 | 561 | ENPP6 | 3936 | -61.56 | -0.117 | 1 | 561 | 2477 | 3037 |
| LINC00235 | 561 | LCP1 | 3808 | -56.31 | -0.102 | 1 | 561 | 996 | 1556 |
| LINC00235 | 561 | KLF15 | 2539 | -74.16 | -0.1407 | 1 | 561 | 1185 | 1745 |
| LINC00235 | 561 | CYR61 | 2295 | -69.36 | -0.1257 | 1 | 561 | 72 | 632 |
| LINC00235 | 561 | PIM1 | 2751 | -83.88 | -0.1531 | 1 | 561 | 2046 | 2606 |
| LINC00235 | 561 | GADD45G | 1087 | -77.25 | -0.1389 | 1 | 561 | 216 | 776 |
| LINC00235 | 561 | ZNF367 | 3714 | -103.96 | -0.194 | 1 | 561 | 2880 | 3440 |

| | | | | | | | | | |
|---|---|---|---|---|---|---|---|---|---|
| LINC00235 | 561 | C11orf96 | 1361 | -89.31 | -0.163 | 1 | 561 | 688 | 1248 |
| LINC00235 | 561 | CFP | 1785 | -66.51 | -0.1209 | 1 | 561 | 727 | 1287 |
| LINC00235 | 561 | IQCN | 3860 | -71.49 | -0.1334 | 1 | 561 | 2703 | 3263 |
| LINC00235 | 561 | MEF2B | 1472 | -70.49 | -0.1263 | 1 | 561 | 159 | 719 |
| LINC00235 | 561 | JPH4 | 4404 | -90.54 | -0.1617 | 1 | 561 | 1373 | 1933 |
| LINC00235 | 561 | MYO1G | 3313 | -64.27 | -0.126 | 1 | 561 | 2621 | 3181 |
| LINC00235 | 561 | FOS | 2158 | -60.16 | -0.1148 | 1 | 561 | 146 | 706 |
| LINC00235 | 561 | DUSP1 | 2040 | -78.61 | -0.1422 | 1 | 561 | 26 | 586 |
| LINC00235 | 561 | TFF3 | 1054 | -62.82 | -0.1163 | 1 | 561 | 149 | 709 |

| | | | | | | | | | |
|---|---|---|---|---|---|---|---|---|---|
| LINC00235 | 561 | FAM71A | 2332 | -61.35 | -0.1268 | 1 | 561 | 1249 | 1809 |
| LINC00235 | 561 | C10orf62 | 1264 | -59.14 | -0.1091 | 1 | 561 | 400 | 960 |
| LINC00235 | 561 | GADD45B | 1393 | -59.46 | -0.1069 | 1 | 561 | 416 | 976 |
| LINC00235 | 561 | SMAD7 | 3088 | -83.87 | -0.1498 | 1 | 561 | 298 | 858 |
| LINC00235 | 561 | SIRT4 | 1213 | -57.49 | -0.1049 | 1 | 561 | 259 | 819 |
| LINC00235 | 561 | FBXL22 | 1570 | -72.14 | -0.1359 | 1 | 561 | 806 | 1366 |
| LINC00235 | 561 | DNAJC27 | 5008 | -49.63 | -0.1241 | 1 | 412 | 4597 | 5008 |
| LINC00235 | 561 | ATP5MF-PTCD1 | 5538 | -64.77 | -0.1217 | 1 | 561 | 4146 | 4706 |
| LINC00235 | 561 | KCNG4 | 2779 | -67.67 | -0.1 | 1 | 561 | 1011 | 1571 |

| | | | | | | | | | |
|---|---|---|---|---|---|---|---|---|---|
| | | | | | 246 | | | | |
| LINC00235 | 561 | SDCBP2 | 1595 | -69 | -0.1304 | 1 | 561 | 795 | 1355 |
| LINC00235 | 561 | PTPN7 | 3265 | -62.8 | -0.1148 | 1 | 561 | 1331 | 1891 |
| LINC00235 | 561 | MXD1 | 5630 | -57.73 | -0.1352 | 1 | 478 | 5153 | 5630 |
| LINC00235 | 561 | KCNV2 | 2174 | -68.67 | -0.1376 | 1 | 561 | 655 | 1215 |
| LINC00235 | 561 | EID3 | 1486 | -55.47 | -0.1305 | 130 | 561 | 1 | 432 |
| LINC00235 | 561 | ID1 | 1239 | -66.63 | -0.1267 | 1 | 561 | 122 | 682 |
| LINC00235 | 561 | CD74 | 1506 | -66.95 | -0.1204 | 1 | 561 | 652 | 1212 |
| LINC00235 | 561 | ATF3 | 2088 | -61.97 | -0.1185 | 1 | 561 | 1411 | 1971 |

| | | | | | | | | | |
|---|---|---|---|---|---|---|---|---|---|
| LINC00235 | 561 | MAGEA10-MAGEA5 | 1873 | -53.22 | -0.1041 | 1 | 561 | 946 | 1506 |
| LINC00235 | 561 | FXYD6-FXYD2 | 1122 | -66.25 | -0.1207 | 1 | 561 | 261 | 821 |
| LINC00235 | 561 | PLK2 | 2850 | -66.7 | -0.1266 | 13 | 561 | 1 | 549 |
| LINC00235 | 561 | RIT1 | 3455 | -50.47 | -0.1022 | 10 | 561 | 1 | 552 |
| LINC00235 | 561 | SERPINE1 | 3207 | -56.1 | -0.1013 | 1 | 561 | 2454 | 3014 |
| LINC00235 | 561 | CDK5R2 | 2528 | -86.97 | -0.1561 | 1 | 561 | 1649 | 2209 |
| LINC00235 | 561 | ZFP36 | 1752 | -64.75 | -0.1158 | 1 | 561 | 380 | 940 |
| LINC00235 | 561 | ARC | 2948 | -82.32 | -0.1533 | 1 | 561 | 2105 | 2665 |
| LINC00235 | 561 | CYP26B1 | 4567 | -70.45 | -0.135 | 1 | 561 | 2506 | 3066 |

| | | | | | | | | | |
|---|---|---|---|---|---|---|---|---|---|
| LINC00235 | 561 | KLF10 | 3115 | -60.82 | -0.1114 | 1 | 561 | 2489 | 3049 |
| LINC00235 | 561 | AMOTL2 | 4991 | -79.59 | -0.1482 | 1 | 561 | 326 | 886 |
| LINC00235 | 561 | HSFX2 | 1962 | -54.86 | -0.1076 | 29 | 561 | 1 | 533 |
| LINC00235 | 561 | KLF5 | 3583 | -82.24 | -0.1509 | 1 | 561 | 3005 | 3565 |
| LINC00235 | 561 | FRMD1 | 3049 | -64.23 | -0.1179 | 1 | 561 | 1418 | 1978 |
| LINC00235 | 561 | FAM110A | 1619 | -78.97 | -0.1476 | 1 | 561 | 700 | 1260 |
| LINC00235 | 561 | PPP1R3G | 1745 | -87.21 | -0.1569 | 1 | 561 | 554 | 1114 |
| LINC00235 | 561 | TMED7-TICAM2 | 3773 | -68.86 | -0.1282 | 1 | 559 | 3215 | 3773 |
| LINC00235 | 561 | PERM1 | 3064 | -70.12 | -0.1261 | 1 | 561 | 1046 | 1606 |

| | | | | | | | | | |
|---|---|---|---|---|---|---|---|---|---|
| LINC00235 | 561 | FOXD4L4 | 1251 | -70.65 | -0.1328 | 1 | 561 | 191 | 751 |
| LINC00235 | 561 | TBC1D3D | 2065 | -68.4 | -0.1293 | 1 | 561 | 356 | 916 |
| LINC00235 | 561 | CT45A8 | 1010 | -44.56 | -0.1173 | 151 | 561 | 1 | 411 |
| LINC00235 | 561 | CT45A2 | 1010 | -44.56 | -0.1173 | 151 | 561 | 1 | 411 |
| LINC00235 | 561 | PRKCH | 3868 | -85.8 | -0.1543 | 1 | 561 | 83 | 643 |
| LINC00235 | 561 | TMEM53 | 2379 | -67.46 | -0.122 | 1 | 561 | 1100 | 1660 |
| LINC00235 | 561 | DNAJB1 | 2419 | -70.61 | -0.1398 | 45 | 561 | 1 | 517 |
| LINC00235 | 561 | HBA1 | 627 | -67.67 | -0.1434 | 1 | 480 | 148 | 627 |
| LINC00235 | 561 | ADM | 1606 | -69.03 | -0.1288 | 1 | 561 | 611 | 1171 |

| | | | | | | | | | |
|---|---|---|---|---|---|---|---|---|---|
| LINC00235 | 561 | HSPA6 | 2370 | -71.96 | -0.1403 | 1 | 561 | 1799 | 2359 |
| LINC00235 | 561 | NUAK2 | 3466 | -66.5 | -0.1207 | 1 | 561 | 1463 | 2023 |
| LINC00235 | 561 | ADAMTS1 | 4670 | -81.6 | -0.147 | 1 | 561 | 3517 | 4077 |
| LINC00235 | 561 | TMEM42 | 997 | -61.29 | -0.1199 | 1 | 523 | 475 | 997 |
| LINC00235 | 561 | KLK14 | 1022 | -68.69 | -0.1274 | 1 | 561 | 397 | 957 |
| LINC00235 | 561 | TMEM45A | 1586 | -45.32 | -0.1434 | 1 | 400 | 1187 | 1586 |
| LINC00235 | 561 | CDKN2AIP | 3604 | -67 | -0.1216 | 1 | 561 | 3013 | 3573 |
| LINC00235 | 561 | SETDB2-PHF11 | 3711 | -68.25 | -0.129 | 1 | 542 | 3170 | 3711 |
| LINC00235 | 561 | EGR2 | 2723 | -62.99 | -0.1158 | 1 | 561 | 929 | 1489 |

| | | | | | | | | | |
|---|---|---|---|---|---|---|---|---|---|
| LINC00235 | 561 | LOC107984512 | 3361 | -68.04 | -0.1306 | 1 | 561 | 2039 | 2599 |
| LINC00235 | 561 | LOC107986754 | 1149 | -76.19 | -0.1416 | 1 | 561 | 250 | 810 |
| LINC00235 | 561 | LOC105375938 | 1346 | -86.77 | -0.1583 | 1 | 561 | 277 | 837 |
| LINC00235 | 561 | LOC107984124 | 583 | -69.69 | -0.1508 | 1 | 510 | 74 | 583 |
| LINC00235 | 561 | SNORC | 2833 | -89.16 | -0.1606 | 1 | 561 | 452 | 1012 |
| LINC00235 | 561 | PHOSPHO2-KLHL23 | 4229 | -53.12 | -0.1084 | 1 | 511 | 3719 | 4229 |
| LINC00235 | 561 | LRRC14B | 2554 | -88.96 | -0.1615 | 1 | 561 | 1869 | 2429 |
| LINC00235 | 561 | RNF151 | 855 | -65.74 | -0.1236 | 1 | 561 | 272 | 832 |
| LINC00235 | 561 | XIRP1 | 6467 | -65.42 | -0.1 | 1 | 561 | 3291 | 3851 |

| | | | | | | | | | |
|---|---|---|---|---|---|---|---|---|---|
| | | | | | 211 | | | | |
| LINC00235 | 561 | OVCH1 | 7990 | -54.54 | -0.1025 | 1 | 561 | 5765 | 6325 |
| LINC00235 | 561 | GRAPL | 1313 | -59.35 | -0.112 | 1 | 561 | 126 | 686 |
| LINC00235 | 561 | IL12A | 1329 | -59.36 | -0.1097 | 1 | 561 | 34 | 594 |
| LINC00235 | 561 | LOC100128242 | 1004 | -82.34 | -0.1545 | 1 | 561 | 30 | 590 |
| LINC00235 | 561 | NUDT4B | 3650 | -66.05 | -0.1295 | 48 | 561 | 1 | 514 |
| LINC00235 | 561 | HIST2H3PS2 | 2624 | -71.49 | -0.1293 | 1 | 561 | 1 | 561 |
| LINC00235 | 561 | LOC112268237 | 1343 | -74.33 | -0.1429 | 1 | 561 | 398 | 958 |
| LINC00235 | 561 | LOC112268238 | 1355 | -71.48 | -0.1483 | 45 | 561 | 1 | 517 |

| | | | | | | | | | |
|---|---|---|---|---|---|---|---|---|---|
| LINC00235 | 561 | LOC105378193 | 1979 | -71.75 | -0.1307 | 1 | 561 | 998 | 1558 |
| LINC00235 | 561 | LOC105371932 | 1171 | -58.98 | -0.1092 | 1 | 561 | 79 | 639 |
| LINC00235 | 561 | LOC112267940 | 3166 | -64.15 | -0.1229 | 1 | 561 | 2327 | 2887 |
| LINC00235 | 561 | LOC107986353 | 3982 | -63.27 | -0.1212 | 1 | 561 | 2327 | 2887 |
| LINC00235 | 561 | LOC112268348 | 4024 | -55.24 | -0.1006 | 1 | 561 | 44 | 604 |
| EEF1E1-BLOC1S5 | 2992 | EID3 | 1486 | -39.12 | -0.1178 | 1 | 342 | 1145 | 1486 |
| EEF1E1-BLOC1S5 | 2992 | LOC107984124 | 583 | -43.79 | -0.1343 | 1 | 370 | 214 | 583 |
| RAB4B-EGLN2 | 2849 | SPATA25 | 752 | -80.89 | -0.1154 | 911 | 1662 | 1 | 752 |
| RAB4B- | 2849 | F8A3 | 1116 | -12 | -0.115 | 722 | 1837 | 1 | 1116 |

| | | | | | | | | | |
|---|---|---|---|---|---|---|---|---|---|
| EGLN2 | | | | 5.71 | | | | | |
| RAB4B-EGLN2 | 2849 | F8A2 | 1116 | -125.71 | -0.115 | 722 | 1837 | 1 | 1116 |
| RAB4B-EGLN2 | 2849 | PRTN3 | 1001 | -100.15 | -0.1041 | 941 | 1941 | 1 | 1001 |
| RAB4B-EGLN2 | 2849 | MLN | 572 | -53.41 | -0.10115 | 1285 | 1856 | 1 | 572 |
| RAB4B-EGLN2 | 2849 | CCR10 | 1244 | -136.23 | -0.112 | 907 | 2150 | 1 | 1244 |
| RAB4B-EGLN2 | 2849 | HBA2 | 622 | -64.57 | -0.1113 | 1333 | 1954 | 1 | 622 |
| RAB4B-EGLN2 | 2849 | HBA1 | 627 | -70.33 | -0.1186 | 1445 | 2071 | 1 | 627 |
| RAB4B-EGLN2 | 2849 | KLK14 | 1022 | -108.28 | -0.1064 | 706 | 1727 | 1 | 1022 |
| RAB4B-EGLN2 | 2849 | LOC107986754 | 1149 | -113.52 | -0.1034 | 700 | 1848 | 1 | 1149 |

| | | | | | | | | | |
|---|---|---|---|---|---|---|---|---|---|
| RAB4B-EGLN2 | 2849 | LOC107984124 | 583 | -77.27 | -0.1452 | 1101 | 1683 | 1 | 583 |
| LINC01419 | 1521 | KITLG | 5376 | -23.92 | -0.1035 | 1 | 255 | 5122 | 5376 |
| LINC01419 | 1521 | ZNF20 | 3077 | -33.76 | -0.116 | 1 | 324 | 2754 | 3077 |
| C5orf17 | 2003 | EGR1 | 3136 | -75.81 | -0.1128 | 1 | 757 | 2380 | 3136 |
| C5orf17 | 2003 | INSL6 | 711 | -34.05 | -0.1229 | 1 | 287 | 425 | 711 |
| C5orf17 | 2003 | SPATA25 | 752 | -57.4 | -0.102 | 1 | 621 | 132 | 752 |
| C5orf17 | 2003 | MLN | 572 | -46.83 | -0.1003 | 1 | 474 | 99 | 572 |
| C5orf17 | 2003 | TENT5A | 5617 | -69.85 | -0.1211 | 1 | 641 | 4977 | 5617 |
| C5orf17 | 2003 | HBA2 | 622 | -60.45 | -0.1107 | 1 | 574 | 49 | 622 |

| | | | | | | | | | |
|---|---|---|---|---|---|---|---|---|---|
| C5orf17 | 2003 | HBEGF | 2381 | -59.87 | -0.1686 | 1 | 360 | 2022 | 2381 |
| C5orf17 | 2003 | ZNF367 | 3714 | -84.71 | -0.1231 | 1 | 698 | 3017 | 3714 |
| C5orf17 | 2003 | MXD1 | 5630 | -62.43 | -0.1617 | 1 | 396 | 5235 | 5630 |
| C5orf17 | 2003 | TMED7-TICAM2 | 3773 | -58.6 | -0.1422 | 1 | 497 | 3277 | 3773 |
| C5orf17 | 2003 | GAGE12F | 598 | -45.54 | -0.1095 | 1 | 450 | 149 | 598 |
| C5orf17 | 2003 | TMEM42 | 997 | -54.51 | -0.1359 | 1 | 412 | 586 | 997 |
| C5orf17 | 2003 | CDKN2AIP | 3604 | -66.96 | -0.1572 | 1 | 464 | 3141 | 3604 |
| C5orf17 | 2003 | SETDB2-PHF11 | 3711 | -55.98 | -0.1642 | 1 | 354 | 3358 | 3711 |
| C5orf17 | 2003 | LOC1079841 24 | 583 | -62.54 | -0.1292 | 1 | 514 | 70 | 583 |

| | | | | | | | | | |
|---|---|---|---|---|---|---|---|---|---|
| C5orf17 | 2003 | NUDT4B | 3650 | -65.38 | -0.1572 | 1 | 417 | 3234 | 3650 |
| C5orf17 | 2003 | LOC112268238 | 1355 | -69.29 | -0.1578 | 1 | 454 | 902 | 1355 |
| KCNH1-IT1 | 935 | ZNF20 | 3077 | -32.14 | -0.102 | 1 | 344 | 2734 | 3077 |
| KCNH1-IT1 | 935 | LOC107984124 | 583 | -41.79 | -0.1255 | 1 | 347 | 237 | 583 |
| MIR22HG | 2003 | LOC107984124 | 2003 | 2003 | 2003 | 2003 | 2003 | 2003 | 2003 |
| MIR22HG | 2003 | NUDT4B | 2003 | 2003 | 2003 | 2003 | 2003 | 2003 | 2003 |
| MIR22HG | 2003 | MXD1 | 2003 | 2003 | 2003 | 2003 | 2003 | 2003 | 2003 |
| MIR22HG | 935 | INSL6 | 935 | 935 | 935 | 935 | 935 | 935 | 935 |
| MIR22HG | 935 | ZNF563 | 935 | 935 | 935 | 935 | 935 | 935 | 935 |
| MIR22HG | 935 | ZNF442 | 935 | 935 | 935 | 935 | 935 | 935 | 935 |
