# Supplementary material for: Long non-coding RNA SNHG9 regulates viral replication in rhabdomyosarcoma cells infected with enterovirus D68 via miR-150-5p/c-Fos axis
Source: Front Microbiol. 2023 Jan 19;13:1081237. doi: 10.3389/fmicb.2022.1081237 (PMC9893417; doi:10.3389/fmicb.2022.1081237)
Supplement: Supplementary file 5 [file Data_Sheet_5.PDF]

Supplementary Material

Data Sheet 5 lncRNA-RBP datasets

RBP	lncRNA
ELAVL2	PWAR1
NOVA2	PWAR1
ZFP36	PWAR1
A2BP1	PWAR1
SFRS7	PWAR1
SFRS2	PWAR1
ZRANB2	PWAR1
NCL	PWAR1
KHDRBS3	PWAR1
SFRS1	PWAR1
QKI	PWAR1
NONO	PWAR1
PABPC1	PWAR1
RBMY1A1	PWAR1
EIF4B	PWAR1
SNRPA	PWAR1
FUS	PWAR1

ACO1	PWAR1
HNRNPA1	PWAR1
KHSRP	PWAR1
SFRS9	PWAR1
MBNL1	PWAR1
PTBP1	PWAR1
YTHDC1	PWAR1
RBMX	PWAR1
YBX1	PWAR1
SFRS13A	PWAR1
ELAVL1	PWAR1
RBM4	PWAR1
EIF4B	LINC00235
NONO	LINC00235
SFRS2	LINC00235
RBMY1A1	LINC00235
SNRPA	LINC00235
SFRS1	LINC00235
FUS	LINC00235
NCL	LINC00235

SFRS9	LINC00235
KHSRP	LINC00235
SFRS7	LINC00235
MBNL1	LINC00235
A2BP1	LINC00235
YTHDC1	LINC00235
ACO1	LINC00235
YBX1	LINC00235
RBMX	LINC00235
ZRANB2	LINC00235
HNRNPA1	LINC00235
RBM4	LINC00235
PTBP1	LINC00235
PABPC1	LINC00235
SFRS13A	LINC00235
KHDRBS3	LINC00235
QKI	LINC00235
ELAVL1	LINC00235
ZFP36	LINC02035
ELAVL2	LINC02035
QKI	LINC02035

SNRPA	LINC02035
SFRS7	LINC02035
SFRS2	LINC02035
A2BP1	LINC02035
NOVA2	LINC02035
NCL	LINC02035
HNRNPA1	LINC02035
IGF2BP1	LINC02035
KHSRP	LINC02035
EIF4B	LINC02035
SFRS1	LINC02035
NONO	LINC02035
PABPC1	LINC02035
KHDRBS3	LINC02035
RBMY1A1	LINC02035
ZRANB2	LINC02035
FUS	LINC02035
SFRS9	LINC02035
ACO1	LINC02035
PTBP1	LINC02035

MBNL1	LINC02035
YBX1	LINC02035
YTHDC1	LINC02035
RBMX	LINC02035
SFRS13A	LINC02035
ELAVL1	LINC02035
RBM4	LINC02035
NOVA2	MIR22HG
NCL	MIR22HG
ZFP36	MIR22HG
ELAVL2	MIR22HG
A2BP1	MIR22HG
SFRS2	MIR22HG
SNRPA	MIR22HG
SFRS1	MIR22HG
KHSRP	MIR22HG
NONO	MIR22HG
PABPC1	MIR22HG
RBMY1A1	MIR22HG
EIF4B	MIR22HG
QKI	MIR22HG

FUS	MIR22HG
SFRS9	MIR22HG
KHDRBS3	MIR22HG
ACO1	MIR22HG
ZRANB2	MIR22HG
MBNL1	MIR22HG
HNRNPA1	MIR22HG
YBX1	MIR22HG
YTHDC1	MIR22HG
PTBP1	MIR22HG
RBMX	MIR22HG
SFRS13A	MIR22HG
RBM4	MIR22HG
ELAVL1	MIR22HG
ZFP36	EEF1E1-BLOC1S5
NCL	EEF1E1-BLOC1S5
ELAVL2	EEF1E1-BLOC1S5
A2BP1	EEF1E1-BLOC1S5

SNRPA	EEF1E1-BLOC1S5
SFRS1	EEF1E1-BLOC1S5
SFRS2	EEF1E1-BLOC1S5
IGF2BP1	EEF1E1-BLOC1S5
EIF4B	EEF1E1-BLOC1S5
NOVA2	EEF1E1-BLOC1S5
NONO	EEF1E1-BLOC1S5
PABPC1	EEF1E1-BLOC1S5
RBMY1A1	EEF1E1-BLOC1S5
QKI	EEF1E1-BLOC1S5
ZRANB2	EEF1E1-BLOC1S5
KHDRBS3	EEF1E1-BLOC1S5
FUS	EEF1E1-BLOC1S5
HNRNPA1	EEF1E1-BLOC1S5

ACO1	EEF1E1-BLOC1S5
SFRS9	EEF1E1-BLOC1S5
MBNL1	EEF1E1-BLOC1S5
PTBP1	EEF1E1-BLOC1S5
KHSRP	EEF1E1-BLOC1S5
YTHDC1	EEF1E1-BLOC1S5
RBMX	EEF1E1-BLOC1S5
SFRS13A	EEF1E1-BLOC1S5
YBX1	EEF1E1-BLOC1S5
RBM4	EEF1E1-BLOC1S5
ELAVL1	EEF1E1-BLOC1S5
NOVA2	RAB4B-EGLN2
A2BP1	RAB4B-EGLN2
SFRS1	RAB4B-EGLN2

SNRPA	RAB4B-EGLN2
HNRNPA1	RAB4B-EGLN2
NCL	RAB4B-EGLN2
ZFP36	RAB4B-EGLN2
SFRS2	RAB4B-EGLN2
NONO	RAB4B-EGLN2
EIF4B	RAB4B-EGLN2
RBMY1A1	RAB4B-EGLN2
QKI	RAB4B-EGLN2
FUS	RAB4B-EGLN2
ACO1	RAB4B-EGLN2
SFRS7	RAB4B-EGLN2
ZRANB2	RAB4B-EGLN2
SFRS9	RAB4B-EGLN2

KHSRP	RAB4B-EGLN2
MBNL1	RAB4B-EGLN2
YBX1	RAB4B-EGLN2
PTBP1	RAB4B-EGLN2
YTHDC1	RAB4B-EGLN2
RBMX	RAB4B-EGLN2
RBM4	RAB4B-EGLN2
PABPC1	RAB4B-EGLN2
SFRS13A	RAB4B-EGLN2
ELAVL1	RAB4B-EGLN2
KHDRBS3	RAB4B-EGLN2
ZFP36	LINC01419
SNRPA	LINC01419
ELAVL2	LINC01419
SFRS7	LINC01419

ZRANB2	LINC01419
HNRNPA1	LINC01419
SFRS1	LINC01419
NOVA2	LINC01419
NONO	LINC01419
PABPC1	LINC01419
RBMY1A1	LINC01419
KHDRBS3	LINC01419
SFRS2	LINC01419
QKI	LINC01419
FUS	LINC01419
EIF4B	LINC01419
SFRS9	LINC01419
ACO1	LINC01419
MBNL1	LINC01419
NCL	LINC01419
KHSRP	LINC01419
PTBP1	LINC01419
A2BP1	LINC01419
YTHDC1	LINC01419
RBMX	LINC01419

SFRS13A	LINC01419
RBM4	LINC01419
YBX1	LINC01419
ELAVL1	LINC01419
ELAVL2	C5orf17
ZFP36	C5orf17
NOVA2	C5orf17
IGF2BP1	C5orf17
A2BP1	C5orf17
ZRANB2	C5orf17
SNRPA	C5orf17
SFRS7	C5orf17
KHSRP	C5orf17
EIF4B	C5orf17
NONO	C5orf17
SFRS2	C5orf17
SFRS1	C5orf17
PABPC1	C5orf17
RBMY1A1	C5orf17
QKI	C5orf17

KHDRBS3	C5orf17
FUS	C5orf17
SFRS9	C5orf17
ACO1	C5orf17
MBNL1	C5orf17
NCL	C5orf17
YTHDC1	C5orf17
HNRNPA1	C5orf17
PTBP1	C5orf17
YBX1	C5orf17
RBMX	C5orf17
RBM4	C5orf17
SFRS13A	C5orf17
ELAVL1	C5orf17
ELAVL2	KCNH1-IT1
ZFP36	KCNH1-IT1
QKI	KCNH1-IT1
HNRNPA1	KCNH1-IT1
KHSRP	KCNH1-IT1
EIF4B	KCNH1-IT1
SFRS2	KCNH1-IT1

SFRS7	KCNH1-IT1
NONO	KCNH1-IT1
SFRS1	KCNH1-IT1
PABPC1	KCNH1-IT1
NCL	KCNH1-IT1
SNRPA	KCNH1-IT1
RBMY1A1	KCNH1-IT1
FUS	KCNH1-IT1
KHDRBS3	KCNH1-IT1
ACO1	KCNH1-IT1
SFRS9	KCNH1-IT1
MBNL1	KCNH1-IT1
ZRANB2	KCNH1-IT1
RBMX	KCNH1-IT1
PTBP1	KCNH1-IT1
YBX1	KCNH1-IT1
YTHDC1	KCNH1-IT1
SFRS13A	KCNH1-IT1
ELAVL1	KCNH1-IT1
RBM4	KCNH1-IT1

QKI	LINC00561
SNRPA	LINC00561
ELAVL2	LINC00561
ZFP36	LINC00561
ZRANB2	LINC00561
HNRNPA1	LINC00561
KHDRBS3	LINC00561
SFRS7	LINC00561
SFRS1	LINC00561
EIF4B	LINC00561
NONO	LINC00561
PABPC1	LINC00561
RBMY1A1	LINC00561
SFRS2	LINC00561
FUS	LINC00561
SFRS9	LINC00561
ACO1	LINC00561
PTBP1	LINC00561
MBNL1	LINC00561
KHSRP	LINC00561
NCL	LINC00561

YTHDC1	LINC00561
RBMX	LINC00561
SFRS13A	LINC00561
YBX1	LINC00561
ELAVL1	LINC00561
RBM4	LINC00561
