# Supplementary material for: Long non-coding RNA SNHG9 regulates viral replication in rhabdomyosarcoma cells infected with enterovirus D68 via miR-150-5p/c-Fos axis
Source: Front Microbiol. 2023 Jan 19;13:1081237. doi: 10.3389/fmicb.2022.1081237 (PMC9893417; doi:10.3389/fmicb.2022.1081237)
Supplement: Supplementary file 6 [file Data_Sheet_6.PDF]

Supplementary Material

Data Sheet 6 The set of genes for enrichment analysis

Target
SOX15
RPL3L
TSPEAR
RASSF1
IFNL1
KCNF1
EGR1
EGR3
SPATA25
JUN
B3GALT4
GRK7
F8A3
F8A2
YPEL2
FZD9
PRTN3

CD68
RPP25
CTGF
ZNF442
RGS9BP
TENT5A
FOXD4L6
FOXD4L1
COL28A1
CCR10
NPC1L1
PPP1R15A
SLC5A5
DHRS2
FOSB
HSPA1B
PLA2G4B
CA9
HBA2
HS3ST5

ZNF709
ASPRV1
ARRDC4
RIMBP3C
ST6GALNAC2
HBEGF
HSPA1A
ENPP6
LCP1
KLF15
CYR61
PIM1
GADD45G
ZNF367
C11orf96
CFP
IQCN
MEF2B
JPH4
MYO1G
FOS

DUSP1
TFF3
FAM71A
C10orf62
GADD45B
SMAD7
SIRT4
FBXL22
DNAJC27
ATP5MF-PTCD1
KCNG4
SDCBP2
PTPN7
MXD1
KCNV2
EID3
ID1
CD74
ATF3
MAGEA10-MAGEA5

FXYD6-FXYD2
PLK2
RIT1
SERPINE1
CDK5R2
ZFP36
ARC
CYP26B1
KLF10
AMOTL2
HSFX2
KLF5
FRMD1
FAM110A
PPP1R3G
TMED7-TICAM2
PERM1
FOXD4L4
TBC1D3D
CT45A8
CT45A2

PRKCH
TMEM53
DNAJB1
HBA1
ADM
HSPA6
NUAK2
ADAMTS1
TMEM42
KLK14
TMEM45A
CDKN2AIP
SETDB2-PHF11
EGR2
LOC107984512
LOC107986754
LOC105375938
LOC107984124
SNORC
PHOSPHO2-KLHL23

LRRC14B
RNF151
XIRP1
OVCH1
GRAPL
IL12A
LOC100128242
NUDT4B
HIST2H3PS2
LOC112268237
LOC112268238
LOC105378193
LOC105371932
LOC112267940
LOC107986353
LOC112268348
MLN
KITLG
ZNF20
INSL6
GAGE12F

ZNF563
CREBRF
ARRDC3
ZNF441
IFIT2
RSAD2
C7orf61
OAS1
ZNF433
RABGGTB
NUP210L
ZNF844
DNAI1
ID2
PDZRN4
FLRT3
IL7R
IL23A
FAM169A
SLC16A6

SGMS2
ACSL5
HTD2
ZNF625
ADAM21
PLCL2
UGT8
TRPM3
KLHL38
KIAA1549L
DAB1
CDRT4
METTL21C
PTPRC
HBB
ALDOB
HLA-DRA
SNTG1
APOL4
CLVS1
TGIF2-RAB5IF

SLC2A2
SOHLH2
