# Supplementary material for: Long non-coding RNA SNHG9 regulates viral replication in rhabdomyosarcoma cells infected with enterovirus D68 via miR-150-5p/c-Fos axis
Source: Front Microbiol. 2023 Jan 19;13:1081237. doi: 10.3389/fmicb.2022.1081237 (PMC9893417; doi:10.3389/fmicb.2022.1081237)
Supplement: Supplementary file 7 [file Data_Sheet_7.PDF]

Supplementary Material

Data Sheet 7 Pathway enrichment analysis was performed by the number of enriched genes

Term	Count	%	P-Value	Benjamini	Genes
MAPK signaling pathway	11	6.5	4.60E-04	8.80E-02	JUN, KITLG, GADD45B, DUSP1, PLA2G4B, HSPA6, FOS, PTPN7, HSPA1B, GADD45G, HSPA1A
Measles	7	4.1	2.10E-03	2.00E-01	JUN, OAS1, HSPA6, IL12A, FOS, HSPA1B, HSPA1A
Hippo signaling pathway	7	4.1	3.80E-03	2.50E-01	RASSF1, ID2, ID1, SERPINE1, FZD9, FRMD1, SMAD7
African trypanosomiasis	4	2.4	5.20E-03	2.50E-01	HBB, HBA2, IL12A, HBA1
Antigen processing and presentation	5	3	6.50E-03	2.50E-01	CD74, HSPA6, HLA-DRA, HSPA1B, HSPA1A
Estrogen signaling pathway	6	3.6	1.00E-02	3.30E-01	JUN, HSPA6, FOS, HSPA1B, HSPA1A, HBEGF
Malaria	4	2.4	1.20E-02	3.30E-01	HBB, HBA2, IL12A, HBA1
Legionellosis	4	2.4	1.70E-02	3.70E-01	HSPA6, IL12A, HSPA1B, HSPA1A
C-type lectin receptor signaling pathway	5	3	1.70E-02	3.70E-01	EGR2, JUN, EGR3, IL23A, IL12A
Herpes simplex virus 1 infection	11	6.5	1.90E-02	3.70E-01	ZNF441, ZNF709, CD74, OAS1, ZNF20, HLA-DRA, IL12A, CFP, ZNF433, ZNF442, ZNF563

Toxoplasmosis	5	3	2.20E-02	3.90E-01	HSPA6, HLA-DRA, IL12A, HSPA1B, HSPA1A
Inflammatory bowel disease	4	2.4	2.40E-02	3.90E-01	JUN, IL23A, HLA-DRA, IL12A
Amphetamine addiction	4	2.4	2.80E-02	4.00E-01	ARC, JUN, FOSB, FOS
Pathways in cancer	11	6.5	2.90E-02	4.00E-01	RASSF1, JUN, KITLG, GADD45B, IL23A, FZD9, PIM1, IL12A, FOS, IL7R, GADD45G
Pertussis	4	2.4	3.60E-02	4.50E-01	JUN, IL23A, IL12A, FOS
Leishmaniasis	4	2.4	3.70E-02	4.50E-01	JUN, HLA-DRA, IL12A, FOS
Colorectal cancer	4	2.4	4.90E-02	5.10E-01	JUN, GADD45B, FOS, GADD45G
Breast cancer	5	3	5.20E-02	5.10E-01	JUN, GADD45B, FZD9, FOS, GADD45G
Lipid and atherosclerosis	6	3.6	5.50E-02	5.10E-01	JUN, HSPA6, IL12A, FOS, HSPA1B, HSPA1A
Th1 and Th2 cell differentiation	4	2.4	5.80E-02	5.10E-01	JUN, HLA-DRA, IL12A, FOS
Rheumatoid arthritis	4	2.4	5.90E-02	5.10E-01	JUN, IL23A, HLA-DRA, FOS
GnRH signaling pathway	4	2.4	5.90E-02	5.10E-01	EGR1, JUN, PLA2G4B, HBEGF
Human T-cell leukemia virus 1 infection	6	3.6	6.10E-02	5.10E-01	EGR1, ZFP36, EGR2, JUN, HLA-DRA, FOS

JAK-STAT signaling pathway	5	3	6.90E-02	5.20E-01	IFNL1, IL23A, PIM1, IL12A, IL7R
AGE-RAGE signaling pathway in diabetic complications	4	2.4	7.00E-02	5.20E-01	EGR1, JUN, SERPINE1, PIM1
Coronavirus disease - COVID-19	6	3.6	7.10E-02	5.20E-01	JUN, OAS1, IL12A, FOS, RPL3L, HBEGF
Chagas disease	4	2.4	7.40E-02	5.20E-01	JUN, SERPINE1, IL12A, FOS
Ether lipid metabolism	3	1.8	8.00E-02	5.20E-01	UGT8, PLA2G4B, ENPP6
Protein processing in endoplasmic reticulum	5	3	8.10E-02	5.20E-01	PPP1R15A, DNAJB1, HSPA6, HSPA1B, HSPA1A
Influenza A	5	3	8.10E-02	5.20E-01	DNAJB1, RSAD2, OAS1, HLA-DRA, IL12A
Th17 cell differentiation	4	2.4	8.40E-02	5.30E-01	JUN, IL23A, HLA-DRA, FOS
